# *E. coli* prepares for starvation by dramatically remodeling its proteome in the first hours after loss of nutrients

**DOI:** 10.1038/s44320-026-00226-5

**Published:** 2026-07-01

**Authors:** Théo Gervais, Bjoern Kscheschinski, Michael Mell, Nevil Goepfert, Erik van Nimwegen, Thomas Julou

**Affiliations:** 1 Biozentrum, University of Basel, Spitalstrasse 41, Basel, 4056 Switzerland; 2 Swiss Institute of Bioinformatics, Basel, Switzerland

**Keywords:** Microbiology, Virology & Host Pathogen Interaction, Proteomics

## Abstract

Although in the wild bacteria likely spend most of their time deprived of nutrients and slowly starving to death, very little is known about how bacteria adapt their phenotype to starvation. Here we combine microfluidics with quantitative fluorescence microscopy of transcriptional reporters to comprehensively quantify growth and gene expression at the single-cell level in E. coli during carbon starvation. We find that all cells immediately stop growing upon loss of carbon source and that almost all remain alive for over 30 hours. Furthermore, entry into starvation triggers a dynamic expression program that is remarkably homogeneous across single cells, but highly variable across genes, causing dramatic remodeling of the proteome early in starvation. We further show that, as protein production and the rate of protein degradation both decay approximately exponentially, protein concentrations become essentially ‘frozen’ after the first 5 to 10 hours, setting phenotypes for several days of starvation. Finally, using experiments in which gene expression is inhibited for different periods, we show that protein production in the first 5 hours is crucial for protecting cells from stresses late in starvation.

## Synopsis

Upon loss of carbon source, single *E. coli* cells immediately halt growth and undergo a homogeneous gene expression response that remodels their proteome, with protein levels becoming stable after 10 hours. This protein production early in starvation is vital for long-term stress survival.

- All cells go into growth-arrest within minutes of loss of carbon source, longer cells subsequently undergo reductive division, and cells remain alive for over 30 hours.
- Upon entry into starvation, all cells undergo a similar gene expression program that dramatically remodels their proteome.
- Both protein production rates and protein degradation rates decay approximately exponentially on a 5-10 hour time scale, causing protein levels to stabilize after 10 hours.
- This expression program early in stationary phase is crucial for tolerance to stress later in starvation.

## Abstract (web summary)

Upon loss of carbon source, single *E. coli* cells immediately halt growth and undergo a homogeneous gene expression response that remodels their proteome, with protein levels becoming stable after 10 hours. This protein production early in starvation is vital for long-term stress survival.

## Graphical abstract

**Figure:**
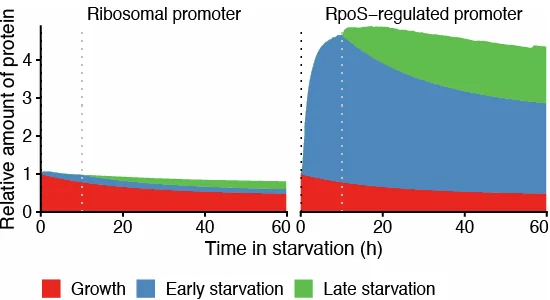

**Figure:**
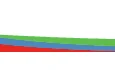

## Introduction

Most bacteriological studies in the laboratory focus on exponentially growing cells for several reasons. First, it is relatively easy to reproducibly measure the growth rates of different strains in different environments. More generally, balanced exponential growth constitutes a well-defined steady state, which guarantees that measurements do not depend on the precise timing of the experiments. Furthermore, differences in growth rate are also of obvious evolutionary importance in the wild, as faster-growing bacterial strains are able to consume available resources before other competing strains.

However, it is generally believed that in their natural environments, bacteria are often starving for nutrients and may thus likely spend most of their time not growing, and eventually dying of starvation or stresses present in the environment. Indeed, if the total bacterial population on earth remains roughly constant, the total amount of bacterial growth and death must be balanced. Hence, one would a priori expect the selection pressure for survival, and for surviving starvation in particular, to be as important as the selection for fast growth. While bacterial survival has been observed to exhibit simple exponential decay in certain conditions (Schink et al, 2019), other studies report an initial phase with almost no death (Dukan and Nyström, 1999) and different bacterial species die at different rates and with diverse dynamics (Bergkessel and Delavaine, 2021), suggesting that differences in both conditions and bacterial physiology can have a strong impact on mortality. This argues that the study of the states of bacteria in non-growing conditions, and how these affect survival rates, is as important as the study of bacteria in exponential growth conditions.

The study of the behavior of growth-arrested bacteria is also highly relevant from a medical point of view. First, various pathogenic bacterial species induce their virulence genes at the transition to the stationary phase (Bachman and Swanson, 2001; Iriarte et al, 1995; Lim et al, 2013; Sonenshein, 2005). Second, bacteria in the stationary phase have higher levels of tolerance to antibiotics (Gutierrez et al, 2017; Tuomanen, 1986; Tuomanen et al, 1986), and the fraction of persister cells is much higher in stationary phase cultures than in exponentially growing ones (Balaban et al, 2004; Harms et al, 2016).

A straightforward way to induce non-growing states in the laboratory, at least at the population level, is to simply let bacterial populations grow until they exhaust their nutrients, after which they enter the “stationary phase” where the population size initially remains constant and then decreases due to cell death. This stationary phase is therefore a practical setting for studying bacterial starvation.

Despite the importance of understanding bacterial states in the stationary phase, both from an evolutionary and medical perspective, relatively little is currently known. Previous studies of the stationary phase have revealed that, in general, cells become much less active: their energy levels sink (Charbon et al, 2021; Lempp et al, 2019; Lin and Jacobs-Wagner, 2022), their DNA becomes coated by the protein Dps, which is not expressed in growing cells (Janissen et al, 2018), and the composition of the cytoplasmic membrane and the cell wall changes to make them sturdier (Huisman et al, 1996; Schink et al, 2022). In addition, transcriptomic analysis during the stationary phase in minimal media indicates a global reallocation of the transcriptome with a major role of the transcription factor RpoS, including rapid arrest of expression of genes involved in amino acid and nucleotide synthesis (Lempp et al, 2019; Lim et al, 2025). However, it is also known that the physiological state during starvation is dependent on the physico-chemical conditions, such as the limiting nutrient, the osmolarity, pH, on how quickly bacteria transition from growth conditions to growth arrest, and on how long the bacteria have been in starvation (Bergkessel et al, 2016; Huisman et al, 1996). How all these factors determine the precise physiological state of the cells during starvation remains to be characterized.

Here, we focus on quantifying the time evolution of gene expression during the first few days of carbon starvation. In a previous study, it was reported that the expression of a transcriptional reporter of a synthetic inducible promoter exhibited constant gene expression, at ≈10% of the level in exponentially growing cells, over 50 h in the stationary phase (Gefen et al, 2014). This indicates that, at least for some genes, there is sustained gene expression activity deep into the stationary phase, but it is currently unclear to what extent this observation generalizes to different genes. Furthermore, although cell-to-cell variability is believed to be higher in the stationary phase (Bergkessel et al, 2016; Guido et al, 2007), virtually nothing is currently known about how the expression dynamics during starvation varies across single cells. We thus decided to quantify expression dynamics at the single-cell level.

To track the time evolution of single-cell growth and gene expression dynamics upon a switch from exponential growth to carbon starvation, we used a microfluidic device that allows a precisely controlled switch in environment (Kaiser et al, 2018) together with time-lapse fluorescence microscopy of transcriptional reporters for a diverse collection of *E. coli* promoters. In combination with automated image analysis, this allowed us to track many independent cell lineages and to quantify growth and gene expression for each of the promoters in each cell.

We find that all cells immediately enter growth arrest upon removal of the carbon source and remain growth arrested as long as the carbon source remains absent. Second, we find that, after going into growth arrest, cells that are further along in their cell cycle undergo reductive division, causing the average cell size of the population to decrease. In addition, we observed that almost all cells (at least 92%) survive more than two days of growth arrest in our experiments.

Using the GFP fluorescence measurements, we find that protein production rates undergo dramatic changes upon entry into starvation with a wide variety of behaviors across promoters, but a remarkable consistency across single cells. Some genes cease protein production immediately, some slowly fade in protein production on a time scale of approximately 10 h, and others show a transient increase in protein production, after which production also slowly fades away over time, decaying approximately exponentially. Using separate measurements of protein degradation, we find that the rate at which fluorescent proteins are degraded itself decays exponentially with time. Assuming that this exponential decay of the degradation rate is a global feature that applies to other proteins as well, we developed a mathematical model that infers the dynamics of protein concentration for each promoter. This model shows how protein concentrations in late starvation are determined by the combination of growth arrest (which halts dilution), the extent of transient increase in expression, the rate of exponential decay in protein production, and the exponential decay in protein degradation.

Thus, the expression program in the first hours of starvation is the main determinant of the proteome composition later in starvation.

Using a setup where a batch culture is flown through the microfluidic chip as it progresses from exponential phase to stationary phase, we also show that the gene expression response is similar between abrupt and gradual growth arrest.

Finally, we demonstrate that the transient expression upon entry into starvation has major phenotypic consequences. In particular, we show that the expression changes early in starvation are crucial for surviving stress later in starvation.

## Results

**Fig. 1:**
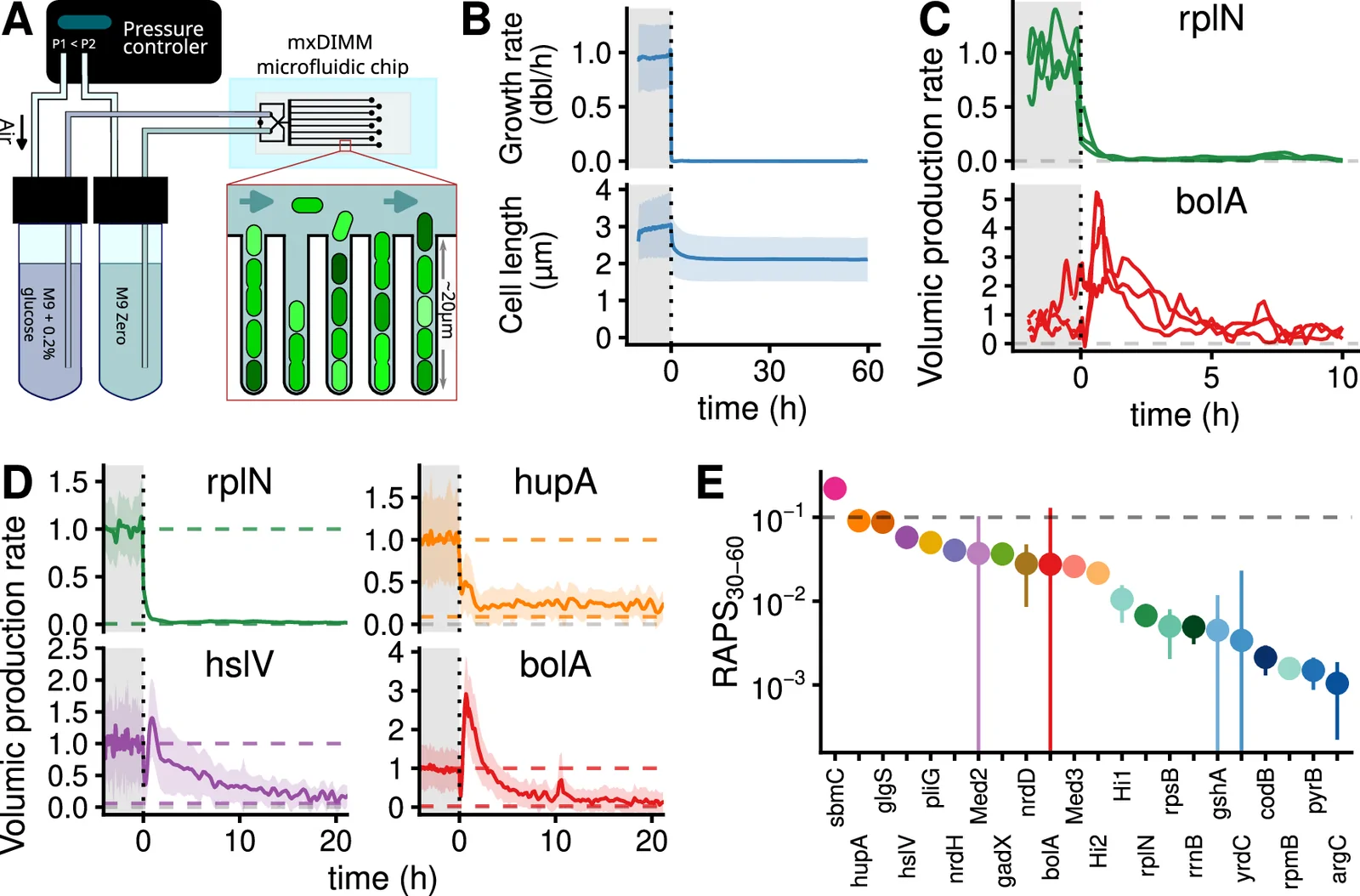
Characterization of single-cell gene expression during starvation using microfluidics and time-lapse microscopy. (**A**) Size and fluorescence of individual *E. coli* bacteria were monitored as cells were switched from exponential growth (M9 minimal media + 0.2% glucose) to starvation conditions ("M9 zero" without carbon source) in a modified DIMM microfluidic chip (Kaiser et al, 2018), allowing measurement of up to 8 strains in parallel. (**B**) After switching to “M9 zero", elongation stops immediately (top panel, Appendix Fig. S4), and average cell size decreases due to reductive divisions (bottom panel). Lines and ribbons show mean and mean ± s.d. across all single cells (at least 3500 per time point), respectively; time 0 was defined as the time of the media switch. (**C**) Examples of single-cell traces of the volumic production rate *q* for the rplN promoter (top panel) and the bolA promoter (bottom panel). The volumic production rate is defined as the amount of fluorescent protein produced from the transcriptional reporter per unit time and per unit cell volume. Note the consistency in the time-dependent response in production upon entry into starvation across the single cells. (**D**) Time-dependent volumic production rates for four example promoters (out of 22) illustrate the diversity of the responses upon entry into starvation (only the first 20 of 60 h are shown); lines and ribbons show mean and mean ± s.d. across all single cells (at least 20 to 200 per time point), respectively, normalized by the average value during exponential growth. Colored dashed lines are guides for the eye showing 1 (i.e., the production during exponential growth) and the average relative production rate in the last 30 h (relative average production in starvation between 30 and 60 h, RAPS_30–60_). (**E**) Production rates late in starvation relative to production rates in exponential growth (RAPS_30–60_) for 18 native and 4 synthetic promoters vary over more than two orders of magnitude (error bars correspond to RAPS ± s.d., calculated by Gaussian error propagation from the relevant average volumic productions; 10^4^ to 10^5^ observations per point, on 60 to 470 cells in starvation and 630 to 6000 cells in exponential growth). The horizontal dashed line indicates the value reported previously for a synthetic promoter in Gefen et al (2014).

In order to study gene expression under starvation with single-cell resolution, we used an integrated setup which combines microfluidics, time-lapse microscopy and automated image analysis (Kaiser et al, 2018). After inoculation in the dual input mother machine (DIMM) microfluidic chip, *E. coli* cells were first grown in M9 minimal media supplemented with 0.2% glucose for 10 h and then switched to M9 minimal media without a carbon source (referred to as “M9 zero”) for up to 60 h. To assess the survival of cells at the end of this starvation period, cells were then allowed to regrow in fresh media for an additional 10 to 20 h in some experiments. We used a multiplexed version of the DIMM chip that allows us to measure up to 8 strains in parallel, each carrying a different transcriptional fluorescent reporter (Fig. 1a).

### Inference of instantaneous growth and protein production rates

We first summarize our processing of the raw image data. A more thorough description of all procedures is in the Methods, and additional details are provided in the Appendix.

The cell size *V*(*t*) and total fluorescence *F*(*t*) of each cell at each time point in the experiment were estimated from the raw image data using our automated image analysis software. From this, we estimated the instantaneous growth rate *λ*(*t*) and volumic protein production rate *q*(*t*), i.e., the amount of fluorescence produced per unit time and unit cell volume, as a function of time *t* for each cell.

Formally, we define the instantaneous growth rate *λ*(*t*) of a cell as the time derivative of the logarithm of cell volume

1 $$\frac{dV(t)}{dt}=\lambda (t)V(t),$$

with *V*(*t*) the cell’s volume at time *t*. Similarly, we define the instantaneous volumic protein production *q*(*t*) as the rate at which fluorescent proteins are produced per unit time and per unit volume of the cell. That is, we assume that the total fluorescence *F*(*t*) of the cell evolves as

2 $$\frac{dF(t)}{dt}=q(t)V(t)-\left(\beta +\gamma (t)\right)F(t)$$

where *β* is the rate of photobleaching and *γ*(*t*) is the rate of degradation of fluorescent protein, which, as we will see below, depends explicitly on time *t* during starvation.

We use the volumic production rate *q*(*t*) as a proxy for the expression of the corresponding gene since it captures both the promoter-specific transcriptional activity as well as the global decrease of transcription and translation that occurs during starvation. In particular, because the fluorescent reporters of different promoters have essentially the same mRNA, the production rate *q*(*t*) is proportional to the product of the reporter mRNA concentration and the total protein production rate per mRNA.

There are several challenges in inferring the growth rates *λ*(*t*) and production rates *q*(*t*) from the raw measurements. First, the raw measurements of cell size and fluorescence are affected by significant measurement noise and, because true size changes little between consecutive time points, simply taking discrete derivatives between consecutive measurement time points of the logarithm of cell volume would result in estimates of growth rate that are far too noisy to be useful, and the same applies to inferring *q*(*t*) from changes in measured total fluorescence. To address this challenge, we recently developed an advanced Bayesian inference procedure, called RealTrace, that removes measurement noise by assuming the growth and production rate are both smoothly fluctuating with time, without making specific assumptions about the functional form of these fluctuations Kscheschinski et al. (2025), resulting in smooth traces of instantaneous growth and production rates of every cell at every time point (Appendix Fig. S1).

A second challenge is that RealTrace assumes that bleaching and protein degradation are constant in time. However, we performed calibration experiments to determine protein degradation rates (Appendix Fig. S2), and these uncovered that the protein degradation rate of the fluorescent proteins is decreasing approximately exponentially with time during the starvation phase, i.e., $\gamma (t)={\gamma }_{0}{e}^{-{\omega }_{\gamma }t}$, if we define *t* = 0 as the start of the starvation phase. Although we noted that the degradation rate and its dynamics can vary between individual cells, we considered here only the average degradation across the whole population. With this, we inferred the volumetric production rates *q*(*t*) as follows. After estimating the time-dependent degradation rates *γ*(*t*) of the fluorescent proteins, we performed additional calibration experiments to estimate the photobleaching rates *β* of the fluorescent proteins (see Methods and Appendix Table S1). We then ran RealTrace assuming fluorescence only decays through photobleaching, i.e., assuming the total fluorescence of the cell evolves according to $\frac{dF}{dt}={q}^{\inf }(t)V(t)-\beta F(t)$. Because this inference neglects protein degradation, the inferred production rate ${q}^{\inf }(t)$ will systematically underestimate the true production rate *q*(*t*). But because we have estimated the time-dependent protein degradation rate *γ*(*t*), we can correct for this underestimation to infer the true production rate *q*(*t*) (Appendix section 3).

Finally, the observed production of fluorescence lags behind the production of fluorescent proteins because fluorescent proteins need some time to mature. Most of the results in this study use GFPmut2 as a reporter, and its maturation time is sufficiently short that we can neglect maturation without substantially affecting the observed production traces (Appendix Fig. S3). However, in some experiments, we used dual-reporter strains that also use mVenus and mCherry. Since these fluorescent proteins have longer maturation times, we explicitly correct for maturation for the results with these reporters as described in Appendix section 3.

### Immediate growth arrest and reductive divisions upon entry into starvation

The most readily observable signatures of starvation are the arrest of growth at the population level, and the decrease of average cell size. Although it has been reported recently that *E. coli* cells are all growth-arrested during the stationary phase (Bakshi et al, 2021), our experiments are the first to measure the changes in growth at the single-cell level with high time resolution as cells are abruptly switched from exponential growth to carbon-free media.

We observed that, upon the switch from M9 + 0.2% glucose to M9 zero, all bacteria stop growing immediately (within the 3-min microscopy acquisition time; Fig. 1B; Appendix Fig. S4A,B). Almost all cells remain alive for more than 25 h (95 ± 5%), and the vast majority of the cells (92 ± 4%) are still able to regrow after 60 h of starvation in the absence of fluorescence illumination (Appendix Fig. S5A,B). This is in agreement with survival curves measured in batch cultures for this strain and media (Dukan and Nyström, 1999).

In addition, after growth has completely ceased, a large fraction of cells (38%) undergoes division within the first 10 h, with most of these reductive divisions occurring early and their frequency decreasing with time (Appendix Fig. S4C), leading to a decrease of the average cell size with time (Fig. 1B). That average cell size decreases upon entry into stationary phase has been observed previously (Bakshi et al, 2021; Nyström, 2004), and this has been attributed to a different mechanism of cell size control during the last cell cycle(s). However, our data show that reductive divisions occur after cell growth has already ceased completely. Moreover, we find that whether a cell will undergo a reductive division is well predicted by its size at the time of entry into starvation, i.e., as measured either by the cell’s absolute length or added cell length since birth (Appendix Fig. S4D). It is tempting to speculate that, perhaps, the cells that undergo reductive division are those in which DNA replication has already been started or completed.

In summary, in contrast to the hypothesis that the stationary phase at the population level might reflect a balance between growth and death at the single-cell level, we find that all cells immediately arrest growth upon loss of carbon source, and that the reduction in average cell size results from the fact that longer cells undergo a division during the first hours of growth arrest. Further, although it is impossible to exclude that trace amounts of carbon nutrients might still be present after the switch to M9 zero, it is clear that the immediate growth arrest in all cells is caused by the dramatic decrease in carbon availability upon the switch, and we will refer to this state as carbon starvation. Our microfluidic setup is thus a powerful platform to study this carbon starvation at high time resolution, under controlled conditions.

### Dramatic and highly consistent remodeling of gene expression upon entry into starvation

We measured gene expression dynamics during starvation using transcriptional fluorescent reporters for a set of native and synthetic promoters selected as follows. For native promoters, we took advantage of previous flow cytometry measurements (Urchueguía et al, 2021) to pick 18 promoters spanning a broad range of expression and of fold-changes between growth and stationary phase (Appendix Fig. S6). In order to quantify expression of constitutive genes, we also used four synthetic promoters that were obtained by screening random sequence fragments for their expression level during exponential growth (either “medium” or “high”) (Wolf et al, 2015), and for which we verified the absence of regulatory inputs other than sigma factor binding sites (RpoD/RpoS).

In previous studies, it has been observed that, when conditions change, different subpopulations of single cells can exhibit highly variable responses in gene expression (Galbusera et al, 2025; Jones and Uphoff, 2021; Julou et al, 2020), and we thus anticipated that the expression responses in single cells upon a switch to starvation might also exhibit large variability. However, we not only find that upon entry into starvation, the protein production rates of different promoters undergo dramatic changes that are highly diverse across promoters, but also that these changes are highly consistent across single cells. As an example, Fig. 1C shows single-cell production traces for the ribosomal protein promoter rplN and the rpoS target promoter bolA, and Appendix Fig. S7 shows example single-cell production traces for all 22 promoters.

Although we do observe substantial variability across single cells for each promoter, the consistency of the time-dependent protein production profiles across single cells motivated us to first analyze the responses across promoters based on their time-dependent means and standard deviations across the population. We will return to the analysis of the variability across single cells later on. Figure 1D shows the average volumic protein production rate across time for four native promoters representative of the diversity of the dynamics that we observe across the 22 promoters (Appendix Fig. S8A). Note that values are scaled to the average volumic production rate during exponential growth and that our set of promoters covers a range of absolute volumic production rates that spans more than two orders of magnitude during growth, and more than three orders of magnitude during starvation (Appendix Fig. S8B).

Overall, protein production rates change dramatically at the onset of starvation. As can be seen from Fig. 1D, production patterns are diverse and rather complex upon entry into starvation. Certain promoters, such as the ribosomal promoter rplN, undergo a sharp downregulation of production that almost stops their production within an hour of the switch. Other promoters, such as hupA, see a sharp initial drop in production, followed by a slower decay, and even a very long sustained production at a lower level. Finally, promoters such as hslV and bolA show a transient burst of variable amplitude in production, increasing production over its value during exponential growth, followed by a slower decay in production that proceeds at different rates for different promoters. As expected, we observed that the mean volumic production rate after prolonged starvation is always lower than it was during exponential growth.

As discussed above, protein production rates are proportional to mRNA levels, so that, we in principle expect that the time-dependent production rates of different promoters should also be evident in bulk population transcriptomic data. Although, as discussed in Appendix section 6, there are several challenges to quantitatively compare volumic protein production rates with RNA-seq data, we find that the time-dependent expression profiles of different promoters are generally reproduced in transcriptomic measurements (Appendix Fig. S9).

### Slow protein production decay at variable rates across promoters

In a previous study, it was found that one promoter exhibits approximately constant activity in the stationary phase at the population level over a 50-h period (Gefen et al, 2014). In line with this observation, we also find that several promoters still have detectable protein production 60 h after the switch to starvation, i.e., at the end of our experiments (Appendix Fig. S10B). However, rather than a constant production, we observed that the volumic production rate decreases slowly over this time scale for all promoters, and can often be fitted by a mixture of two exponential decay functions (Appendix section 4 and Appendix Fig. S10A).

To compare the decrease in production rate for different promoters during starvation, we measured the average volumic production rate in a given time window relative to the average rate during growth, e.g., RAPS_30–60_ refers to the relative average production in starvation during the period from 30 to 60 h in starvation. If the decay in production were driven primarily by the overall reduction of energy or of building blocks availability, then we would expect all promoters to have similar RAPS_30–60_ (estimated at ~10% for the promoter studied in Gefen et al. (2014)). In contrast, our data show that RAPS_30–60_ varies by more than two orders of magnitude (from 20% down to 0.1%) across our set of 22 promoters (Fig. 1E; Appendix Fig. S8B). The fact that RAPS_30–60_ is so variable across promoters underscores the profound changes that the gene regulatory network undergoes during starvation.

### Time-dependent protein concentration changes

While protein production rates define the current gene expression state of the cell, protein concentrations arguably define the physiological state of the cell, e.g., they dictate the rates at which different biochemical reactions occur within the cell. We thus also aimed to track what our transcriptional reporter observations imply for the protein concentrations of the corresponding genes.

**Fig. 2:**
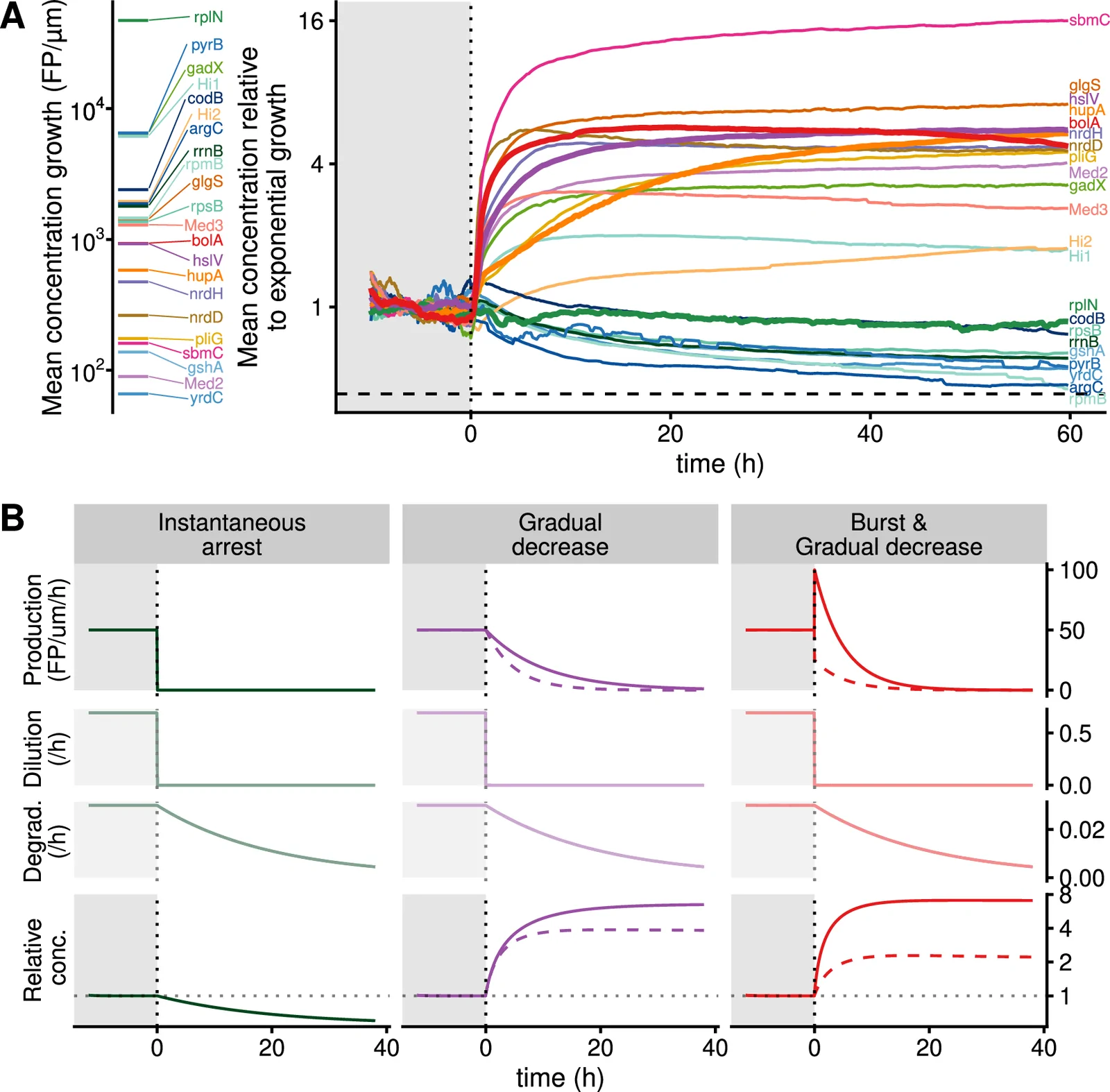
Dynamics of protein concentration during the switch from growth to starvation. (**A**) The protein concentrations corresponding to promoters used in this study span a broad range (ca. 100-fold) during exponential growth (left), and change in a promoter-dependent manner upon entry into starvation (right). Protein concentrations are measured in fluorescent protein per μm (FP/μm) and corrected for photobleaching (Appendix section 3). Time traces show averages across all cells (at least 20 to 400 per time point), scaled to the average concentration during exponential growth and shown on a logarithmic scale; promoters shown in Fig. 1D are indicated with thick lines. The horizontal dashed line shows the minimal concentration that would be reached, theoretically, in the case of instantaneous arrest of both production and growth upon entry into starvation (Appendix section 8). (**B**) Examples of protein concentration dynamics (bottom row) resulting from the combined time dynamics of protein production (top row), dilution (second row), and protein degradation (third row) for three archetypical scenarios of production dynamics (columns). In all scenarios, dilution drops to zero as growth stops immediately after the switch (Fig. 1B) and degradation rates decay exponentially during starvation as measured experimentally (Appendix Fig. S2). On the left, protein production halts immediately upon the switch; in the middle, it decays exponentially (at two different rates), while on the right, there is an initial sudden change in production, followed by exponential decay. The two extreme scenarios are representative of what is experimentally observed for ribosomal and RpoS-regulated promoters, respectively, while the middle one is an illustration of intermediate dynamics observed for promoters such as hslV or gadX. All parameters of the model are set after experimental observations (Appendix section 8). Note that the predicted concentration fold-changes are comparable to those observed experimentally in panel (**A**).

To obtain time traces of protein concentration for the genes corresponding to our reporters (Fig. 2A), we numerically integrated our estimates of the true time-dependent production rates of fluorescent proteins *q*(*t*), accounting also for the time-dependent protein degradation rate *γ*(*t*) (see Appendix section 3 for more details on the calculation). This approach relies on two main assumptions. First, we assume that the rate of degradation of native proteins during starvation is similar to that of the fluorescent proteins. Second, the translational efficiency and mRNA stability of the fluorescent reporters may differ from those of the corresponding native proteins. However, what matters for the predicted protein concentration fold-changes is how the translational efficiency and mRNA stability of a given gene change as the cells switch from exponential to stationary phase. As long as these *changes* are similar for the fluorescent reporters and native genes, the predicted fold-changes will still be accurate. As mentioned above, our calibration experiments on the degradation of fluorescent proteins show that degradation *rates* decrease exponentially, i.e., $\gamma (t)={\gamma }_{0}{e}^{-{\omega }_{\gamma }t}$, starting from initial values of degradation rate (*γ*_0_ ∈ [0.02−0.03] h^−1^, see Appendix Table S5). These rates are compatible with the overall protein degradation rate of native proteins at vanishingly slow growth (Calabrese et al, 2022), and the marked slowdown of degradation during starvation is also consistent with the fact that the vast majority of protein degradation in *E. coli* is performed by ATP-dependent proteases (Bittner et al, 2017; Maurizi, 1992) and that ATP levels decrease dramatically during starvation (Charbon et al, 2021; Lin and Jacobs-Wagner, 2022; Yeom and Groisman, 2021). Furthermore, changes in the levels of metabolites related to protein degradation upon entry into carbon starvation also indicate that degradation slows down during starvation (Lempp et al, 2019).

To validate that the fold-changes inferred from the transcriptional reporters reflect those of native proteins, we used quantitative proteomics to measure the temporal dynamics of native protein levels in batch cultures (Appendix section 7). Unfortunately, we found that the accuracy of the proteomics measurements was insufficient to meaningfully quantify the fold-changes of individual proteins (Appendix section 7 and Appendix Fig. S11). Indeed, publicly available proteomics datasets comparing growth and starvation show dramatic variability between replicates and datasets, confirming that it is impossible to obtain reliable quantitative estimates of the change in concentration of individual proteins from proteomics measurements (Appendix Fig. S11). However, although the proteomics measurements are not reproducible enough to quantify concentration fold-changes for individual proteins, we find that the trends are consistent between the proteomics measurements and the fold-changes inferred from our measurements in the Mother Machine with transcriptional reporters. In particular, for almost all proteins, the range of observed fold-changes in proteomics measurements overlaps with the fold-changes inferred from fluorescence measurements of the reporters in the Mother Machine (Appendix section 7 and Appendix Fig. S12). This consistency suggests that both the degradation rates and the changes in translation efficiency and mRNA decay cannot be too different between our fluorescent reporters and native proteins.

Thus, both the proteomics measurements and available knowledge in the literature are consistent with our assumption that the overall decrease of degradation due to reduced energy levels is the dominant effect for most genes, and that concentrations of fluorescent proteins used as transcriptional reporters are representative of the concentrations of the corresponding native proteins.

It is noteworthy that, among our set of promoters, there are almost none whose protein concentration remains stable across the switch, i.e., concentration either decreases due to degradation after a sharp production arrest, or increases more than twofold (Fig. 2A). Notably, synthetic promoters reporting constitutive gene expression (high expressors) are the closest to staying constant in concentration. For downregulated promoters, despite dilution stopping, the concentration can drop as low as ≈50% of their exponential levels due to protein degradation (Fig. 2A). In contrast, for promoters whose concentration increases, the relative change can be much larger, up to 12-fold across our set of 22 promoters. Strikingly, for those promoters, most of the changes seem to happen within the first 10 h of starvation, suggesting that the differences in production observed after prolonged starvation, i.e., RAPS_30–60_, are not playing an important role in the final concentrations reached at the end of the experiment.

Perhaps counterintuitively, even for promoters that do not exhibit a temporal burst in production but only undergo decreasing production, protein concentrations can increase substantially (i.e., more than twofold). Among the four examples of Fig. 1D, this is the case for both hslV and hupA (Fig. 2A right), although hupA takes far more time to reach the same fold-change. Mechanistically, this increase in protein concentration comes from the delay between the growth arrest (which is almost instantaneous) and the arrest of protein production (which is gradual and promoter-dependent). As a consequence, even for promoters for which protein production is reduced to very low levels during starvation, the protein concentration can nonetheless increase substantially during starvation.

### A simple mathematical model captures the diversity of observed concentration dynamics

Although the protein production time courses are highly diverse between promoters and differ in their details, it seems that most of these dynamics can be captured by two main variables: the amplitude of the initial burst of production and the delay between growth arrest and production arrest. We thus explored to what extent the diversity of protein concentration dynamics that we observe in our experiments can be captured by a simplified mathematical model (Appendix section 8; Appendix Table S3). In this model, all cells immediately arrest growth upon the switch to starvation (thereby ceasing dilution) and protein degradation decreases exponentially with time, i.e., with degradation rate $\gamma (t)={\gamma }_{0}{e}^{-{\omega }_{\gamma }t}$ as observed in our calibration experiments (Appendix Fig. S2).

For the protein production rate *q*(*t*) we assume that, upon entry into starvation, the production rate undergoes an instantaneous fold-change of size *b*, i.e., with *b* > 1 corresponding to a burst in production and *b* < 1 to an instantaneous reduction in production. After this, we assume that the protein production *q*(*t*) decreases exponentially at a rate *ω*_*q*_ that may differ between promoters.

Several simple scenarios of this model are illustrated in Fig. 2B. One extreme possibility is that production stops instantaneously upon entry into starvation, i.e., *b* = 0. On the other extreme end, production may jump up sharply after the entry into starvation (*b* > 1), but it is also possible that the production remains relatively unchanged (*b* ≈ 1). After the initial drop or burst, production then decays exponentially at some rate *ω*_*q*_ (note that two example values of *ω*_*q*_ are shown). As shown in Fig. 2B, the concentration dynamics resulting from the cessation of dilution, the time-dependent protein degradation, and these time-dependent production rates, recover the diversity of protein concentration dynamics that we observe experimentally. The *b* = 0 scenario corresponds to the dynamics observed for ribosomal promoters, and the *b* > 1 scenario captures the dynamics of promoters regulated by RpoS, such as bolA and Med3.

As shown in Appendix section 8, the fold-change in protein concentration between exponential growth and late stationary phase can be calculated analytically for this model and depends only on three effective ratios of rates. First, the total amount of decay is set by the ratio *r*_*γ*_ = *γ*_0_/*ω*_*γ*_. In particular, for a promoter that ceases production immediately upon entry into starvation, a fraction ${f}_{0}={e}^{-{r}_{\gamma }}$ remains late in the stationary phase, corresponding to about 44% of the initial concentration as illustrated in the left column of Fig. 2B.

Besides *r*_*γ*_, the fold-change in concentration increases with the ratio *r*_*q*_ = *bλ*/*γ*_0_, which corresponds to the ratio of the fold-change in production *b* and the fold-change in degradation/dilution *γ*_0_/*λ*. Finally, the fold-change also depends on the ratio *r*_*t*_ = *ω*_*q*_/*ω*_*γ*_ of the time scales at which production and degradation decay (Appendix Table S2).

Notably, introducing a delay in the arrest of production is enough to produce a large increase in concentration, e.g., there is a fourfold increase when the production decays with a characteristic time of 5 h, Fig. 2B, middle column. The early burst of production can further tune the concentration fold-change observed between growth and starvation, e.g., the sevenfold increase observed for bolA promoter (Fig. 2B, right column).

Because bolA is a known reporter of RpoS activity, we hypothesize that the increase in concentration we observe for this promoter, and by extension for others that display similar dynamics, may be primarily driven by RpoS activity. To test this hypothesis, we studied the concentration levels and fold-change for some of our promoters in a *Δ*rpoS background. Indeed, known targets of RpoS, such as bolA, are strongly downregulated in the mutant, both in the exponential and starvation phase (Appendix Fig. S13A). Moreover, with the exception of glgS, all promoters that were upregulated in starvation relative to the exponential phase in the wildtype show a decrease in upregulation in the *Δ*rpoS background (Appendix Fig. S13B).

However, while the drop in upregulation in the *Δ*rpoS background is dramatic for some RpoS targets such as hslV, gadX, and the synthetic promoters Med2 and Med3, other RpoS targets such as hupA and bolA show only a small decrease in upregulation. This suggests that other regulatory mechanisms besides RpoS contribute to the gene expression changes during starvation.

### Gene expression dynamics under gradual entry into the stationary phase is similar to those under a hard switch

Our results so far concern the gene expression response upon a sudden “hard switch” from exponential growth to starvation conditions without a carbon source. Such an almost instantaneous depletion of nutrients might be rare in the natural environments of *E. coli*, and it is conceivable that the gene expression response might be quite different when the depletion of the nutrients is slower, perhaps allowing cells to sense the loss of nutrients.

**Fig. 3:**
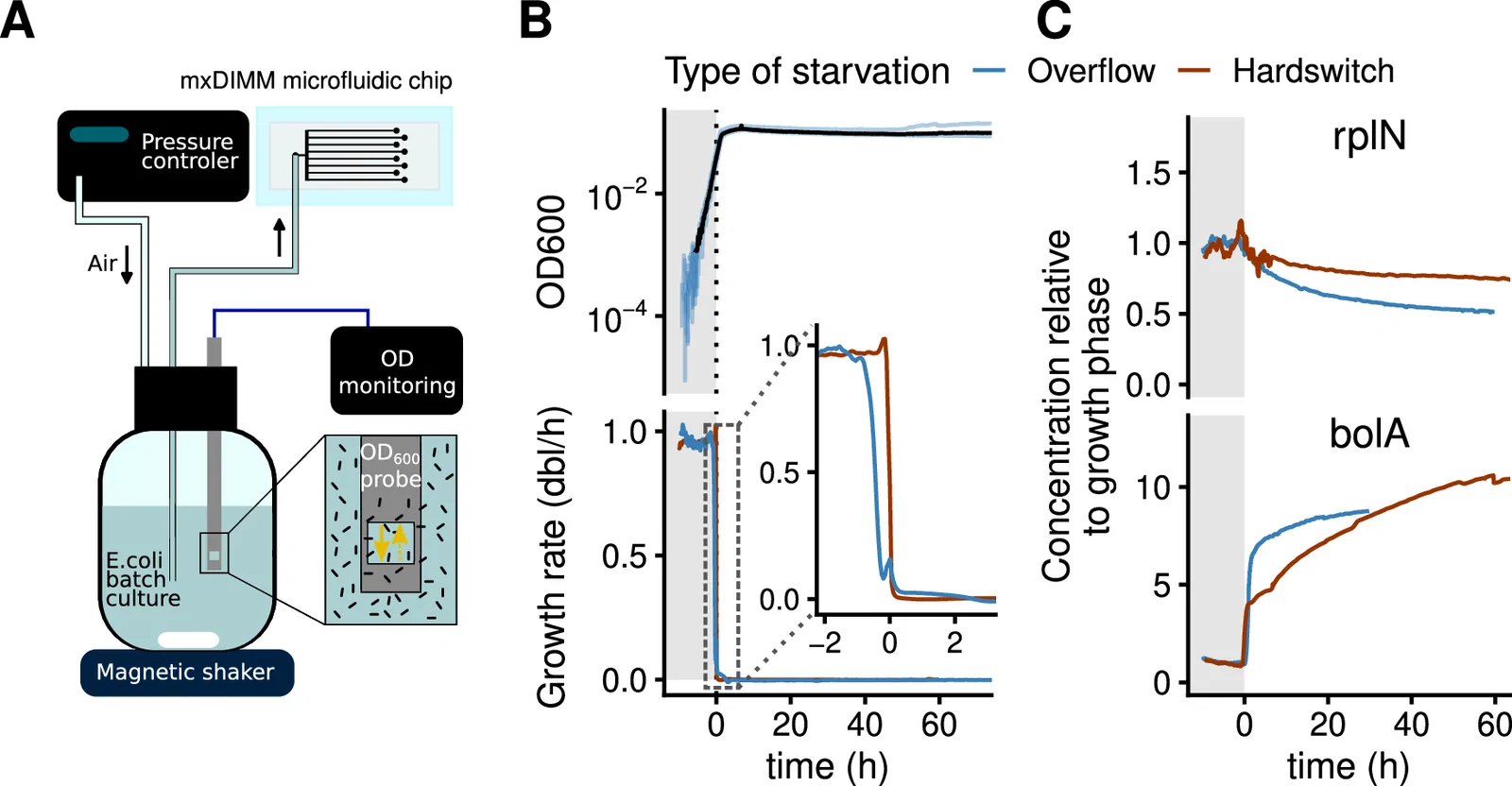
Temporal dynamics of gene expression is similar during gradual entry into the stationary phase. (**A**) A batch culture of *E. coli* growing in M9+0.02% glucose was connected to a microfluidic chip to arrest the growth of cells trapped in the channels of the multiplexed DIMM. (**B**) The OD_600_ in the flask was followed along with the average growth rate of cells in the chip to verify that the growth arrest was synchronized between flask and chip. Time 0 was defined as the moment where average growth rate becomes lower than 0.055 dbl/h. Top: individual blue lines correspond to three replicates, while the black line is the mean of those replicates. Bottom: average instantaneous growth rate across all cells, along time, measured in the two experimental setups, indicated with colors. The inset is a zoom on a 5 h time window spanning the entry into starvation. At least 3500 cells are present per time point for the hard switch, against 110 for the overflow setup. The growth arrest is more progressive in the overflow setup (blue line, duration ≈45 min; see Appendix Fig. S14A, B) than in the hard-switch setup (red line, duration ≈5 min; see Appendix Fig. S4B). (**C**) For two example promoters, the temporal dynamics and magnitude of the relative concentration (mean of at least 60 cells per time point), computed as in Appendix section 3, is similar between a hard switch starvation and a gradual entry into the stationary phase (see also Appendix Figs. S14B and S16). Note that in those experiments, rplN and bolA reporters are chromosomally integrated, and bolA controls the expression of mCherry2-L and not GFPmut2 (see the Reagents and tools table and Appendix Table S6 for more details on the strains used in the different experiments).

To address this question, we did another series of microfluidic experiments where a culture growing in M9 + 0.02% glucose is flown through a mother machine microfluidic chip, so as to expose bacteria in the chip to the same physico-chemical conditions as in the culture flask, where nutrients are depleted due to the uptake by bacteria (Fig. 3A), i.e., using the same approach as in Gefen et al. (2014) and Bakshi et al. (2021). In order to ensure that glucose is exhausted before other nutrients, we used a lower glucose concentration than in our hard switch experiments (Chubukov and Sauer, 2014).

We observed that under this gradual entry into the stationary phase, the instantaneous growth rates decrease over a longer period than for the hard switch. That is, while the growth rate dropped from its maximum value to zero in ca. 5 min in experiments with instantaneous switching, this transition took ca. 45 min in these culture overflow experiments, which almost certainly indicates that nutrients are exhausted more gradually (Appendix Fig. S14A,B). It is interesting to contrast this time scale with the sharp angle of the growth curve observed at the population level in this media (Fig. 3B). For comparison of the gene expression dynamics between the two transitions, we define the *t* = 0 start of starvation for the overflow experiments as the time when the instantaneous growth rate (averaged over all individual cells) has decreased by 95% (which corresponds to 0.055 dbl/h; Fig. 3B; Appendix Fig. S14A,B).

Although the growth variability across cells increases during the transition to growth arrest (Appendix Fig. S14B), which does not occur when cells are switched abruptly (Appendix Fig. S4B), all cells eventually go into growth arrest after about an hour and there is no subpopulation of cells which continues to grow for an extended period of time, in spite of the presumed presence of by-products of glucose metabolism which might in principle be used as nutrients (Lim et al, 2025; Orr et al, 2019). As a side note, because there is variation in growth rates across cells, there will also be variability in the number of cells exiting across the channels of the microfluidic device, and this could, in principle, bias observations relative to batch culture (where no cells are lost). However, as shown in Appendix Fig. S15, this effect is so tiny that it can safely be ignored in our analyses.

While the entry into growth arrest is clearly more gradual, the dynamics of protein production rates (Appendix Fig. S14C) as well as the inferred resulting dynamics of protein concentrations (Fig. 3C; Appendix Fig. S16) are very similar to those measured in experiments with a hard switch. This suggests that the gene expression response upon loss of nutrients and cessation of growth is fairly independent of the speed of the switch and the precise amounts and types of residual carbon sources, and may apply to a wide range of environmental transitions from growth to starvation.

### Gene expression changes during early starvation are highly consistent across single cells

**Fig. 4:**
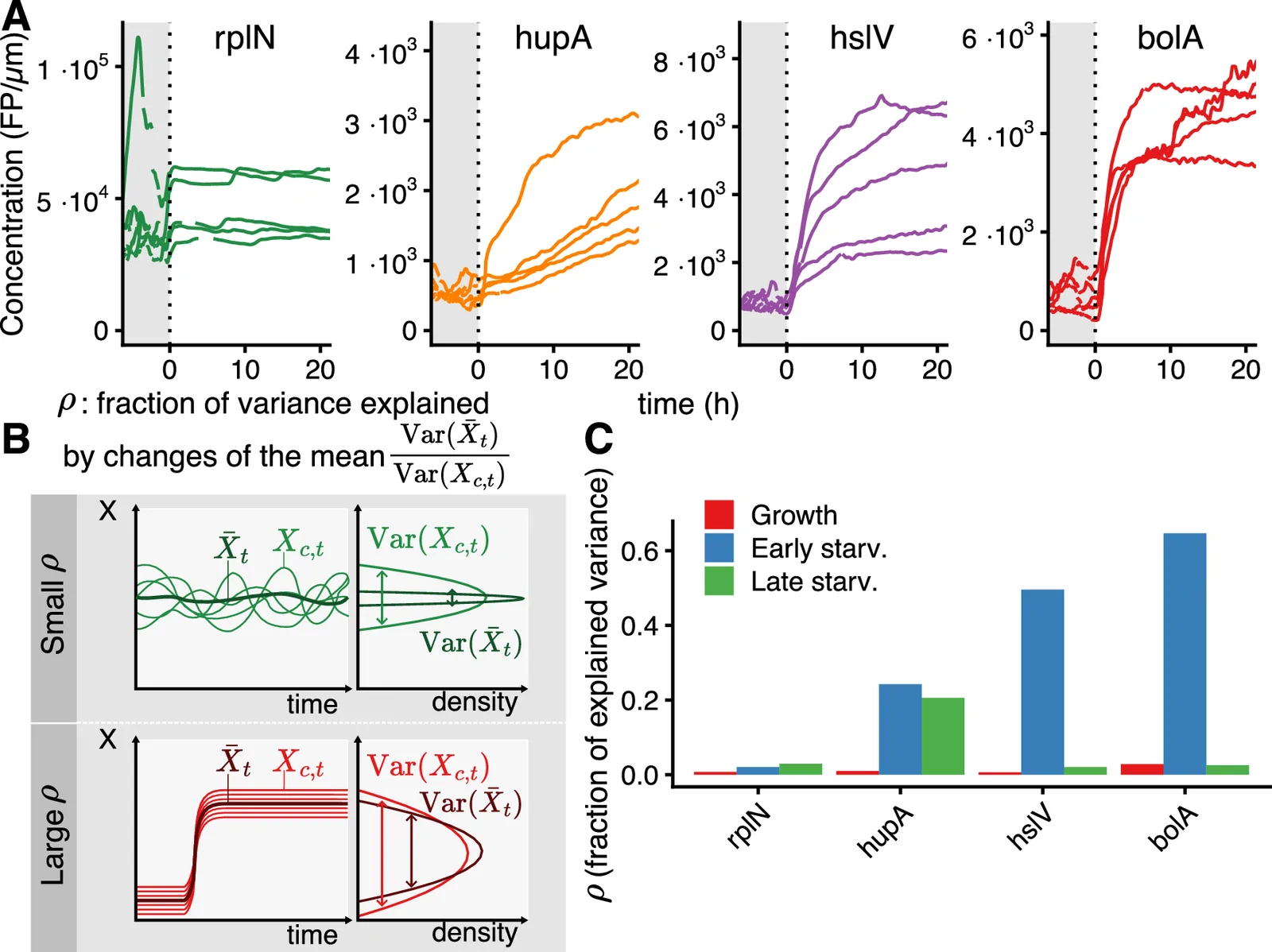
Protein concentration dynamics at the onset of starvation are highly consistent across single cells. (**A**) Each panel shows the protein concentration dynamics for a random selection of single cells (lines) for one representative promoter (indicated at the top), illustrating that the dynamics is very similar across single cells. (**B**) To quantify the consistency of the protein concentration changes across single cells, we calculate *ρ*, the ratio of the variance in the population average protein concentration across time and the variance of all individual protein concentration measurements. Two limit cases illustrate the meaning of *ρ*. When individual cells fluctuate around a constant value, the variance of the population average across time is small compared to total variance (*ρ* ≈ 0; top row). In contrast, when all cells follow the same dynamics starting from slightly different values, the total variance is close to the population average variance (*ρ* ≈ 1; bottom row). (**C**) Values of *ρ* for the same four representative promoters during exponential growth (red), the first 10 h of starvation (blue), and late starvation (green) show that *ρ* is systematically higher during early starvation; corresponding results for all promoters are shown in Appendix Fig. S17.

Like the dynamics of volumic protein production (Fig. 1C), the dynamics of protein concentration is also highly consistent across single cells (Fig. 4A; Appendix Fig. S17A). To characterize the consistency in the response to starvation at the single-cell level, we quantify for each reporter how much of the total variance in protein concentration across cells and time is explained by the time-dependent population average. That is, we measure the ratio *ρ* of the variance in the population-average protein concentration across time and the total variance of all protein concentration observations across all cells $\rho ={{\rm{Var}}}({\bar{X}}_{t})/{{\rm{Var}}}({X}_{c,t})$, where *X*_*c*,*t*_ is the protein concentration in individual cell *c* at time point *t* and ${\bar{X}}_{t}$ is the population average concentration at time *t*. Note that when the concentration fluctuates around a fixed value, *ρ* is close to zero, whereas in the limit that all cells in the population exhibit the same time-dependent changes, *ρ* will approach its maximum value of 1 (Fig. 4B). Especially for promoters for which the protein concentration increases upon entry into starvation, *ρ* is much larger during the first 10 h of starvation than during exponential growth or later in starvation (Fig. 4C; Appendix Fig. S17B). For bursting promoters (e.g., hslV and bolA), more than 50% of the observed variation across all cells during early starvation is captured by the population average behavior, while *ρ* is smaller for promoters only displaying a delayed arrest of production (e.g., 25% for hupA).

To put these numbers into context, we compared the consistency of the response to loss of carbon source with the consistency in the response to another stress. Our lab recently quantified the single-cell responses of target promoters of the LexA regulon upon mild DNA damage induced by low doses (0 to 2 ng/mL) of ciprofloxacin (Galbusera et al, 2025). Although the LexA target promoters are induced to a similar degree at the population level (up to 2.5-fold upregulation), there is almost no consistency at all across single cells in this response. In particular, values of *ρ* are below 0.05 both before and after induction (Appendix Fig. S18).

The stark contrast with the high consistency of the gene expression response upon entry into starvation suggests that the dynamic properties of the starvation response (i.e., the amplitudes of the burst and time scale of the arrest in volumic production for a given promoter) are tightly regulated.

### Protein concentrations late in starvation are set by production during early starvation

**Fig. 5:**
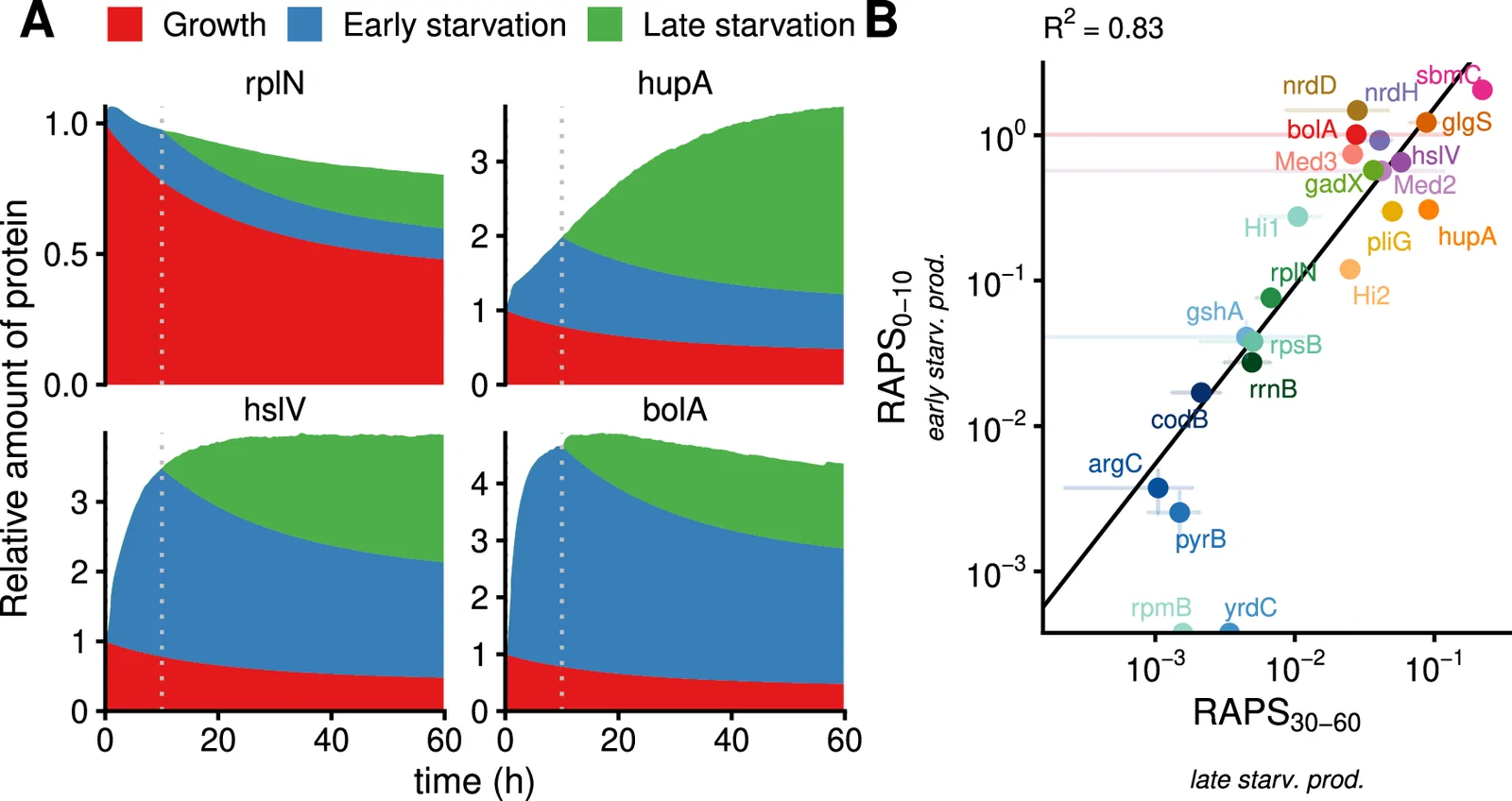
Protein concentrations late in starvation are set by protein production early in starvation. (A) Each panel shows the relative amounts of proteins present at a given time in starvation that derive from production during exponential growth (red), early starvation (blue), and late starvation (green) for an example promoter (indicated at the top). The amount of protein derived from each time period is calculated by integrating the volumic protein production rate forward in time and accounting for protein degradation (Appendix section 3); “early starvation" (before the vertical dotted lines) corresponds to the first 10 h of starvation, while “late starvation” corresponds to 10–60 h (after the vertical dotted lines). The quantities are normalized by the total protein amount at the start of starvation. (**B**) The level of protein production during late starvation, quantified by RAPS_30–60_, is correlated with the production during early starvation as quantified by RAPS_0–10_ (Pearson correlation *R*^2^ = 0.83; error bars correspond to RAPS ± s.d., calculated by Gaussian error propagation from the relevant average volumic productions; 10^4^ to 10^5^ observations per point, on 60 to 470 cells in starvation and 630 to 6000 cells in exponential growth). Note that two promoters with negative RAPS_0–10_ due to negligible production, are set to 0 and excluded from the fit (rpmB and yrdC). As discussed in the Methods and Appendix Fig. S5A, B, at late starvation, there is evidence of the effects of photoxicity caused by our fluorescence illumination protocol, which might also affect gene expression. This makes the observed strong correlation between early and late production especially notable.

An important consequence of the fact that volumic production rates drop to low levels in late starvation is that protein concentrations deep in starvation will strongly depend on gene expression early in starvation, and perhaps even on production during exponential growth. To quantify the relative contributions from gene expression in different periods to the protein concentrations late in starvation, we measured, for each time point during growth arrest, the amounts of proteins that derive from production in the exponential growth phase, during the first 10 h of starvation, and after the first 10 h of starvation. Figure 5A shows these amounts, relative to the total amount of protein at the start of starvation, as a function of time in starvation for the same four example promoters displayed in previous figures. We see that for strongly downregulated promoters, such as rplN, the protein levels late in starvation are dominated by proteins produced during exponential growth. For promoters with delayed production arrest (e.g., hslV) or with a burst (e.g., bolA), the majority of proteins derive from production that happened during the first 10 h of starvation (blue segments in Fig. 5A). For promoters with significant sustained expression (e.g., hupA), the proteins produced later in starvation eventually come to constitute the majority (green segments in Fig. 5A), although this is relatively rare (see Appendix Fig. S19A for results on all promoters). These observations hold for all promoters displaying similar temporal dynamics of volumic production, and emerge from highly consistent behaviors across individual cells (Appendix Fig. S19B).

Interestingly, we find that the ratio of the expression early in starvation (RAPS_0–10_) and of the expression late in starvation (RAPS_30–60_) are strongly correlated (Pearson *R*^2^ = 0.83; Fig. 5B). That is, promoters that display high protein production early in starvation also tend to have sustained expression late in starvation. This suggests that the mechanism controlling gene expression during the entry into starvation might be involved in its sustainment late in starvation as well. As an aside, we compared mortality after 60 h of starvation for experiments with and without illumination and found that, while without illumination the large majority of cells survives, with constant illumination less than half of the cells survive (Appendix Fig. S5A). This raises the question of whether phototoxicity may also affect gene expression. Although we cannot exclude some effects on gene expression, the fact that there is virtually no mortality after 25 h with illumination, that most protein concentration changes occur during the first 10 h, and that protein production in early and late starvation are well-correlated, suggests that the effect of phototoxicity on gene expression cannot be too severe.

The correlation between production in early and late starvation also implies that the changes in protein concentrations happening during the first 10 h tend to persist later on. As a consequence, the cells maintain an approximately fixed phenotypic state for days.

### The gene expression response early in the stationary phase is crucial for stress tolerance later in the stationary phase

We have shown that protein production rates are dramatically remodeled during the first 10 h of the stationary phase, causing major changes in protein concentrations, and that this new gene expression state is then rather stably maintained over the next two days of starvation. It is tempting to speculate that the functional role of this remodeling of gene expression early in starvation is to phenotypically prepare the cells for survival and stresses that may occur later during starvation. Indeed, as protein production rates eventually become very low, cells are likely unable to respond to environmental changes late in starvation.

To test this hypothesis, we initially aimed to investigate the role of gene expression during the first 5 h of starvation by comparing cell survival under normal conditions versus conditions in which protein translation is inhibited during the first 5 h by a low dose of chloramphenicol. However, we found that, quite interestingly, the addition of chloramphenicol during the first 5 h of starvation did not prevent the burst of expression observed in RpoS-regulated genes, but simply postponed it until the end of the chloramphenicol treatment (Appendix Fig. S20).

**Fig. 6:**
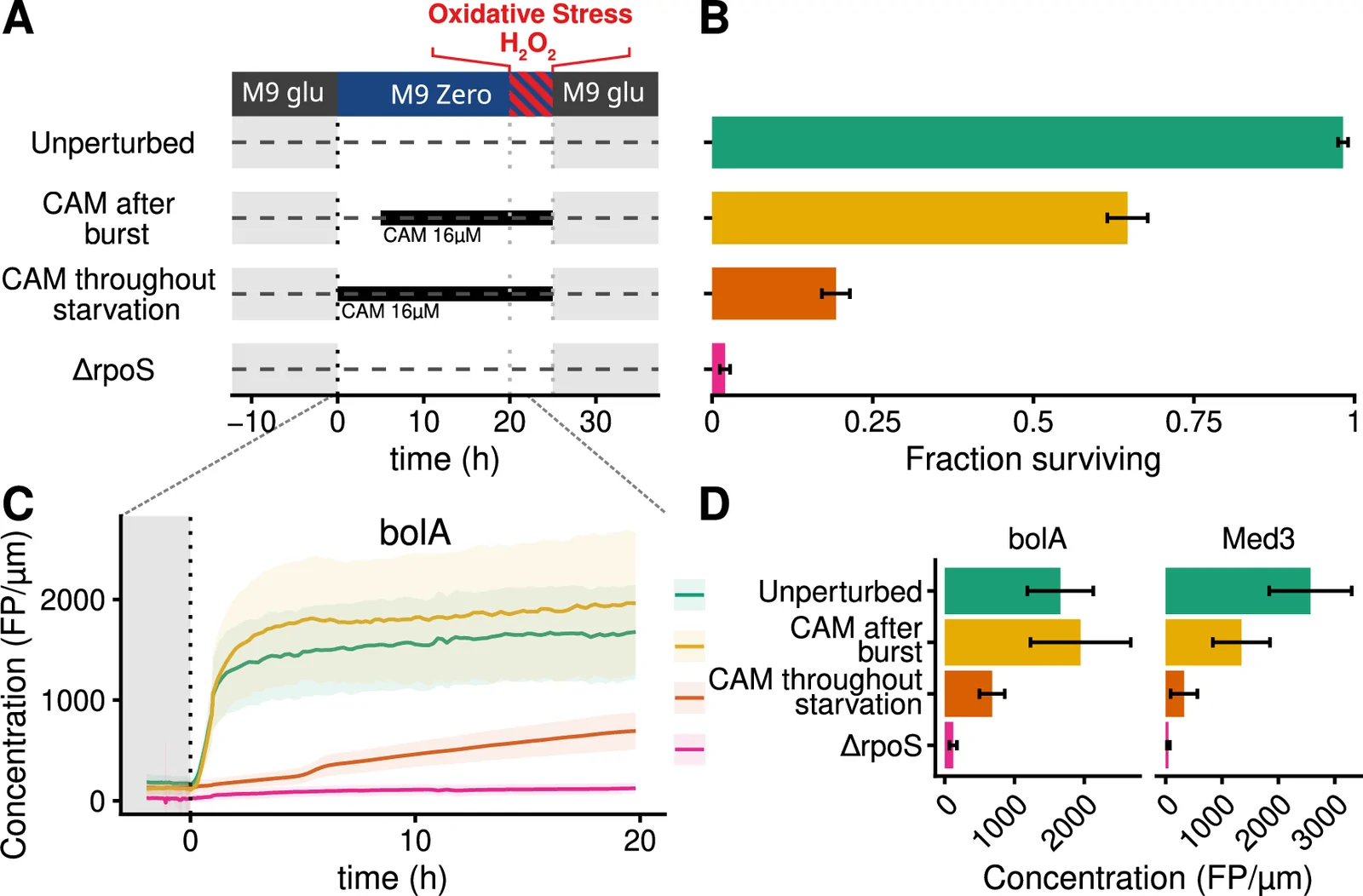
The remodeling of gene expression at the onset of starvation is essential for survival of oxidative stress later in starvation. (**A**) *E. coli* cells inoculated in the DIMM microfluidic chip are switched from M9 glucose to starvation in M9 zero and, after 20 h, submitted to oxidative stress during 5 h (0.65–0.75 mM ${{{{\rm{H}}}}_{{{\rm{2O}}}}}_{{{\rm{2}}}}$), before being switched back to fresh media in order to assess survival. In addition to this default treatment (“Unperturbed”), gene expression can be inhibited using chloramphenicol, either starting after 5 h of starvation (“CAM after burst”), or during the entire starvation time (“CAM throughout starvation”). To assess the role of the RpoS sigma factor in the gene expression response, we also subjected a *Δ*rpoS mutant to the same treatment as the “Unperturbed” case. (**B**) Stress survival under the different treatments is quantified as the fraction of cells regrowing and dividing within the time of observation in fresh media (20 h; error bars correspond to the fraction ±s.d. over 230 to 330 observed cells). (**C**) Concentration profiles of the bolA fluorescent reporter (as described in Fig. 2) up to the point of the oxidative stress challenge for the various treatments (in colors). The line and ribbon correspond to the mean ± s.d. of 250 to 340 cells per time point. (**D**) Concentration levels of RpoS-regulated reporters (bolA and Med3) at the moment of the oxidative stress challenge, for the different treatments (colors). Means and standard deviations are based on data from two independent experiments and two similar H_2_O_2_ concentrations. Results of individual replicates are shown in Appendix Fig. S21. Note that in these experiments, Med3 and bolA reporters are chromosomally integrated, and control the expression of mVenus and mCherry2-L, respectively and not GFPmut2 (see the Reagents and tools table and Appendix Table S6 for more details on the strains used in the different experiments).

We thus decided to instead compare the effect of inhibiting protein synthesis with 16 μM chloramphenicol during the entire starvation period to the effect of the same treatment starting 5 h after the entry into starvation (Fig. 6A). In the latter, there is only protein production during the first 5 h, while the former constitutes a control where protein production is strongly inhibited during the entire starvation period. However, we found that irrespective of translation inhibition, essentially all cells survive 1 day of starvation in our setup (Appendix Fig. S21C). Instead of testing survival to longer periods of starvation, we decided to quantify the importance of the gene expression remodeling at the onset of starvation for the stress tolerance of the cells. In particular, we compared the tolerance to a strong oxidative stress (0.65 or 0.75 mM hydrogen peroxide H_2_O_2_) from 20 to 25 h of starvation between several treatments: normal starvation, translation inhibition after 5 h in starvation, and translation inhibition during the entire starvation period (Fig. 6A).

We quantified stress tolerance by the fraction of cells surviving the oxidative stress (defined as regrowing within 20 h after the termination of the stress and switch to fresh media). We find that, without translation inhibition, there is almost no mortality (98.2% survival), which indicates that most cells can handle this level of oxidative stress in the absence of additional perturbations (Fig. 6B). In contrast, inhibiting gene expression with chloramphenicol during starvation increased mortality. Most importantly, the difference between the treatment with translation inhibition after 5 h in starvation and inhibiting translation from the start demonstrates that the initial remodeling of gene expression strongly decreases the sensitivity to oxidative stress (65 vs 19% survival; Fig. 6B). This confirms that the remodeling of gene expression upon entry into starvation prepares the cell for stresses that may occur later in starvation.

Using the transcriptional reporters described previously, we can also assess the effects of translation inhibition on the concentration of RpoS-regulated promoters (i.e., bolA and Med3; Fig. 6C; Appendix Fig. S21A,D). As expected, starting the inhibition after 5 h does not significantly affect the levels of these reporters during starvation. In contrast, upon chloramphenicol treatment during the entire starvation, their concentration is reduced more than twofold after 20 h. Notably, the concentrations at the time of the oxidative stress under the different treatments have the same order as the fraction surviving (Fig. 6D), suggesting that the induction of RpoS- regulated genes might underlie a large part of the protective effect.

To test this further, we submitted a *Δ*rpoS strain to the same assay of resistance to oxidative stress during starvation (without translation inhibition). We find that rpoS-regulated reporters were not expressed anymore, not only during starvation but also during growth (Fig. 6C,D), strongly suggesting that their expression during growth in the wildtype reflects RpoS activity during growth. Remarkably, the survival after oxidative stress dropped almost to zero and was even lower than in the wild-type strain with translation inhibition during the whole starvation period, confirming the importance of this global regulator to survive oxidative stress during starvation. The fact that deleting rpoS had a stronger impact on the sensitivity to oxidative stress than inhibiting the gene expression globally during the whole starvation could, in part, be due to incomplete inhibition by chloramphenicol at this concentration, but we suspect that the rpoS activity during growth, causing some expression of rpoS-regulated genes before starvation, also contributes to this effect (Appendix Fig. S13A). Overall, these experiments show that the remodeling of gene expression upon entry into starvation is crucial for resistance to stresses that may occur later in starvation.

## Discussion

Although bacteria can rapidly expand their numbers exponentially when nutrients are available, nutrients always eventually run out, forcing the bacterial cells to withstand periods of starvation, which in the wild are likely frequent and extended. Yet, very little is known about how bacteria adapt their phenotypes when faced with starvation. To address this lack of knowledge, we have here tracked, with high quantitative precision and time resolution, the dynamics of growth and gene expression at the single-cell level across a set of representative genes in *E. coli* when cells undergo carbon starvation.

We first discuss how our findings fit in the context of previous studies. First, our main observations are that, after cells go into growth arrest upon loss of carbon source, there are major changes in the rates at which proteins of different genes are being produced, causing a major remodeling of the proteomes of the cells in the first 5–10 h. Notably, although the overall rate of protein production is much reduced, and certain classes of normally highly expressed genes, such as ribosomal proteins, almost completely stop being produced, other genes, such as those regulated by the sigma factor RpoS, show a transient increase in their production rate. Importantly, we found that both protein production and degradation decay to very low levels after 10 h, and that these changes in protein concentrations are subsequently stable for several days. Importantly, we also found that these changes are crucial for stress tolerance during starvation. These observations explain a previous report that gene expression during the first 10 h of starvation is more crucial for cell survival than expression at later times (Reeve et al, 1984a).

Second, it was known that, upon entry into the stationary phase, the average cell size decreases due to reductive division, but this process was believed to take place as cells slow down prior to growth arrest (Bakshi et al, 2021; Shimaya et al, 2021). Our results show, however, that all cells immediately go into growth-arrest upon loss of the carbon source, and reductive divisions occur in this growth-arrested state (Appendix Fig. S4). In addition, we found that reductive divisions predominantly occur among cells that are further advanced in their cell cycle, confirming a previous hypothesis (Nyström, 2004).

Third, we observed that the vast majority of cells survive during the 60 h of starvation in our experiment. This may seem contradictory with recent articles on *E. coli* starvation, in which the survival was reported to decrease exponentially from the onset of starvation (leading to ca. 50% mortality after 2 days; Phaiboun et al, (2015); Schink et al, (2019)). However, it has been observed that survival dynamics depend on strains and conditions (Bergkessel and Delavaine, 2021). In a previous study using the same strain (*E. coli* MG1655) and the same conditions (M9 minimal media), it has been observed that survival curves measured in bulk only started to decay exponentially after several days (Dukan and Nyström, 1999). Similarly, results from microfluidics experiments also report more than 90% survival during the first 60 h of starvation (Yang et al, 2023). Our observations are thus in agreement with previous reports. However, the differences in survival between strains and conditions call for a more comprehensive study of the strain- and condition-dependence of *E. coli* survival under starvation.

Last, with regard to gene expression during starvation, some previous studies have suggested that, once bacteria are growth-arrested and starving, they can no longer substantially alter their phenotype (Biselli et al, 2020). Similarly, in another study, it was proposed that adaptation to starvation only occurs when cells progressively deplete nutrients and sense the impending starvation as their growth slows down (Phaiboun et al, 2015). In contrast, Gefen et al. reported that bacteria can sustain expression, i.e., production of new proteins, over an extended period of time (≈2 days) in starvation (Gefen et al, 2014), and reported an approximately constant sustained expression for a particular gene, suggesting that phenotypes can keep adapting deep into the stationary phase.

Here we have shown that, contrary to the hypothesis that bacteria need to adapt their expression state before growth stops, marked gene expression changes take place during the first 10 h of starvation. Moreover, the absence of dilution causes the increase of protein concentration for genes that do not immediately halt production (Fig. 2B). Also, in contrast to the hypothesis that cells need to sense impending starvation for their adaptive response, we found that the temporal dynamics as well as the amplitude of the adaptation were very similar when cells entered starvation gradually over a 45 min period as in a batch culture (Fig. 3), further supporting that bacteria adapt gene expression in the first hours after growth arrest. Moreover, even if protein production is inhibited during the first 5 h of starvation, essentially identical dynamics of protein production are subsequently observed after the inhibition is released (Appendix Fig. S20), strongly suggesting that this protein production is driven by the use of reserves or recycling of resources inside the cell.

In contrast to Gefen et al. (2014), we found that protein production systematically decreases with time and that the main changes in proteome composition occur during the first 10 h of starvation. Moreover, we show that the level of gene expression during starvation, as well as its temporal dynamics, are highly promoter-dependent, with some genes showing a transient increase in expression, and different genes exhibiting different rates of decay in protein production with time (Fig. 2B). In particular, in our sample of ≈20 promoters, those levels of protein production late in starvation (relative to that during exponential growth) span more than two orders of magnitude (Fig. 1E). The level of about 10% reported by Gefen et al. is within this range, at the upper end.

The sustained gene expression activity that we observe raises the question: how can cells maintain this energy-demanding process in the absence of a carbon source? It is known that, during starvation in *E. coli*, intracellular ATP levels drop rapidly, with 70–90% depletion occurring within minutes. The level then decreases more slowly afterward, remaining at around 10% of the exponential growth levels for an extended period of time (Jensen et al, 1995; Lempp et al, 2019; Mancini and Pilizota, 2023; Yeom and Groisman, 2021). We believe that the sustained gene expression is likely fueled by different energy sources on different time scales. First, while a large fraction of protein production is allocated to production of ribosomal proteins during exponential growth (Bremer and Dennis, 2008; Mori et al, 2021), we observe that ribosomal protein production stops immediately upon entering starvation (Fig. 1C), so that whatever production remains shifts to production of other proteins. This may cause the protein production of many genes to effectively increase, even if the total production is much lower. Second, intracellular energy storage, such as glycogen, might transiently fuel gene expression activity very early in starvation, although it has been reported to be depleted within minutes at the onset of starvation (Sekar et al, 2020).

It thus seems likely that the gene expression activity during prolonged starvation must be sustained mainly by endogenous metabolism (Dawes and Ribbons, 1964), i.e., by the hydrolysis of abundant cellular constituents such as proteins and RNAs, which release metabolites that can be used for producing ATP and precursors of gene expression. Our measurements of the degradation of several fluorescent proteins during the first days of starvation (Appendix Fig. S2) show that their rate of protein degradation itself decays exponentially, and that on the order of half of the fluorescent proteins present at the onset of starvation are eventually degraded (Fig. 2). Assuming that other cytoplasmic proteins undergo similar degradation, ~40% of the proteome will be degraded within the first day of starvation, which constitutes significant amounts of energy and metabolites for fueling gene expression. In addition, early experiments with mutants lacking up to five different proteases indicated that their activity was important for survival during sustained starvation (Reeve et al, 1984b), which also supports the importance of proteolysis for releasing resources during starvation. In order to further investigate the hypothesis that endogenous metabolism fuels gene expression during starvation, it will be important to identify the proteases and nucleases active during this period in order to measure gene expression when their levels are perturbed.

Our use of microfluidic devices (Kaiser et al, 2018), first of all, allowed us to make controlled changes in the environment and track growth and gene expression in constant starvation conditions. This is in contrast to batch cultures, where, during the stationary phase, bacteria continue to modify the composition of the media over time, which may have important confounding effects on the observed gene expression dynamics. Crucially, our microfluidics setup also allowed us to quantitatively track gene expression at the single-cell level. These single-cell measurements strikingly revealed that, although the common belief is that phenotypic variability is higher during stationary phase than during growth (Bergkessel et al, 2016; Guido et al, 2007), the response to starvation that we have discovered is remarkably homogeneous across cells (Fig. 4). This homogeneity in single-cell behavior at the onset of starvation suggests the existence of a tightly controlled regulatory program that allocates available resources to reshape the cell phenotype before energy runs out completely.

By what precise regulatory mechanisms bacteria commit to this new expression program remains to be elucidated, but our study already provides several relevant insights. Firstly, when gene expression is inhibited with chloramphenicol during the initial 5 h of starvation, the response simply appears to occur 5 h later, and protein concentrations of induced genes still increase to levels similar to those achieved without perturbation (Appendix Fig. S20). This implies that the decrease in production rate during the first 10 h of starvation is likely the result of energy depletion by expression itself, rather than a specific regulatory mechanism at the transcriptional or translational level. Importantly, this also indicates that the total gene expression that is achieved during this period is primarily set by the amount of available intracellular resources. In order to gain more insight into how the total gene expression during starvation is set by the state of the cell when it enters growth arrest, it will likely be very informative to alter the metabolic capacity of the cells at the moment they enter growth arrest by varying the prestarvation conditions.

The fact that different promoters show such variable time-dependent responses in protein production raises the question as to what regulatory mechanisms implement these variable responses. For example, immediately upon entry into starvation, ribosomal protein promoters are almost completely shut down. Interestingly, essentially all other promoters, including those that show a transient burst in production during the first hours of starvation, appear to also exhibit an initial very short drop in production. This suggests the existence of a general mechanism that briefly halts protein production upon entry into the stationary phase. Further, our results with the knockout of rpoS (Fig. 6) show that, as expected, this sigma factor plays an important role in the expression of transiently upregulated genes. However, we suspect that other major regulators, such as the major regulator of catabolism CRP, might also contribute to this transient upregulation.

Especially intriguing is the fact that, for promoters whose expression is sustained during prolonged starvation, the decay of the volumic production *q* happens at different rates for different promoters (Appendix Fig. S10A). This indicates that the approximately exponential decay of production is not just controlled by an overall loss of expression capacity, because this would imply that the production decays at the same rate for all promoters. However, we must keep in mind that the volumic production for a given promoter is the product of an overall rate of protein production and its mRNA concentration. The different rates of decay of production for different promoters thus suggest that mRNA levels continue to shift over two days of starvation. Further insight into the underlying mechanisms could perhaps be gained using dual reporters that report on the rate of production from two copies of the same promoter, or the production from two different promoters with different rates of decay. This would, for example, show whether the rates of production decay are also different within the same cell.

Perhaps the most intriguing question is what the precise functional role is of the phenotypic changes happening in the first 5–10 h of starvation. Our experiments show that the cells easily survive 25 h of starvation in our setup, even when their gene expression is inhibited throughout the whole starvation period (Appendix Fig. S21C). Thus, the gene expression response is not necessary for survival on the time scale of 1 day, but it might well be important for survival on longer time scales. Indeed, a previous study has already reported that the first 10 h of expression during starvation are most important for survival on a longer time scale (Reeve et al, 1984a). Our results further show that the gene expression occurring during the first 5 h of starvation is crucial for *E. coli* bacteria to survive a stress coming 15 h later. Thus, the gene expression response upon entry into starvation is crucial both for ensuring survival on longer time scales, and for protecting cells against stress.

In general, it might be difficult to disentangle which aspects of the expression response upon starvation are mainly to support survival and which are mainly to protect against possible stress. To learn more about how natural selection has shaped the functional role of the expression response upon entry into starvation, it would be interesting to systematically compare the response across different strains of *E. coli* as well as the differences in survival and tolerance to various types of stress across these strains.

## Methods

### Reagents and tools table

**Table:** 

Reagent/resource	Reference or source	Identifier or catalog number
**Experimental models**		
*E. coli* K12 MG1655	Migula (1895); Castellani and Chalmers 1919	DSM 18039; CGSC 6300
**Strains**		
MG1655 attBHK022::(Med2p-mVenus,Med2p-mCerulean) attB186::(bolAp-mCherry2-L)	This study; promoters from Wolf et al, 2015; Zaslaver et al, 2006	Med2 bolA
MG1655 attBHK022::(Med2p-mVenus,Med2p-mCerulean) attB186::(bolAp-mCherry2-L) ∆rpoS	This study; promoters from Wolf et al, 2015; Zaslaver et al, 2006	Med2 bolA ∆rpoS
MG1655 attBHK022::(Med3p-GFPmut2)	Urchueguia et al, 2021	Med3
MG1655/pUA66-Med3p-GFPmut2	Wolf et al, 2015	Med3 Pl.
MG1655 attBHK022::(Med3p-mVenus,Med3p-mCerulean) attB186::(bolAp-mCherry2-L)	This study; promoters from Wolf et al, 2015; Zaslaver et al, 2006	Med3 bolA
MG1655 attBHK022::(Med3p-mVenus,Med3p-mCerulean) attB186::(bolAp-mCherry2-L) ∆rpoS	This study; promoters from Wolf et al, 2015; Zaslaver et al, 2006	Med3 bolA ∆rpoS
MG1655 attBHK022::(pHi1-GFPmut2)	Urchueguia et al, 2021	Hi1
MG1655 attBHK022::(pHi1-GFPmut2) ∆rpoS	Urchueguia et al, 2021	Hi1 ∆rpoS
MG1655 attBHK022::(Hi2-mVenus,Hi2-mCerulean) attB186::(bolAp-mCherry2-L)	This study; promoters from Wolf et al, 2015; Zaslaver et al, 2006	Hi2 bolA
MG1655 attBHK022::(gshAp-GFPmut2)	Urchueguia et al, 2021	gshA
MG1655 attBHK022::(yrdCp-GFPmut2)	This study; promoter from Zaslaver et al, 2006	yrdC
MG1655 attBHK022::(rplNp-GFPmut2)	This study; promoter from Zaslaver et al, 2006	rplN
MG1655 attBHK022::(rplNp-GFPmut2) ∆rpoS	This study; promoter from Zaslaver et al, 2006	rplN ∆rpoS
MG1655 / pUA66-rplNp-GFPmut2	Zaslaver et al, 2006	rplN Pl.
MG1655 ∆rpoS / pUA66-rplNp-GFPmut2	This study; promoter from Zaslaver et al, 2006	rplN Pl. ∆rpoS
MG1655 attBHK022::(rpmBp-GFPmut2)	This study; promoter from Zaslaver et al, 2006	rpmB
MG1655 attBHK022::(rpsBp-GFPmut2)	This study; promoter from Zaslaver et al, 2006	rpsB
MG1655 attBHK022::(rpsBp-GFPmut2) ∆rpoS	This study; promoter from Zaslaver et al, 2006	rpsB ∆rpoS
MG1655 attBHK022::(rrnBp-GFPmut2)	This study; promoter from Zaslaver et al, 2006	rrnB
MG1655 attBHK022::(rrnBp-GFPmut2) ∆rpoS	This study; promoter from Zaslaver et al, 2006	rrnB ∆rpoS
MG1655/pUA66-argCp-GFPmut2	Zaslaver et al, 2006	argC Pl.
MG1655/pUA66-bolAp-GFPmut2	Zaslaver et al, 2006	bolA Pl.
MG1655/pUA66-codBp-GFPmut2	Zaslaver et al, 2006	codB Pl.
MG1655 ∆rpoS/pUA66-codBp-GFPmut2	This study; plasmid from Zaslaver et al, 2006	codB Pl. ∆rpoS
MG1655/pUA66-gadXp-GFPmut2	Zaslaver et al, 2006	gadX Pl.
MG1655 ∆rpoS/pUA66-gadXp-GFPmut2	This study; plasmid from Zaslaver et al, 2006	gadX Pl. ∆rpoS
MG1655/pUA66-glgSp-GFPmut2	Zaslaver et al, 2006	glgS Pl.
MG1655 ∆rpoS/pUA66-glgSp-GFPmut2	This study; plasmid from Zaslaver et al, 2006	glgS Pl. ∆rpoS
MG1655/pUA66-hslVp-GFPmut2	Zaslaver et al, 2006	hslV Pl.
MG1655 ∆rpoS/pUA66-hslVp-GFPmut2	This study; plasmid from Zaslaver et al, 2006	hslV Pl. ∆rpoS
MG1655/pUA66-hupAp-GFPmut2	Zaslaver et al, 2006	hupA Pl.
MG1655 ∆rpoS/pUA66-hupAp-GFPmut2	This study; plasmid from Zaslaver et al, 2006	hupA Pl. ∆rpoS
MG1655/pUA66-nrdHp-GFPmut2	Zaslaver et al, 2006	nrdH Pl.
MG1655/pUA66-nrdDp-GFPmut2	Zaslaver et al, 2006	nrdD Pl.
MG1655/pUA66-pliGp-GFPmut2	Zaslaver et al, 2006	pliG Pl.
MG1655/pUA66-pyrBp-GFPmut2	Zaslaver et al, 2006	pyrB Pl.
MG1655/pUA66-sbmCp-GFPmut2	Zaslaver et al, 2006	sbmC Pl.
MG1655/pTrc99a-trcp-GFPmut2	This study	pTrc99a-GFPmut2
MG1655/pTrc99a-trcp-mCherry2-L	This study	pTrc99a-mCherry2-L
MG1655/pTrc99a-trcp-mVenus	This study	pTrc99a-mVenus
**Chemicals, enzymes and other reagents**		
M9 salts	Sigma-Aldrich	M6030
MgSO_4_	Sigma-Aldrich	230391
CaCl_2_	Sigma-Aldrich	C4901
D-(+)-Glucose	Sigma-Aldrich	G7528
Kanamycin	Sigma-Aldrich	K1377
Ampicillin	Sigma-Aldrich	A0166
LB Broth (Miller)	Sigma-Aldrich	L3522
Hydrogen peroxide	Sigma-Aldrich	88597-F
**Software**		
Elveflow Smart Interface	https://elveflow.com/microfluidic-products/microfluidics-software/elveflow-software-sdk/	
µManager	https://micro-manager.org/	
deepMoMA	https://github.com/nimwegenLab/moma/releases/tag/v0.9.6	
R	https://www.r-project.org/	
RStudio	https://posit.co/download/rstudio-desktop/	
Optical Parameter Monitor (Thorlabs)	https://www.thorlabs.com/software_pages/ViewSoftwarePage.cfm?Code=OPM	
**Microscopy equipment**		
Nikon Ti-E	Nikon	
CFI Plan Apochromat Lambda DM ×100 objective	Nikon	
Hamamatsu Orca-Flash 4.0v3	Hamamatsu Photonics	
Thorlabs MWWHL4	Thorlabs	
Lumencor SpectraX	Lumencor	
459/526/596 nm triple-edge dichroic beamsplitter and 475/543/702 nm triple-band emission filter	Semrock	
495/605 dual-edge dichroic beamsplitter, and 527/645 dual-band emission filter	Semrock	
The-Cube	Life Imaging Systems	
**Other**		
SYLGARD 184	Dow	

### Bacterial strains and media

All strains used in this study are derivatives of *E. coli* K12 MG1655 (GCSC #6300), and are listed in the Reagents and tools table. Reporter constructs were obtained or derived from the Zaslaver library of *E. coli* native promoters (Zaslaver et al, 2006). In addition, we used synthetic promoters evolved in vitro in the same pUA66 plasmid backbone to study the expression of unregulated promoters (Wolf et al, 2015). The sequences of promoters used in this study are listed in the Dataset EV1. We used different fluorescent reporters for different promoters, i.e., either GFPmut2 (Zaslaver et al, 2006), mVenus (Cox et al, 2010), or mCherry2-L (Balleza et al, 2018). Figures are based on measurements obtained with plasmidic reporters and GFPmut2, unless stated otherwise in the caption.

In more detail, plasmid reporters with GFPmut2 were taken directly from the Zaslaver library and used for the promoters: *pyrB*, *argC*, *codB*, *rplN*, *hupA*, *hslV*, *bolA*, *pliG*, *gadX*, *nrdH*, *nrdD*, *glgS*, *sbmC*, and *Med3*. We verified that the plasmid copy number does not change between growth and starvation, and therefore does not play a role in the observed changes of volumic production (Appendix Fig. S5C,D). Chromosomal GFPmut2 reporters were constructed by integrating the plasmid fragment containing the promoter, ribosome binding site, and GFPmut2 protein at the phage HK022 locus *attB*, using the *clonetegration* method (St-Pierre et al, 2013). These chromosomal reporters were used for the ribosomal promoters (*rplN*, *rpsB*, *rpmB*, and *rrnB*), as well as two synthetic promoters (referred to as *Hi1* and *Med3*), and additionally *gshA* and *yrdC*. In addition, three strains were constructed with dual reporters (mVenus and mCerulean) for a given synthetic promoter (referred to as *Hi2*, *Med2*, and *Med3*) and an mCherry reporter of the RpoS regulatory activity (using the *bolA* promoter). In these strains, the dual reporters are integrated with diverging orientation at the phage HK022 locus *attB*. In more detail, each synthetic promoter was amplified from the corresponding pUA66 plasmid, together with the strong RBS sequence located downstream. Gibson assembly was used to prepare the *clonetegration* helper plasmid containing the two diverging promoter sequences separated by a random spacer, and the fluorescent proteins downstream (mVenus and mCerulean, amplified from plasmid pZS-124 (Cox et al, 2010)). Note that in the experiments reported in this article, we did not acquire the signal coming from mCerulean, but we did verify that it was not affecting other measurements (see Appendix section 1). A similar construct with the *bolA* promoter controlling the expression of an mCherry2-L protein (amplified from plasmid pEB2-mCherry2-L of Balleza et al. (2018)) was integrated in these dual-reporter strains at the phage 186 insertion site.

For all constructs integrated using the *clonetegration* protocol, the *kanR* resistance cassette was excised using transient FLP induction, and the final construct was checked by Sanger sequencing.

To analyse the regulatory role of the RpoS sigma factor, *ΔrpoS* strains were obtained from some of the strains mentioned above, using a P1 transduction of the *rpoS*::*kanR* loci from the Keio collection (Baba et al, 2006). The kanamycin resistance cassette was excised afterward using the pE-FLP helper plasmid (St-Pierre et al, 2013).

All experiments were done in M9 minimal media (Sigma-Aldrich), supplemented with 2 mM MgSO_4_, 0.1 mM CaCl_2_, and sugar as specified in the main text, typically 0.02% of glucose for the carbon-limited batch culture, and 0.2% of glucose for preculture and for the growth condition in microfluidic experiments with a hard switch. To starve bacteria during these experiments, we used the same minimal media not supplemented with any carbon source (referred to as “M9 Zero”). In experiments where an oxidative stress was applied, a 3% hydrogen peroxide (0.88 M) solution was diluted into M9 Zero at the appropriate concentration (0.65 or 0.75 mM). All experiments were carried out at 37 ^∘^C.

### Microfluidic procedures

For all microfluidic experiments, bacterial cultures used for inoculation were prepared as follows, unless otherwise noted. Bacteria were streaked onto LB agar plates from frozen glycerol stocks stored at −80 ^∘^C. Overnight preculture was grown from single colonies in M9 minimal medium supplemented with 0.2% of glucose. The next day, cells were diluted 100-fold into fresh medium and harvested after 4–6 h, at OD_600_ 0.05–0.15.

#### Microfluidic experiments with “hard switch” to starvation

In order to study the effect of starvation on bacterial gene expression dynamics at the single-cell level, we used a multiplexed DIMM as described previously (Julou et al, 2020). Several independent main channels are connected to the same DaW junction, allowing to multiplex up to eight different strains in parallel. In addition, each main channel features a low- resistance overflow channel which allows loading of the strains without mixing them between channels. We used a pressure controller (OB1 mk3, Elvesys) to switch the cells from exponential growth (M9 minimal media supplemented with 0.2% of glucose) to starvation (M9 Zero).

#### Microfluidic experiments with batch culture overflow

In order to compare the response of an abrupt entry into starvation with more gradual glucose exhaustion, we took advantage of the fact that the concentration of the limiting nutrient in a batch culture decreases gradually due to its uptake by the bacteria. When the culture reaches the stationary phase, bacteria eventually stop growing after exhausting nutrients. We thus continuously supplied the “overflow” from a batch bacterial culture as media input of the microfluidic chip, such that the cells inside the chip experience the same media as the bacteria in the flask, as pioneered by others to study stationary phase (Bakshi et al, 2021; Gefen et al, 2014).

In more detail, we seeded 10 μL of overnight culture into 100 mL of fresh M9 + 0.02% glucose. This concentration of glucose was chosen to make cell exhaust carbon before other limiting nutrients (Chubukov and Sauer, 2014). The batch culture was grown in a pressure-proof 100-mL glass bottle, with a custom cap allowing to connect tubing from the culture to the inlet of the microfluidic chip and to pressurize the bottle (1000 mbar during the whole experiment using an OB1 mk3 controller, Elvesys). The piece of fluoropolymer tubing used to connect the culture to the chip is chosen to minimize dead volume (ID: 0.25 mm, length ≈40 cm, hence ≈80 μL, which flows in 60 to 80 min at this pressure). We monitor, during the whole experiment, the optical density of the culture inside the flask, using an optical fiber connected to an OD probe immersed in the liquid (Ocean Optics, T300-RT-VIS-NIR); based on preliminary experiments, the mirror of the probe tip was removed, and the stainless-steel surface of the probe was polished in-house. The light source used for those OD measurements is an LEDD1B from Thorlabs, used at half its maximal intensity. The power is recorded using a PM100USB powermeter (Thorlabs), using the companion software Optical Power Monitor (Thorlabs). Because the precise time at which the transition to growth arrest happens is difficult to control experimentally, we typically ran the experiments for 120 h, in order to be sure to observe at least 60 h of starvation.

Since the Mother Machine design selects a biased sample of the growing bacterial population where non- or slow-growing cells are overrepresented, we used these overflow experiments to test the extent to which this bias affects our observations on gene expression during starvation. For this, we compared the concentration traces averaged over all cells, with another average where cells are weighted according to their expected number of cells in a corresponding batch culture. We focused on the time window spanning from one hour before the start of starvation to one hour after, over which the growth rate decreases to 0 (Appendix Fig. S14A). To compute weights, we counted, for each growth channel, the number of cells *n*_*s*_ present at the end (defined as entry into starvation) and the number of cells *n*_*t*_ pushed out of the channel during the growth rate transition phase; the corrected average is computed using weights (*n*_*s*_ + *n*_*t*_)/*n*_*s*_ for each channel. The comparison of the raw and corrected traces indicates that the effect of this sampling bias is so small that it can be neglected in our analysis (Appendix Fig. S15).

#### Microfluidic experiments with oxidative stress during starvation

Applying oxidative stress at a desired time during starvation and assessing survival by exposing bacteria in fresh media afterward requires multiple media switches during the experiment. To do so, we took advantage of another microfluidic chip design where eight independent DIMMs are multiplexed. These independent DIMMs are similar to the original DIMM design (Kaiser et al, 2018), but deprived of the mixing serpentine and with all waste channels connected together for convenience. We connected the first inlet of all the DIMMs (referred to as inlet A) to tubing pieces flowing the liquid of a single reservoir through a manifold, so that the same media is flown to all. To the second inlets (referred to as inlets B), we connected tubing coming from eight reservoirs pressurized together, so that each inlet can be supplied with different media. In order to switch four different media sequentially, the liquid of each reservoir needs to be exchanged while the corresponding inlets are inactive, as detailed in Appendix Table S4.

### Microscopy

We performed microfluidic experiments using an inverted Nikon Ti-E microscope, equipped with a motorized xy-stage and enclosed in a temperature incubator (TheCube, Life Imaging Systems). Focus was maintained using hardware autofocus (Perfect Focus System, Nikon). Images were recorded using a CFI Plan Apochromat Lambda DM ×100 objective (NA 1.45, WD 0.13 mm) and a CMOS camera (Hamamatsu Orca-Flash 4.0v3). The setup was controlled using *μ*Manager (Edelstein et al, 2010) and time-lapse movies were recorded with its multi-dimensional acquisition engine (sometimes customized using runnables).

Phase contrast images were acquired using a 100-ms exposure (Thorlabs MWWHL4, at 50% power). Images of mVenus fluorescence and mCherry fluorescence were acquired using a 459/526/596 nm triple-edge dichroic beamsplitter and 475/543/702 nm triple-band emission filter (Semrock), using 200 ms of exposure time and 32 and 8% of excitation intensity, respectively (Lumencor SpectraX, Teal LED, excitation filter 511/16 nm, and Yellow LED, excitation filter 578/21  nm). The use of the multiband filter allows to acquire both fluorescent signals without changing the filter sets, which increases the number of positions which can be measured at a given acquisition frequency. GFPmut2 fluorescence was acquired using a 495/605 dual-edge dichroic beamsplitter, and a 527/645 dual-band emission filter (Semrock), using 200 ms of exposure time and 8% of excitation intensity (Lumencor SpectraX, Cyan LED, excitation filter 470/24 nm).

In growth media, images were acquired every 3 min. To minimize phototoxicity, images were acquired only once every 12 min during carbon starvation, except the first hour after the switch to starvation media, where, in order to monitor transient dynamics with high time resolution, we still acquired images every 3 min.

### Phototoxicity

Under those illumination conditions, the ability of cells to regrow when exiting starvation was barely affected after 25 h of starvation (95 ± 5% of cells regrow; see Appendix Fig. S5A), indicating that phototoxicity is sufficiently low to not induce cell death in the first 25 h. However, although we did not observe cell lysis, we did observe a substantial increase in the non-regrowing fraction after 60 h of starvation compared to a control experiment without fluorescence illumination, as well as a detectable increase in the lag times to regrow for the remaining cells (Appendix Fig. S5A,B). Although gene expression may be affected by phototoxicity, we see no effects of fluorescence excitation on mortality for the first 25 h. Since most gene expression changes occur even earlier, i.e., during the first 5–10 h of starvation, there are no more significant changes in expression, we feel that it is unlikely phototoxicity is a major driver of the observed gene expression changes early in starvation.

### Image analysis

Automated image analysis was performed using MoMA (Kaiser et al, 2018). For datasets acquired in the later part of this project, we used a new version of the software developed internally, called deepMoMA (available at https://github.com/nimwegenLab/moma/releases/tag/v0.9.6, and relevant segmentation models at https://github.com/nimwegenLab/moma-model). The main difference between MoMA and deepMoMA is that segmentation hypotheses are generated using probability maps produced by a U-Net segmentation model trained for this purpose; cost functions used for tracking by global optimization were adapted as necessary.

Since the cell diameter fluctuates very little in a given condition, the cell length measured by MoMA is used as a proxy for the cell volume; accordingly, concentrations of fluorescent proteins are expressed in FP/μm. The estimation of the number of fluorescent proteins from fluorescence intensity is described in detail in the Appendix section 1.

Raw images from the experiments were stored on a centralized storage, and preprocessed before being analysed with MoMA. Empty growth channels, as well as those featuring structural defects or cells being growth-arrested from the start, were excluded from the analysis. Summary of the final number of cells observed during the experiments for each strains can be found in Appendix Table S6.

### Correcting for measurement noise using Gaussian process priors

Our experiments provide time course measurements of estimated total fluorescence and size of each cell in the microfluidic device, and from these measurements, we want to infer how the instantaneous growth rates and volumic protein production rates smoothly fluctuate with time in each cell. However, since each cell size and fluorescence measurement is affected by unavoidable measurement noise, and we a priori have little information about the ways in which true growth and volumic production rates may vary with time, inferring the underlying instantaneous rates and assigning error bars to these inferences is a challenging inference problem.

To address this challenge, our lab developed a Bayesian procedure which uses Gaussian process priors for both the volumic production rate and growth rate, called RealTrace (Kscheschinski et al, 2025), which is available and documented at https://github.com/nimwegenLab/RealTrace. The basic idea behind RealTrace is that, while measurement errors are independent for each measurement, the underlying true growth and volumic production rates vary continuously with unknown means, variances, and correlation times. These assumptions are formalized in a Bayesian procedure using maximum entropy process priors on the time-dependent growth and volumic fluorescence production rates. RealTrace first optimizes the parameters of the maximum entropy process prior and measurement errors, i.e., finding the parameters that maximize the likelihood of all measurements in a given experiment, and then uses this maximum likelihood prior to calculate the posterior distributions over cell sizes, fluorescence concentration, instantaneous growth rate, and instantaneous volumic fluorescence production rates for each cell at each time point.

For experiments, such as ours, in which growth conditions are changed dramatically during the experiments, causing large changes in the distribution of instantaneous growth and production rates, the accuracy of RealTrace inferences can be improved by using priors with different parameters for different time segments of the experiment, and the RealTrace software offers an option allowing different priors for different time segments.

For each promoter, we used this feature of RealTrace to use one set of prior parameters for the exponential growth phase and one for the stationary phase. The prior’s parameters for the exponential phase were simply fitted from all data of the experimental growth phase of the experiment, while for the starvation phase, we first fitted only the measurement error size from the later parts of the stationary phase (where production rates do not vary much) and then fitted all other parameters of the prior on data from the entire stationary period. This more complex procedure was used to obtain more accurate estimates of the measurement noise, which otherwise would be affected by the rapid and dramatic changes in volumic production early in the stationary phase.

Using these priors, we then obtain posterior distributions for the fluorescence level, length, growth rate, and volumic fluorescent protein production rate of each cell at each time point. These results are the data that we use throughout this study. Finally, we found that a small fraction of data points (approximately 10 000 out of 5 682 000 data points, i.e., <0.2%) were affected by segmentation errors in the deepMoMA image analysis step that escaped attention during curation (e.g., two cells merging into one), and we filtered these out by hand.

### Quantification of fluorescent protein degradation

#### Experimental protocol

We set out to measure the rate of degradation of our fluorescent proteins by performing experiments at very low exposure frequency, so as to avoid a fluorescence loss due to photobleaching. For this, we used *E. coli* strains transformed with a pTrc99a plasmid containing either mVenus, mCherry2-L, GFPmut2, or mCerulean under the control of an IPTG-inducible promoter. We conducted Mother Machine experiments with those strains, switching them from M9 + 0.2% glucose supplemented with 500 μM IPTG to M9 zero (i.e., without IPTG). During starvation, we acquired fluorescence images only every 3 or 6 h, thus making the effect of photobleaching negligible. The decrease of fluorescence signal can therefore be assumed to be only due to protein degradation (mediated or not by proteases). Total fluorescence of single cells was recorded in this way for 30 and 60 h, respectively, with 3-h intervals for the 30 h time course and 6-h intervals for the 60 h time course, so that we typically had ten fluorescence frames per single cell per experiment (see Appendix Fig. S2A for example time traces).

#### Estimation of degradation rates

For each cell and each consecutive pair of time points, we estimated the degradation rate by taking the discrete derivative in log-fluorescence and then averaged these across all cells at each time point. In Appendix Fig. S2B, we can see that the resulting estimated decay rates are decreasing approximately exponentially with time during the starvation period (i.e., decreasing approximately linearly on a logarithmic scale).

The degradation rate *γ* is thus a time-dependent variable during starvation that decreases approximately as $\gamma (t)={\gamma }_{0}{e}^{-{\omega }_{\gamma }t}$ where *γ*_0_ is the degradation rate at entry into starvation and ${\omega }_{\gamma }$ is the exponential decay rate of *γ*. These parameters are obtained from exponential fits of the instantaneous derivative of fluorescence along time (Appendix Table S5).

### Measurements of photobleaching rates

We measured photobleaching rates of the different fluorescent proteins using the same pTrc99a inducible plasmids as for measuring proteolysis. After inducing the expression of the fluorescent proteins with a media containing M9 + 0.2% glucose and IPTG 500 μM, we switched the cells to media without inducer, either M9 + 0.2% glucose (to measure photobleaching during exponential growth), or M9 zero (to measure photobleaching during stationary phase). We measured the fluorescence as a function of time, using our default illumination settings. The procedure for estimating photobleaching rates from time series of fluorescence levels is described in the Appendix section 2.

## Supplementary information

Appendix

Peer Review File

Dataset EV1

## Data Availability

The datasets and computer code produced in this study are available in the following repositories: Image datasets analysed with MoMA: BioImage Archive https://doi.org/10.6019/S-BIAD2506. Image analysis output, analysis scripts and simulations: Zenodo https://doi.org/10.5281/zenodo.18059846. Mass spectrometry files: MassIVE (accession code MSV000095680) https://doi.org/10.25345/C53776653. The source data of this paper are collected in the following database record: biostudies:S-SCDT-10_1038-S44320-026-00226-5.
