# Supplementary material for: E. coli prepares for starvation by dramatically remodeling its proteome in the first hours after loss of nutrients
Source: Mol Syst Biol. 2026 Jul 1;22(10):1531–50. doi: 10.1038/s44320-026-00226-5 (PMC13639054; doi:10.1038/s44320-026-00226-5)
Supplement: Supplementary file 1 [file 44320_2026_226_MOESM1_ESM.pdf]

Appendix to the article "*E. coli* prepares for starvation by dramatically remodeling its proteome in the first hours after loss of nutrients"

1 Estimation of fluorescent proteins levels 3
1.1 Accounting for autofluorescence and crosstalk between fluorescence channels 3
1.2 Fluorescence signal conversion to absolute numbers 3
1.3 Accounting for maturation of fluorescent proteins 4
2 Estimation of photobleaching rates 5
3 Calculation of production traces and concentration traces 7
3.1 Inference of true instantaneous production 7
3.2 Integration of concentration traces 9
3.3 Dynamics of the protein amount produced during a given time window 11
4 Dynamics of volumic production in late starvation 11
5 Regulators of synthetic promoters 13
6 Comparison of microfluidic measurements with transcriptomics 14
7 Quantitative proteomics measurements of protein levels 15
7.1 Sample Preparation 15
7.2 LC-MS analysis 16
7.3 Protein Identification and Quantification 16
7.4 Proteomics fold change calculation from literature 17
7.5 Interpretation of the proteomics measurements 18
8 A simple mathematical model capturing the diversity of concentration dynamics 18

List of Appendix Figures

Appendix Figure S1. Inference of instantaneous growth rate and production rate from noisy traces of cell length and fluorescence 24

Appendix Figure S2. Estimation of the degradation rates of fluorescent proteins 25

Appendix Figure S3. Comparison of fluorescent reporters and maturation dynamics 26

Appendix Figure S4. Growth dynamics upon entry into starvation 27

Appendix Figure S5. Experimental setup controls for the hard switch experiments 28

Appendix Figure S6. Selection of native promoters 29

Appendix Figure S7. Production dynamics of individual cells for all promoters at the onset of starvation 30

Appendix Figure S8. Production dynamics of all promoters at the onset of starvation 31

Appendix Figure S9. Comparison with the relative expression levels measured by transcriptomics . . . 32
Appendix Figure S10. The late dynamics of volumic production is promoter dependent . . . 33
Appendix Figure S11. Analysis of protein concentration changes between growth and starvation using quantitative proteomics . . . 34
Appendix Figure S12. Analysis of the overall agreement in expression fold-changes between growth and starvation as measured by proteomics and fluorescent reporters . . . 35
Appendix Figure S13. Expression levels of a selection of reporters in the ΔrpoS mutant . . . 36
Appendix Figure S14. Comparison of a hard switch with a gradual entry into stationary phase . . . 37
Appendix Figure S15. Controlling for population sampling bias in the Mother Machine during overflow experiments . . . 38
Appendix Figure S16. Comparison of protein concentration dynamics for the hard switch and gradual entry into stationary phase . . . 39
Appendix Figure S17. Changes of concentration upon entry into starvation are consistent across individual cells . . . 40
Appendix Figure S18. Fraction of explained variance ρ for target promoters of the LexA regulon under increasing subinhibitory doses of ciprofloxacin . . . 41
Appendix Figure S19. Relative contributions to protein concentrations from protein production during different periods . . . 42
Appendix Figure S20. Gene expression dynamics of RpoS targets upon entry into starvation under increasing inhibition of protein synthesis . . . 43
Appendix Figure S21. Impact of gene expression perturbations on survival to oxidative stress during starvation . . . 44

List of Appendix Tables

Appendix Table S1. Experimentally measured bleaching rates and corresponding correction . . . 7
Appendix Table S2. Predicted lower bounds on the fold-change f_0 in concentration for the different fluorescent reporters . . . 20
Appendix Table S3. Parameters of the concentration dynamics model . . . 21
Appendix Table S4. Timing of media switches for the microfluidic experiments with oxidative stress . . . 21
Appendix Table S5. Parameters of the time-dependent protein degradation rates . . . 21
Appendix Table S6. Summary of cell numbers in the different experiments . . . 22

1 Estimation of fluorescent proteins levels

1.1 Accounting for autofluorescence and crosstalk between fluorescence channels

For each fluorescent protein, we measured the corresponding levels of autofluorescence per μm cell length of our wild type strain in the same experimental conditions as used for gene expression estimation. The estimated autofluorescence per unit length was found to only depend on the nature of the fluorescent protein (mCherry, GFP, mVenus), and not on the conditions (growth or starvation). In our analysis, we estimate the autofluorescence of a cell by its length and always first subtract this estimated autofluorescence from the measured fluorescent levels.

In addition, since we use multi-band fluorescence filters and since the excitation spectra of mCherry2 overlaps with the excitation spectra of mVenus, and similarly between mVenus and mCerulean, we checked whether the readout in one channel was affected by the level of the other when using dual fluorescent reporters. With *E. coli* strains transformed with a pTrc99a plasmid containing either mVenus, mCherry2, mCerulean, we could measure the amount of fluorescence coming from mVenus protein in the mCherry2 channel or mCerulean protein in the mVenus channel, and reversely. As expected from the spectra, we found no contamination coming from mVenus signal into the mCherry2 channel, and no contamination coming from mCerulean into the mVenus channel. Nevertheless, we found that the mCherry2 signal appeared in the mVenus channel at 21% of its level in the mCherry2 channel. We thus compensate for this, by subtracting this fraction of mCherry signal from the total mVenus signal, in the cases where we used dual fluorescent reporter strains. It is important to note that, due to the much higher intensity obtained with mVenus compared to mCherry in a given experiment, the contribution of mCherry in the mVenus signal was always very small.

1.2 Fluorescence signal conversion to absolute numbers

For each cell in each image we estimate a total fluorescence by fitting a Cauchy-shaped distribution with offset to the total intensities of pixel columns across the segment of the image containing the cell, and then corrected for cell autofluorescence, as described in (Kaiser et al. 2018).

Throughout the article, we express the fluorescence levels in units of number of fluorescent proteins n. To obtain this number n, we need to convert the autofluorescence-corrected fluorescence intensity I we get from our measurements (as measured by our setup, using specific illumination parameters) using a conversion factor α, corresponding to the fluorescence emitted by a single fluorescent protein, i.e. $n = I/\alpha$.

For GFP, we have previously estimated α from the fluctuations of fluorescence levels in newborn sibling pairs (Kaiser et al. 2018). We used this estimate $1/\alpha_{\text{Kaiser}} = 0.0361$ to infer the conversion factors in our experiments as well. In order to account for the fact that the illumination parameters of our experiments are not always the same, we assumed that α scales linearly with the light dose $D = T \times L \times N$ where T is the exposure time in ms, L is the excitation power of the light source and N is the

attenuation factor of the (facultative) neutral density present in the excitation light path. We therefore have, for GFP in our experiment

$$\alpha_{\mathrm{GFP}} = \frac{D_{\mathrm{GFP}}}{D_{\mathrm{Kaiser}}} \alpha_{\mathrm{Kaiser}}$$

Given our illumination settings (see Methods), we have $D_{\mathrm{GFP}} = 200 \times 0.08 \times 1 = 16$ instead of $D_{\mathrm{Kaiser}} = 2000 \times 0.16 \times \frac{1}{4} = 80$. We can thus use the conversion factor $1/\alpha_{\mathrm{GFP}} = 0.1805$ for GFP in our experiments.

To find the conversion factor for the other fluorescent proteins (mCherry noted RFP, mVenus noted YFP), we assumed that, at steady state exponential growth, the number of fluorescent proteins present in the cells is dependent on the promoter, but independent of the reporter protein. We took advantage of sets of experiments where the same promoter drives the expression of different reporter fluorescent proteins, and write $I_{\mathrm{RFP/YFP}}/\alpha_{\mathrm{RFP/YFP}} = n = I_{\mathrm{GFP}}/\alpha_{\mathrm{GFP}}$, which in turn means

$$\frac{1}{\alpha_{\mathrm{RFP/YFP}}} = \frac{I_{\mathrm{GFP}}}{I_{\mathrm{RFP/YFP}}} \frac{1}{\alpha_{\mathrm{GFP}}}$$

Note that we first need to convert the intensity to single-reporter equivalents if one of the two reporters is expressed from a plasmid. This is due to the fact that plasmids are present in multiple copies per cell, and therefore we expect n to be higher in those strains compared to chromosomal reporter strains, by a factor equal to the plasmid copy number. Using the plasmid copy number estimated in Appendix Fig. S5D, we find $1/\alpha_{\mathrm{RFP}} = 0.495$ and $1/\alpha_{\mathrm{YFP}} = 0.105$.

We stress that these estimations of the conversion factors α have limited accuracy. The main aim of reporting expression levels in protein number equivalents, as opposed to using 'arbitrary units', is that it provides a reasonable indication of the order of magnitude of the absolute number of proteins present in the cell. The relative expression levels across time of the reporters are of course not affected at all by this potential inaccuracy in the estimate of absolute levels.

1.3 Accounting for maturation of fluorescent proteins

To minimize the effects fluorescent protein maturation we chose fast maturing variants (half-time for maturation at 37°C: 5.6 min for GFPmut2, 9.6 min for mVenus, and 22.8 min for mCherry2-L; Balleza et al. (2018)). To assess the effect of maturation delay on the inferred production dynamics, we developed an analytical procedure to correct for either both protein degradation and maturation, or only protein degradation (Appendix section 3). We then compared inferred production traces with and without maturation correction using data from experiments where the same promoter was used with different fluorescent proteins (Appendix Fig. S3). As expected, correcting for maturation shifts the peak of production to the left, and this effect is more pronounced for mCherry, followed by mVenus, and only minimal for the fast maturing GFPmut2. Notably, after correction for maturation the three reporters show very similar dynamics with peaks in production at roughly the same time. However, we

observe some discrepancy in the fold-changes, with mVenus and mCherry showing larger fold-changes than GFPmut2, which may be due to differences in mRNA stability and translation rate of these reporters.

Most of our results are based on data acquired with GFPmut2 and the effect of maturation is so small for this reporter that we neglect it (because the correction would introduce more measurement noise), whereas for mVenus and mCherry the effect is sufficiently large that we do explicitly correct for maturation (see Appendix section 3).

2 Estimation of photobleaching rates

Using the results from the above-mentioned experiments, we estimated the photobleaching rates for our different fluorescent proteins (mCherry, mVenus, GFPmut2) in the different experimental conditions (exponential growth and starvation settings). Because the total decay in fluorescence is the result of both photobleaching and degradation of the fluorescent proteins, we needed to account for protein degradation in order to estimate the photobleaching rate of each fluorescent protein. Once the degradation was estimated (as described in the Methods section of the main text), we used a Bayesian inference procedure to estimate the most likely photobleaching across all observations. The resulting bleaching rates were then used when running RealTrace on our data.

In order to quantify photobleaching from fluorescent traces acquired in these experiments, we first calculate and subtract at every time point the amount of protein that has been degraded from the start of the observation. The total fluorescence decreases according to the differential equation

$$\frac{dF(t)}{dt} = -\left(\beta + \gamma(t)\right) F(t),$$

with $\gamma(t) = \gamma_0 e^{-\omega t}$ (see Methods). The solution of this differential equation is

$$F(t) = F_0 e^{-\frac{\gamma_0}{\omega}(1-e^{-\omega t})-\beta t} \tag{S1}$$

We define $F_\beta(t)$ as the amount of fluorescence at time t in the absence of protein degradation, i.e. if fluorescence decayed only due to photobleaching. Using equation S1 we have

$$F_\beta(t) = F(t) e^{\frac{\gamma_0}{\omega}(1-e^{-\omega t})} \tag{S2}$$

so that $F_\beta(t) = F_0 e^{-\beta t}$. We then use the fluorescence values $F_\beta(t)$ computed with Eq. S2 to estimate the photobleaching rate.

In particular if we define:

- t_{ci} the time at which the ith measurement of cell c took place.
- $f_{ci} = \log(F_\beta(t_{ci}))$ the logarithm of the degradation-corrected measured fluorescence value in cell c at time t_{ci}.
- ϕ_c the initial log fluorescence of cell c.
- α the photobleaching rate.
- σ the noise of the log-fluorescence measurement.

then the probability of all the fluorescence measurements given α, the ϕ_c and σ is given by

$$
\begin{aligned}
P(D|\boldsymbol{\phi_c}, \alpha, \sigma) &= \prod_{c=1}^{C} \prod_{i=1}^{N_c} \frac{1}{\sigma\sqrt{2\pi}} \exp\left[-\frac{1}{2}\left(\frac{f_{ci} - \phi_c + \alpha t_{ci}}{\sigma}\right)^2\right] \\
&\propto \frac{1}{\sigma^N} \exp\left(-\frac{1}{2}\sum_{c,i}\left(\frac{f_{ci} - \phi_c + \alpha t_{ci}}{\sigma}\right)^2\right),
\end{aligned}
\tag{S3}
$$

where $\boldsymbol{\phi_c}$ is the vector of ϕ_c, C is the number of cells, N_c is the number of data points in cell c, and $N = \sum_c N_c$ is the total number of data points in our data set.

Marginalizing over all ϕ_c using uniform priors and over σ using a scale-prior $P(\sigma) = \frac{1}{\sigma}$ we obtain:

$$
P(D|\alpha) \propto \left(\sum_{c=1}^{C} N_c \text{var}(f_c + \alpha t_c)\right)^{-\frac{N-C}{2}},
$$

where $\text{var}(f_c + \alpha t_c)$ denotes the variance of the quantity $f_{ci} + \alpha t_{ci}$ across the N_c timepoints of cell c.

The optimal bleaching rate α_* and its error-bar (i.e. standard deviation of the posterior) σ_α can be expressed in terms of the weighted sums of variances

$$
V_f = \sum_{c=1}^{C} N_c \text{var}(f_c), \tag{S4}
$$

$$
V_t = \sum_{c=1}^{C} N_c \text{var}(t_c), \tag{S5}
$$

and the weighted sum of covariances

$$
V_{ft} = \sum_{c=1}^{C} N_c \text{var}(f_c, t_c). \tag{S6}
$$

In particular, we find

$$
\alpha^* = -\frac{V_{ft}}{V_t} \tag{S7}
$$

with error-bar

$$
\sigma_\alpha = \sqrt{\frac{V_f V_t - V_{ft}^2}{V_t^2 (N - C)}}. \tag{S8}
$$

The resulting photobleaching rates α_* used for the inference with RealTrace are reported in Appendix Table S1.

Fluo	c_{step}	$\beta(\text{h}^{-1})$	Correction coeff.
GFP	Growth	0.026	1.04
mCherry	Growth	0.20	1.29
mVenus	Growth	0.28	1.4
GFP	Starvation	0.0041	-
mCherry	Starvation	0.031	-
mVenus	Starvation	0.044	-

Appendix Table S1. Experimentally measured bleaching rates and corresponding correction. The correction factor is used to estimate the total concentration during exponential growth from the observed fluorescence concentration.

3 Calculation of production traces and concentration traces

3.1 Inference of true instantaneous production

To infer a volumic production rate $q^{\text{inf}}(t)$, RealTrace assumes that the total fluorescence $F(t)$ of the cell evolves according to

$$\frac{dF(t)}{dt} = q^{\text{inf}}(t)V(t) - \beta F(t), \tag{S9}$$

where β is the bleaching and constant degradation rate, i.e. RealTrace assumes that degradation occurs at a constant rate during the experiment. In terms of the concentration of visible protein $f(t) = \frac{F(t)}{V(t)}$ we have,

$$\frac{df}{dt} = q^{\text{inf}}(t) - (\lambda(t) + \beta)f(t), \tag{S10}$$

where $\lambda(t)$ is the instantaneous growth rate. Thus, RealTrace does not account for time-dependent degradation and also ignores the delay due to maturation of fluorescent proteins. We, therefore developed separate procedures to account for the the effects of maturation and time-dependent degradation on the inferred volumic production rate q^{inf}, and infer the true production rate q.

Correction for maturation and degradation

We denote the concentration of immature protein by $u(t)$, the maturation rate by κ, and denote by $\gamma(t)$ the time-dependent protein degradation rate, which we inferred in calibration experiments as described in the Methods section of the main article.

The dynamics of the immature protein concentration is given by

$$\frac{du(t)}{dt} = q(t) - (\kappa + \lambda(t) + \gamma(t))\, u(t).$$

We can use this equation to write the production $q(t)$ in terms of $u(t)$:

$$q(t) = \frac{du(t)}{dt} + (\kappa + \lambda(t) + \gamma(t))\, u(t).$$

Second, the dynamics of fluorescence concentration f is given by

$$\frac{df}{dt} = \kappa u(t) - (\gamma(t) + \lambda(t) + \beta)\, f(t),$$

where the first term corresponds to maturation of immature fluorescent proteins. Noting that we run RealTrace with the parameter β set to the bleaching rate, and comparing this equation to Eq. (S10), we obtain

$$q^{\text{inf}}(t) = \kappa u(t) - \gamma(t) f(t),$$

which holds at each time t. Using this, we can write $u(t)$ as a function of $q^{\text{inf}}(t)$ as

$$u(t) = \frac{1}{\kappa}\left[q^{\text{inf}}(t) + \gamma(t) f(t)\right]$$

If we substitute this into the equation for $q(t)$ we obtain

$$q(t) = \frac{1}{\kappa}\frac{d}{dt}\left[q^{\text{inf}}(t) + \gamma(t) f(t)\right] + \frac{\kappa + \lambda(t) + \gamma(t)}{\kappa}\left[q^{\text{inf}}(t) + \gamma(t) f(t)\right]. \tag{S11}$$

So far these equations are exact. While we have estimated the time dynamics of the degradation $\gamma(t)$, RealTrace only gives us estimated values for $q^{\text{inf}}(t)$, and $f(t)$ at the measurement time points t_i, and no values for their derivatives. We thus decided to estimate $q(t)$ at time points $t_i + dt/2$ halfway between time points t_i and $t_{i+1} = t_i + dt$, by discrete derivatives between these two time points as follows:

$$\begin{aligned} q(t_i + dt/2) \approx & \frac{1}{\kappa dt}\left[q_{i+1} - q_i + \gamma_{i+1} f_{i+1} - \gamma_i f_i\right] \\ & + \frac{1}{2\kappa}\left[(\lambda_{i+1} + \gamma_{i+1} + \kappa)(q_{i+1} + \gamma_{i+1} f_{i+1}) + (\lambda_i + \gamma_i + \kappa)(q_i + \gamma_i f_i)\right], \end{aligned} \tag{S12}$$

where $q_i = q(t_i)$, $q_{i+1} = q(t_i + dt)$ and so on.

We use this equation together with the maturation rates κ measured experimentally in Balleza et al. (2018) to estimate the true production rate $q(t)$ from the production rate $q^{\text{inf}}(t)$ and fluorescence concentration $f(t)$ estimated by RealTrace. Notably, we perform this for calculation the measurements with fluorescent proteins mVenus and mCherry. For GFPmut2, the maturation rate κ is so high that the effect of maturation is low and we decided to avoid the need for the approximation (S12).

Correction for degradation only

For the fast folding proteins GFPmut2, on which most our results are based, the production and concentration traces are very similar with and without taking the maturation dynamics into account (Appendix Fig. S3). Since the correction for maturation (S12) involves an approximation with discrete derivatives, we decided to use a more exact estimate that only corrects for the time-dependent degradation for all our observations with GFPmut2 (which is most of our results).

If we approximate maturation as instantaneous, the dynamic of the fluorescence concentration f reads

$$\frac{df}{dt} = q(t) - (\gamma(t) + \lambda(t) + \beta)f(t).$$

Again, comparing this equation to (S10) that RealTrace assumes, we obtain

$$q(t) = q^{\text{inf}}(t) + \gamma(t)f(t). \tag{S13}$$

Whenever the fluorescent reporter is GFPmut2, we use this equation to infer the true production $q(t)$ as shown in, *e.g.*, Fig. 1C and Appendix Fig. S8, using the estimates of $q^{\text{inf}}(t)$ and $f(t) = \frac{F(t)}{V(t)}$ given by RealTrace, and the degradation rate $\gamma(t) = \gamma_0 e^{-\omega t}$ that we inferred in calibration experiments. For reporters based on mCherry2-L and mVenus, with longer maturation times, we take into account the maturation dynamics as explained above.

Finally, we note that for observations during the exponential growth phase, we assume that the degradation rate $\gamma(t)$ equals the degradation rate γ_0 at the start of the starvation phase. Although we do not know the true degradation rate during exponential phase, since in exponentially growing cells the role of degradation is negligible compared to dilution due to growth, we expect the relative error introduced by this assumption to be very small.

3.2 Integration of concentration traces

For each reporter, we want to estimate the true concentration of proteins $C(t)$ as a function of time t in stationary phase, i.e. as plotted in Fig. 2A and Appendix Fig. S17A. Notably, we cannot simply take the fluorescence concentration since the true protein concentration includes both unbleached and bleached proteins. We thus need to integrate the concentration dynamics consisting of the true volumic production rate $q(t)$ and protein degradation at rate $\gamma(t)$ during stationary phase. That is, the dynamics of the true protein concentration $C(t)$ reads

$$\frac{dC(t)}{dt} = q(t) - \gamma(t)C(t),$$

where $q(t)$ is the true production rate which is corrected for bleaching, degradation and possibly maturation (for mVenus and mCherry), as described in the last sections.

Correcting the concentration for photobleaching during exponential growth

Our integration approach requires estimating the total concentration of fluorescent proteins $C(0)$ at the start of stationary phase $t = 0$, i.e. including both unbleached and bleached proteins. However, our measurements only give us estimates of the concentration $C_u(0) = F(0)/V(0)$ of unbleached proteins at the start of stationary phase. To estimate $C(0)$, we thus need to estimate the ratio of bleached and unbleached

proteins at the start of stationary phase. Note that during exponential growth, the concentration $C(t)$ evolves according to

$$\frac{dC(t)}{dt} = q(t) - (\lambda(t) + \gamma_0)C(t), \tag{S14}$$

where $\lambda(t)$ is the instantaneous growth rate and γ_0 is the degradation rate during exponential growth. Similarly, the concentration of unbleached proteins $C_u(t) = F(t)/V(t)$ evolves according to

$$\frac{dC_u(t)}{dt} = q(t) - (\lambda(t) + \gamma_0 + \beta)C_u(t). \tag{S15}$$

Both the volumic production $q(t)$ and the growth rate $\lambda(t)$ will fluctuate around averages q and λ respectively, and if we approximate them as approximately constant, we find steady-state values $C = Q/(\lambda + \gamma)$ and $C_u = Q/(\lambda + \gamma_0 + \beta)$. We thus find that approximately,

$$C = C_u \frac{\lambda + \gamma_0 + \beta}{\lambda + \gamma_0} \approx C_u \frac{\lambda + \beta}{\lambda}, \tag{S16}$$

where the last approximation follows from the fact that the degradation rate γ_0 is much smaller than the dilution rate λ. With an average growth rate of approximately ≈ 0.7 h^{-1} and the bleaching rates estimated as described in the previous section, we obtain the correction coefficients shown in Appendix Table S1.

We use this correction to estimate the concentration at the beginning of the cell cycles during growth and at the first observation in starvation. In the case of reductive division, where the cell cycle starts after the beginning of growth arrest, we cannot use this factor. In this case, we simply use the last (corrected) concentration of the parent cell as a starting observation.

Integrating instantaneous production from initial fluorescence value

In order to obtain the level of proteins in the cells at time t, integrating the true production and taking into account the degradation of both already-bleached and unbleached proteins, we need to integrate

$$\frac{dC(t)}{dt} = q(t) - \gamma(t)C(t) \tag{S17}$$

Since the estimated true production $q(t)$ varies in an unknown way from time-point to time-point, this integration must be done numerically and we use the following iterative scheme:

$$C_{i+1} = C_i + [q_i - \gamma_i C_i]\, dt_i, \tag{S18}$$

where C_i, q_i and γ_i are the concentration, volumic production and degradation rate at time t_i, respectively, and dt_i is the time interval $t_{i+1} - t_i$.

At time $t = 0$, we use the value $C(0)$ calculated from the bleaching correction coefficient if the cells are coming from exponential growth (see Appendix Table S1),

or the last calculated concentration of the parent cells if they come from a reductive division during starvation.

3.3 Dynamics of the protein amount produced during a given time window

We now want to estimate, at each time point, what fraction of fluorescent proteins in the cells come from different periods in the past, *e.g.* the burst of expression (during the entry into starvation). For this, we need to track the evolution of the pool of proteins produced during a given time window. As in the previous section, this to calculate the dynamics of the total amount of protein $F(t)$ in terms of the true production $q(t)$ and time-dependent degradation $\gamma(t)$, rather than directly using the volumic production rate $q^{\text{inf}}(t)$ inferred by RealTrace.

Similar to equation (S17), the dynamics of the total protein amount $F(t)$ is given by

$$\frac{dF(t)}{dt} = q(t)V(t) - \gamma(t)F(t), \tag{S19}$$

where $V(t)$ is the size of the cell at time t.

Since we have no analytical form for the production rate $q(t)$, we integrate this equation numerically instead, using an iterative scheme:

$$F_{i+1} = F_i + [q_i V_i - \gamma_i F_i]\, dt_i, \tag{S20}$$

with F_i, V_i and γ_i the total protein amount, cell size, and degradation rate at time t_i. The time interval dt_i is again $dt_i = t_{i+1} - t_i$.

From this, we can calculate, at any given time point, the amount of protein coming from a given period of time $[t_1, t_2]$. At time t, the amount of protein that remains and has been produced during this period of time $F^{[1,2]}(t)$ can be calculated iteratively as follows. Let $F_i^{[1,2]}$ denote $F^{[1,2]}(t_i)$. We initialize $F_0^{[1,2]} = 0$ and then iterate

$$F_{i+1}^{[1,2]} = F_i^{[1,2]} + \left[\Theta(t_i - t_1)\Theta(t_2 - t_i)q_i V_i - \gamma_i F_i^{[1,2]}\right] dt_i, \tag{S21}$$

where $\Theta(x)$ is the Heaviside step-function that is zero when $x < 0$ and one otherwise.

We can now follow the fraction $F_{\text{frac}}(t)$ of the total protein at time t that was produced between t_1 and t_2.

$$F_{\text{frac}}(t) = \frac{F^{[1,2]}(t)}{F(t)},$$

where $F(t)$ is the total amount of protein at time t. Calculating this fraction for three time windows: Growth ($t \leq 0$ h), Early Starvation ($0 \text{ h} < t \leq 10$ h), and Late Starvation ($t > 10$ h), we obtain the Muller plots represented on Fig. 5A and Appendix Fig. S19.

4 Dynamics of volumic production in late starvation

In contrast to the previously reported Constant Activity in Stationary Phase, we observe no period during starvation where gene expression remains constant. From

the maximal level of production (either the level during exponential growth or the top of the peak when there is a burst early in the starvation phase), volumic production initially decreases fast for all promoters, after which it decreases more slowly. The overall dynamics of volumic production over time is reasonably well fitted by a bi-exponential decay (Appendix Fig. S10A, fitting procedure described below), although this fit does not extend late in starvation for many promoters because, due to errors in the estimation, the production is estimated to be negative at some time points. Note that when production is very low, fluctuations of the autofluorescence and of the degradation of fluorescent proteins, as well as measurement errors of the total fluorescence level, will all substantially affect the accuracy of the estimation of the instantaneous production.

Exponential fit of the production decay

In order to fit the decay of production with a bi-exponential function, we express the production q_i, i.e. the 'true' production at time t_i, as

$$q_i = ae^{-\lambda_1 t_i} + be^{-\lambda_2 t_i}$$

which can be rewritten

$$q_i = q_0 \left((1-\delta)e^{-\lambda_1 t_i} + \delta e^{-\lambda_2 t_i} \right)$$

where q_0 is the production at $t = 0$ and δ is the fraction of q_0 getting decayed by the second process.

We assume that the measured production y_i has Gaussian noise of variance σ_i^2. We can therefore write

$$P(y_i|t_i, \sigma_i, q_0, a, \lambda_1, \lambda_2) = \frac{1}{\sigma_i\sqrt{2\pi}} e^{-\frac{1}{2}\left(\frac{y_i - q_i}{\sigma_i}\right)^2}$$

Consequently, the likelihood of the data given the parameters can be expressed as

$$P(y|t, \sigma, q_0, a, \lambda_1, \lambda_2) = \prod_{i=1}^{N} \left(\frac{1}{\sigma_i\sqrt{2\pi}} \right) e^{-\frac{1}{2}\sum_{i=1}^{N}\left(\frac{y_i - q_i}{\sigma_i}\right)^2}$$

where $y = \{y_1, y_2, \cdots y_N\}$, $t = \{t_1, t_2, \cdots t_N\}$, $\sigma = \{\sigma_1, \sigma_2, \cdots \sigma_N\}$, and N is the number of observations.

This likelihood is maximized when the exponential term is minimized, which means that we need to find the set of parameters $\{a, q_0, \lambda_1, \lambda_2\}$ that minimize $\sum_{i=1}^{N}(\frac{y_i - q_i}{\sigma_i})^2$.

It should be noted that the observed production y_i is actually mean of y_{ci} measured for different cells c, that itself have error bars σ_{ci}. We therefore have:

$$\sigma_i = \frac{\sqrt{\sum_{c=1}^{N_{ci}} \sigma_{ci}^2}}{N_{ci}}$$

where N_{ci} is the number of cells we observe at time i.

Assessment of sustained gene expression during prolonged starvation

Because no carbon source is provided in our experiments, bacteria will have to use their reserves to fuel their metabolism. Since the time-dependent volumic production cannot be well-fitted to a mixture of two exponentials for all promoters, we decided to quantify volumic production late in starvation by estimating the average volumic production between 30 and 60 hours of starvation in time windows of 10 hours. Late in starvation the production rates are generally very low and their estimates are affected by fluctuations in autofluorescence and possible single-cell fluctuations in protein degradation rates. Consequently, single-cell production estimates at individual time points becomes extremely noisy. To obtain a meaningful estimate of the production late in starvation we first averaged production over all cells at each time point and then calculated the mean and standard deviation of average production across the time points in each 10 hour bin (Appendix Fig. S10B). We see that, although for a substantial number of promoters that production is barely distinguishable, i.e. with fluctuations in the average production larger than the mean, there is also a substantial number of promoters with consistently positive production up to 60 hours in starvation, e.g. hupA, hslV, pliG, gadX, nrdH, glgS, and sbmC. In general production consistently decreases with time although there are one or two cases, i.e. nrdH and nrdD, where the small rate of production barely decreases with time.

Altogether, this sketches a picture where sustained gene expression during starvation is observed across many promoters, but eventually drops to levels which are too low to be reliably measured.

5 Regulators of synthetic promoters

The synthetic promoters used in this study have been selected to achieve a given expression level during exponential growth (Wolf et al. 2015). We scanned the synthetic promoters for binding sites of transcription factors with known regulatory motifs Arnold et al. (2011); Salgado et al. (2024) and found only binding sites for the sigma factors rpoD and rpoS, suggesting that these promoters are not regulated. Based on this, we selected them to assess how the global gene expression capacity of the cell change upon entry into starvation, in absence of specific regulation. Importantly, it is well established that the two sigma factors RpoD and RpoS recognize very similar motifs. Hence promoters transcribed under the control of one of them will also be transcribed due to the activity of the other, albeit with different intensity (Hengge-Aronis 2002). Given that RpoS is the main sigma factor active during early starvation, it is important to characterize the strength of the regulation by RpoS for our constitutive promoters.

Using ΔrpoS mutants, we were able to demonstrate that the concentration of the two medium expressors (Med2 and Med3) is dramatically decreased in this genetic background compared with the wild type levels, both during growth and starvation (Appendix Fig. S13A). Moreover, the increase of concentration observed for those promoters in the wild type upon switching from exponential growth to starvation is also abolished in the ΔrpoS background (Appendix Fig. S13B). Together, those

observations strongly suggest that the main regulator of the medium expressors is RpoS, meaning that those synthetic promoters can be used as reporters for RpoS activity. On the contrary, the concentration and fold change of the high expressor Hi1 was only mildly affected in the ΔrpoS background compared to the wild type. This implies that Hi1 is primarily regulated by RpoD and we interpret it as a reporter of "unregulated" gene expression.

6 Comparison of microfluidic measurements with transcriptomics

Our microfluidic measurements of transcriptional reporters revealed that, for each promoter, the time-dependent responses across individual cells were sufficiently consistent for the population average to accurately capture the typical time-dependent expression profile of the promoter. Consequently, it should be possible to also capture these time-dependent responses using bulk population measurements such as transcriptomics. We thus decided to compare the time-dependent responses of different promoters with those observed in a transcriptomic dataset. Among several available RNA-seq datasets of *E. coli* during starvation, we focused on data from Lempp et al. (2019) for several reasons. First, the experimental conditions are close to our "hard switch" experiments: *E. coli* K12 (BW25113 instead of MG1655 in our experiments) in M9 minimal media (supplemented with 0.5% glucose instead of 0.2%); starvation is achieved by rapidly washing the culture and re-suspending cells in M9 media without carbon. Second, the sampling scheme is more appropriate for capturing the changes of gene expression taking place upon entry into starvation than in other datasets, i.e. RNA-seq samples were taken at 5, 10, 24, 30, 45 minutes, and then 1, 1.25, 1.5, 1.75, 2, 2.5, 3, 3.5, 4, 5, 6, 8, 10, and 12 hours. Third, the reproducibility between the 2 technical replicates of Lempp et al. (2019) is exceptionally good.

Unfortunately, direct quantitative comparison of our transcriptional reporter data with transcriptomic data is non-trivial. It is inappropriate to directly compare population average fluorescence levels with mRNA levels because these also depend on translation and protein decay rates, which are likely time-dependent. As we argued in the main text, it is reasonable to assume that the volumic production q of a given promoter at a given time-point is proportional to its mRNA level at that time-point. However, a second challenge is that RNA-seq measurements inherently measure *relative* expression, i.e. they do not measure absolute mRNA levels but relative levels of different mRNAs. Comparing relative mRNA levels with the absolute production rates of our transcriptional reporters is further complicated by the fact that we only measured production rates for a subset of promoters.

We thus focused on the set of 12 promoters which were quantified both in our experiments and in the RNA-seq data of Lempp et al. (2019). At each time point, we calculated the *relative* volumic production rates q of each promoter relative to the summed production rate of all 12 promoters. Similarly, we calculated the mRNA levels of the corresponding 12 genes relative to the total mRNA level of all 12 genes at each time point. Notably, this not only turns absolute production rates into relative values across the 12 genes/promoters, but will also correct for time-dependent translation rates that affect the volumic production measurements.

Appendix Figure S9 shows how the relative protein-production and mRNA expression levels vary across time for these 12 promoters. We see that, although there are clear quantitative differences in the observed profiles, for the large majority of promoters the qualitative features are reproduced (e.g. whether a promoter is up-regulated or down-regulated and on what time scale, and to some extent also the size of this response).

We see that some profiles, e.g. bolA, show a very similar time-dependent profile but a systematic shift in the level across the entire time course. This may indicate either a differences in the mRNA stabilities of the 12 native mRNAs, or differences in the capture rates of the 12 native mRNAs in the RNA-seq experiment. For other promoters, the profiles start from similar levels but show a different fold-change, e.g. rplN and sbmC. Such differences could be explained by mRNA stability changing to different extents for the native and reporter mRNAs as conditions change across time. Nonetheless, overall the time-dependent responses show highly varying patterns across promoters that are largely consistent between the transcriptional reporters and transcriptomic measurements.

7 Quantitative proteomics measurements of protein levels

7.1 Sample Preparation

In order to compare the temporal dynamics of the protein levels inferred from the GFP content of the cells and the actual levels of the native proteins produced by our promoter of interest, we used quantitative proteomics. In addition, we compared our results to the fold change between exponential growth and stationary phase conditions found in publicly-available proteomics datasets.

We conducted a low time-resolution experiment with several strains in parallel. Based on the concentration profiles measured in microfluidic experiments (Fig. 4A), we selected reporters covering the range of fold-change observed between exponential growth and starvation (10 out of 18 strains from the Zaslaver library): "pyrB", "codB", "rplN", "pliG", "gadX", "sbmC", "nrdD", "hslV", "hupA", "bolA". We collected 5 mL samples ($\approx 5.10^8$ cells in early exponential phase, at OD600 = 0.1) at 0 h, 1 h, 4 h, 24 h, and 48 h, starting from a 50 mL culture so that half of the initial volume remained at the end of the experiment.

For this, two days before the experiment, we inoculated a 2 mL culture in M9 + 0.2% glucose from individual colonies for each strain. After overnight growth, each culture was resuspended in 10 mL of fresh M9 + 0.2% glucose media at very low density (16000x and 32000x dilution), in order to have cultures still growing exponentially on the next day. For each strain, one culture still growing exponentially ($0.1 \leq$ OD600 ≤ 0.5) was then diluted in a final volume of 50 mL of M9 + 0.02% glucose to an OD of 0.025 (time - 2h). When the culture reached OD600 = 0.1 (0h), we sampled 5 mL of the culture, washed 2 x with 3 mL of ice cold PBS, resuspended the pellet in 3 mL ice cold PBS, centrifuged at 4°C and flash-froze the pellet at -80ºC after carefully removing all supernatant. We repeated this sampling step at every time point.

Proteins were purified and digested with the SP3 approach (Hughes et al. 2019) using a Freedom Evo 100 liquid handling platform (Tecan Group Ltd., Männedorf,

Switzerland). In brief, Speed Beads ™(#45152105050250 and #65152105050250, GE Healthcare) were mixed 1:1, rinsed with water and diluted to the 8 μg/μL stock solution. Samples were adjusted to the final volume of 90 μL and 10 μL of the beads stock solution was added to them. Proteins were bound to the beads by addition of 100 μL of 100% acetonitrile to the samples, which were then incubated for 8 min at RT with a gentle agitation (200 rpm). After, samples were placed on a magnetic rack and incubated for 5 min. Supernatants were removed and discarded. The beads were washed twice with 160 μL of 70 % (v/v) ethanol and once with 160 μL of 100% acetonitrile. Samples were placed off the magnetic rack and 50 μL of digestion mix (10 ng/μL of trypsin in 50 mM triethylammonium bicarbonate) was added to them. Digestion was allowed to proceed for 12 hours at 37°C. After digestion samples were placed back on the magnetic rack and incubated for 5 min. Supernatants containing peptides were collected and dried under vacuum.

7.2 LC-MS analysis

Dried peptides were resuspended in 0.1% aqueous formic acid and subjected to LC–MS/MS analysis using an Orbitrap Eclipse Tribrid Mass Spectrometer fitted with an Ultimate 3000 nano-LC (both Thermo Fisher Scientific) and a custom-made column heater set to 60°C using block randomization. Peptides were resolved using a RP-HPLC column (75 μm × 30 cm) packed in-house with C18 resin (ReproSil-Pur C18–AQ, 1.9 μm resin; Dr. Maisch GmbH) at a flow rate of 0.3 μL/min. The following gradient was used for peptide separation: from 2% B to 12% B over 5 min to 30% B over 40 min to 50% B over 15 min to 95% B over 2 min followed by 11 min at 95% B. Buffer A was 0.1% formic acid in water and buffer B was 80% acetonitrile, 0.1% formic acid in water. The mass spectrometer was operated in DIA mode with a cycle time of 3 seconds. MS1 scans were acquired in the Orbitrap in centroid mode at a resolution of 60,000 FWHM (at 200 m/z), a scan range from 350 to 1200 m/z, normalized AGC target set to 250% and maximum ion injection time mode set to 50 ms. MS2 scans were acquired in the Orbitrap in centroid mode at a resolution of 15,000 FWHM (at 200 m/z), precursor mass range of 400 to 900, quadrupole isolation window of 12 m/z with 1 m/z window overlap, a defined first mass of 120 m/z, normalized AGC target set to 1000% and a maximum injection time of 22 ms. Peptides were fragmented by HCD (Higher-energy collisional dissociation) with collision energy set to 33% and one microscan was acquired for each spectrum.

7.3 Protein Identification and Quantification

To determine changes in protein expressions across samples, a spectral library free DIA analysis was carried out. Therefore, the generated raw files were searched against an *E. coli* database (downloaded from Uniprot on 20220222) and 392 commonly observed contaminants (total of 4764 protein sequences) using DIANN (v1.8.1) (Demichev et al. 2020) and default settings with a few changes: MBR was activated, Protein names were selected for Protein inference and Neural network classifier were set to Double-pass mode. In the additional options box "–report -lib -info" was added. Quantitative results obtained from the report file were further analyzed using the MSstats R package v.4.7.3 (Choi et al. 2014; Kohler et al. 2023). All

mass spectrometry files associated with this manuscript are accessible at MassIVE (ftp://massive.ucsd.edu/v08/MSV000095680/).

7.4 Proteomics fold change calculation from literature

For each proteomics sample s (i.e. time point and replicate) we first log-transform the raw MS signal for each gene g, i.e. $M_{gs} \to \log(M_{gs})$ and normalize by subtracting the average log-signal in the sample, i.e $m_{gs} = \log(M_{gs}) - \langle \log(M_s) \rangle$, with $\langle \log(M_s) \rangle = \sum_g \log(M_{gs})/G$ and G the number of genes. Next, whenever there are replicate measurements of the same condition, the expression m_{gs} is averaged over the replicates. Finally, the log fold-change F_{gt} of gene g at starvation time point t is defined as the difference in log-expression $F_{gt} = m_{gt} - m_{ge}$, where m_{ge} is the log-expression of the gene during exponential growth.

For all datasets, the precise samples that are used and data sources are as follows:

- Data ref: Caglar et al. (2017): Sample names, gene names, dictionary, and protein counts are obtained from the github repository https://github.com/umutcaglar/ecoli_multiple_growth_conditions/tree/master/, downloading the files `Protein_reads_06_16_2016/sampleInformation.csv`, `generateDictionary/nameDictionary_RNA&Protein.csv`, and `Protein_reads_06_16_2016/Protein_Count_GlucoseTimeCourse.csv` respectively. Samples taken in exponential phase at 3, 4 and 5 hours are averaged and used as M_g. Fold changes at 24, 48, 168 and 336h are calculated and represented on Appendix Fig. S11 as samples 1,2,3 and 4, respectively.
- Data ref: Mori et al. (2021): Description of the samples and protein counts are taken from Supplementary Tables EV2 and EV8. The sample named "LB mid log" is used as M_g. Fold changes in late log phase in LB, as well as in stationary phase (following growth in LB) are calculated using the samples named "LB late log", "LB stat 1" and "LB stat 2", and represented on Appendix Fig. S11 as samples 1, 2, and 3 respectively. Note that the two latter samples are repetitions of the experiments, and expected to give the same results.
- Data ref: Schmidt et al. (2016): A table with combined global absolute abundance estimates from both datasets were taken from Supplementary Table S6. The counts in the "Glucose" sample are used as M_g. Fold change after 1 and 3 days are calculated from samples "Stationary phase 1 day" and "Stationary phase 3 days", and represented on Appendix Fig. S11 as samples 1 and 2 respectively.
- Proteomics data from this study: In order to calculate the fold change of native proteins, samples 1 and 2 (Exponential growth) for all 10 strains -that only differ by the plasmid they carry- are averaged and used to calculate m_{ge}. Fold changes after 4h, 24h and 48h starvation are calculated from the 10 replicates of samples 3, 4 and 5 (Starvation 1, 2, 3) and represented on Appendix Fig. S11 as samples 1, 2 and 3 respectively. In order to calculate the fold changes of the GFPmut2 reporters, we proceeded as described above, with the exception that this time, all strains have to be treated as independent experiments -and not as replicates- since the promoter controlling the expression of the protein differs from strain to strain.

7.5 Interpretation of the proteomics measurements

We find that, at least at the level of single proteins, proteomics data are too variable to quantify log fold-changes confidently. Notably, we even observe substantial differences between the fold-changes obtained from our fluorescence measurements in the Mother Machine and proteomics measurements of the concentrations of the corresponding GFPmut2 reporter proteins inside the cells (see Appendix Fig. S11, "Proteo exp. GFP" and "MM exp."). These differences can only be due to technical issues since these measurements measure the same biological quantities. In addition, we observed that for many proteins, proteomics measurements varied erratically between the 24h and 48h measurements, even though all Mother Machine measurements strongly suggest protein concentrations do not substantially change after that first 24h in stationary phase. This too is suggestive of substantial technical variability in the proteomics measurements.

To investigate whether this could be an issue specific to our proteomics measurements, we calculated log fold-changes for the same set of proteins using proteomics data from other labs that is available in the literature. This showed that, rather disturbingly, there is large variation in observed log fold-changes across datasets (Appendix Fig. S11). For some proteins the different datasets do not even agree on whether a given protein is up- or down-regulated in stationary phase relative to its concentration during exponential growth, e.g. hupA, hslV, and nrdD.

In order to obtain an overall comparison of the log fold-changes based on proteomics data of native proteins, proteomics data of the GFP reporters, and fluorescence measurements, we calculated means and standard deviations of the log fold-changes across all measurements, including measurements from different datasets (Appendix Fig. S12). We find that, although there is a lot of variability in log fold-changes across datasets, for the large majority of cases the trend is consistent in that the average log fold-changes are within each other's standard deviations. In particular, the log fold-changes of the GFP concentrations measured by proteomics and the fold-changes measured by fluorescence in the Mother Machine have overlapping mean $\pm$ standard deviation for 10 out of 11 cases (green and blue bars in Appendix Fig. S12). Similarly, for 13 out of 16 cases the log fold-changes of the native protein concentrations measured by proteomics and the fold-changes measured by fluorescence in the Mother Machine have overlapping mean $\pm$ standard deviation (green and red bars in Appendix Fig. S12).

8 A simple mathematical model capturing the diversity of concentration dynamics

The diversity of the observed protein concentration dynamics, can be captured by a simple mathematical model which is characterized by only 3 effective parameters. Moreover, we here show that the fold-change in protein concentration between exponential and late stationary phase can be calculated analytically as a function of these effective parameters.

First, we define $t = 0$ as the time of the switch to starvation and assume that the growth rate drops instantaneously to zero, i.e.

$$\lambda(t) = \begin{cases} \lambda & ; \quad t < 0 \\ 0 & ; \quad t \geq 0 \end{cases} \tag{S22}$$

where λ is the average growth rate before starvation. Note that in this simple model we track the average concentration dynamics as determined by the average growth rate, average protein production, and average protein degradation rate, i.e. we are not modeling single-cell variation.

As already discussed above, the growth rate drops exponentially during stationary phase according to

$$\gamma(t) = \begin{cases} \gamma_0 & ; \quad t < 0 \\ \gamma_0 e^{-\omega_\gamma t} & ; \quad t \geq 0 \end{cases} \tag{S23}$$

where γ_0 is the degradation rate during exponential phase and at entry into starvation, and ω_γ the decay rate of the degradation rate.

Finally, we need to define the protein production rate $q(t)$ as a function of time in stationary phase. Empirically, we have observed that, at the switch, protein production rate either immediately undergoes a sharp drop in production, followed by more slow decay, or shows an initial burst in production, followed by slow decay. Moreover, we have seen that the slow decay in production is often well described by a sum of two exponential decay functions.

We here simplify this observed diversity in production dynamics by assuming that, at the switch, production undergoes an instantaneous jump from q_0 during exponential growth, to bq_0 immediate after the switch. That is, the parameter b denotes the fold change in production upon the switch to starvation. Although our model can be easily extended to have production decay as the sum of two exponentials, we will for simplicity describe the decay of $q(t)$ by a single exponential. That is, the protein production $q(t)$ is described by

$$q(t) = \begin{cases} q_0 & ; \quad t < 0 \\ bq_0 e^{-\omega_q t} & ; \quad t \geq 0 \end{cases} \tag{S24}$$

where w_q is the rate at which production decays.

At the end of the exponential growth phase, the protein concentration c_g will be given by

$$c_g = \frac{q_0}{\lambda}, \tag{S25}$$

where, for simplicity, we have neglected the degradation rate γ_0 since it is so much smaller than the dilution rate λ.

The dynamics of the protein concentration $c(t)$ during stationary phase is described by the differential equation

$$\frac{dc(t)}{dt} = bq_0 e^{-\omega_q t} - \gamma_0 e^{-\omega_\gamma t} c(t). \tag{S26}$$

We are particularly interested in the concentration c_s late in stationary phase, i.e. as $t \to \infty$. Solving the differential equation (S26) we find that c_s is given by the following

integral

$$c_s = c_g e^{-\gamma_0/\omega_\gamma} + bq_0 \int_0^\infty \exp\left[-\omega_q t - \frac{\gamma_0}{\omega_\gamma} e^{-\omega_\gamma t}\right] dt. \tag{S27}$$

This integral can be solved using the substition $x = e^{-\omega_\gamma t}$, which turns the integral into the integral definition of an incomplete gamma-function. Moreover, we are especially interested in the fold-change $f = c_s/c_g$ in protein concentration.

We find for this fold-change

$$f = e^{-\gamma_0/\omega_\gamma} + \frac{b\lambda}{\gamma_0}\left(\frac{\omega_\gamma}{\gamma_0}\right)^{\omega_q/\omega_\gamma - 1}\left[\Gamma\left(\frac{\omega_q}{\omega_\gamma}\right) - \Gamma\left(\frac{\omega_q}{\omega_\gamma}, \frac{\gamma_0}{\omega_\gamma}\right)\right]. \tag{S28}$$

Importantly, the fold change f depends on only 3 effective parameters that correspond to ratios of rates. That is, we define $r_\gamma = \gamma_0/\omega_\gamma$, which controls how much degradation there is overall, $r_q = b\lambda/\gamma_0$ which sets the ratio in fold-changes of production and degration, and $r_t = \omega_q/\omega_\gamma$ which sets the ratio of time scales at which production and degradation decay. In terms of these three ratios we have

$$f = e^{-r_\gamma} + r_q\left(r_\gamma\right)^{1-r_t}\left[\Gamma\left(r_t\right) - \Gamma\left(r_t, r_\gamma\right)\right] \tag{S29}$$

Lower limit on fold-change

To obtain the fold-change f_0 resulting from an instantaneous arrest of production, we just have to set $b = 0$ in equation (S28). We then find

$$f_0 = e^{-r_\gamma} = e^{-\gamma_0/\omega_\gamma}. \tag{S30}$$

Using the degradation rates measured in calibration experiments for the different fluorescent proteins (Appendix Table S5), we obtain lower limits in the range of 44 - 66% (Appendix Table S2).

Fluo	f_0
mCherry	0.48
GFP	0.44
mVenus	0.66

Appendix Table S2. Predicted lower bounds on the fold-change f_0 in concentration for the different fluorescent reporters.

Examples of the diversity in protein dynamics

To give some examples of the concentration dynamics resulting from different time-dependent protein production functions, we use a value of λ corresponding to a doubling time of 1 h^{-1}, and set the initial concentration c_g arbitrarily to 70 FP $\cdot\,\mu\mathrm{m}^{-1}$. The initial degradation rates γ_0 and its decay rate ω_γ are set at 0.03 and 0.05 h^{-1} respectively. q_0 is set at 50 FP $\cdot\,\mu\mathrm{m}^{-1}\cdot\mathrm{h}^{-1}$. The other parameters used for the main text figure are displayed in Appendix Table S3.

Production dynamics	b	ω_q
Instantaneous arrest	0	∞
Gradual decrease (slow)	1	0.1
Gradual decrease (fast)	1	0.2
Burst and gradual decrease (fast)	2	0.2
Reduction and gradual decrease (fast)	0.5	0.2

Appendix Table S3. Parameters of the concentration dynamics model.

Current inlet	Current Media	Duration	Swap	Time of swap
A1	M9 + 0.2% glucose	17h	-	-
B1	M9 Zero ± CAM	5h	A1 → A2	1h
A2	M9 Zero + CAM	15h	B1 → B2	2h
B2	M9 Zero + STRESS	5h	A2 → A3	1h
A3	M9 + 0.2% glucose	30h	-	-

Appendix Table S4. Timing of media switches for the microfluidic experiments with oxidative stress. The current inlet corresponds to the inlet where the highest pressure is applied, and whose media flows through the main channel. Note that in the case of B inlets, the media can be different for the 8 series. Duration corresponds to the total time cells are exposed continuously to the current media. In the example above, the experiment last in total 72 h. The column swap indicates which for which inlets the media reservoir(s) are swapped. The time of swap is indicated relative to the start of the current media.

Fluo	$\gamma_0(\mathrm{h}^{-1})$	$\omega(\mathrm{h}^{-1})$
mCherry	0.0215	0.029
GFP	0.028	0.034
mVenus	0.0215	0.052

Appendix Table S5. Parameters of the time-dependent protein degradation rates. The degradation rate $\gamma(t)$ is fitted to $\gamma_0 e^{-\omega t}$ in control experiments with IPTG-inducible promoters.

Experiment	Strain	#Growth cell	#Starv. cell	#Transition cell	#Replicates
Hardswitch	Med2 bolA	3745	496	307	4
Hardswitch	Med2 bolA ΔrpoS	617	79	50	1
Hardswitch	Med3	3912	457	269	3
Hardswitch	Med3 Pl.	3851	510	2977	3
Hardswitch	Med3 bolA	5434	754	455	5
Hardswitch	Med3 bolA ΔrpoS	536	73	45	1
Hardswitch	Hi1	1419	178	101	1
Hardswitch	Hi1 ΔrpoS	1339	149	103	1
Hardswitch	Hi2 bolA	7287	974	592	5
Hardswitch	gshA	790	106	63	1
Hardswitch	yrdC	710	92	66	1
Hardswitch	pyrB Pl.	3068	383	222	2
Hardswitch	argC Pl.	2862	325	208	2
Hardswitch	codB Pl.	2895	284	216	2
Hardswitch	codB Pl. ΔrpoS	1335	144	96	1
Hardswitch	rpmB	1934	230	137	2
Hardswitch	rpsB	707	82	57	1
Hardswitch	rpsB ΔrpoS	2489	284	189	2
Hardswitch	rplN	2244	314	195	2
Hardswitch	rplN ΔrpoS	376	61	28	1
Hardswitch	rplN Pl.	1027	131	85	1
Hardswitch	rplN Pl. ΔrpoS	2025	330	173	1
Hardswitch	rrnB	726	100	57	1
Hardswitch	rrnB ΔrpoS	723	92	64	2
Hardswitch	hupA Pl.	3355	413	225	2
Hardswitch	hupA Pl. ΔrpoS	1786	178	129	1
Hardswitch	hslV Pl.	3582	468	254	2
Hardswitch	hslV Pl. ΔrpoS	370	131	31	1
Hardswitch	bolA Pl.	2072	237	156	2
Hardswitch	pliG Pl.	1928	259	132	2
Hardswitch	gadX Pl.	3506	512	255	2
Hardswitch	gadX Pl. ΔrpoS	1024	88	71	1
Hardswitch	nrdH Pl.	3278	459	233	3
Hardswitch	nrdD Pl.	1839	272	136	2
Hardswitch	glgS Pl.	2248	289	156	2
Hardswitch	glgS Pl. ΔrpoS	1606	197	133	1
Hardswitch	sbmC Pl.	2480	330	175	2
Overflow	Med2 bolA	364	51	27	1
Overflow	Med3 bolA	486	77	38	1
Overflow	Hi2 bolA	2325	387	209	2
Overflow	rpmB	1112	204	97	1
Overflow	rplN	1147	207	104	1
Overflow	rrnB	1068	197	100	1
Stress: Unperturbed	Med3 bolA	2540	657	203	2
Stress: CAM after burst	Med3 bolA	7197	492	284	2
Stress: CAM thr. starv.	Med3 bolA	9769	623	289	2
Stress: Unperturbed	Med3 bolA ΔrpoS	2628	1044	256	2
Degradation measurement	pTrc99a-GFPmut2	NA	121	NA	1
Degradation measurement	pTrc99a-mCherry2-L	NA	29	NA	1
Degradation measurement	pTrc99a-mVenus	NA	207	NA	2
Photobleaching measurement	pTrc99a-GFPmut2	369	83	23	1
Photobleaching measurement	pTrc99a-mCherry2-L	361	48	32	1
Photobleaching measurement	pTrc99a-mVenus	775	283	95	3

Appendix Table S6. Summary of cell numbers in the different experiments. Transition cells correspond to cells undergoing the switch from M9+0.2% glucose to M9 zero.

Appendix References

[DATASET] Caglar MU, Houser JR, Barnhart CS, Boutz DR, Carroll SM, Dasgupta A, Lenoir WF, Smith BL, Sridhara V, Sydykova DK, et al (2017) The *E. coli* molecular phenotype under different growth conditions. Scientific Reports 7:45303

Choi M, Chang CY, Clough T, Broudy D, Killeen T, MacLean B, Vitek O (2014) MSstats: an R package for statistical analysis of quantitative mass spectrometry-based proteomic experiments. Bioinformatics 30(17):2524–2526

Demichev V, Messner CB, Vernardis SI, Lilley KS, Ralser M (2020) DIA-NN: neural networks and interference correction enable deep proteome coverage in high throughput. Nat Methods 17(1):41–44

Hengge-Aronis R (2002) Stationary phase gene regulation: what makes an *Escherichia coli* promoter sigmas-selective? Current Opinion in Microbiology 5(6):591–595

Hughes CS, Moggridge S, Müller T, Sorensen PH, Morin GB, Krijgsveld J (2019) Single-pot, solid-phase-enhanced sample preparation for proteomics experiments. Nat Protoc 14(1):68–85

Kohler D, Staniak M, Tsai TH, Huang T, Shulman N, Bernhardt OM, MacLean BX, Nesvizhskii AI, Reiter L, Sabido E, et al (2023) MSstats version 4.0: Statistical analyses of quantitative mass spectrometry-based proteomic experiments with chromatography-based quantification at scale. J Proteome Res 22(5):1466–1482

[DATASET] Mori M, Zhang Z, Banaei-Esfahani A, Lalanne JB, Okano H, Collins BC, Schmidt A, Schubert OT, Lee DS, Li GW, et al (2021) From coarse to fine: the absolute *Escherichia coli* proteome under diverse growth conditions. Molecular Systems Biology 17(5):e9536

[DATASET] Schmidt A, Kochanowski K, Vedelaar S, Ahrné E, Volkmer B, Callipo L, Knoops K, Bauer M, Aebersold R, Heinemann M (2016) The quantitative and condition-dependent *Escherichia coli* proteome. Nature Biotechnology 34(1):104–110

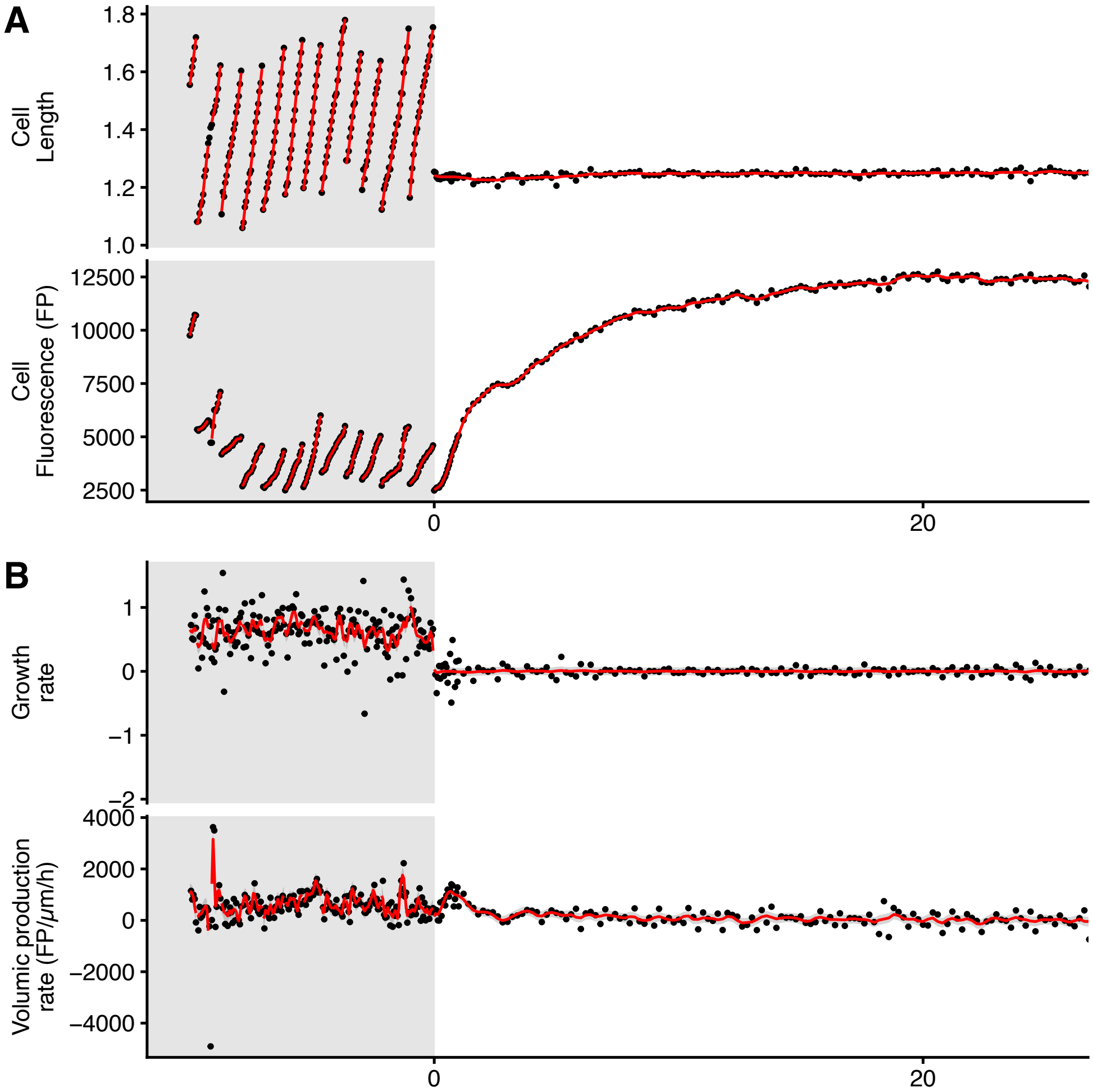

Appendix Figure S1. Inference of instantaneous growth rate and production rate from noisy traces of cell length and fluorescence. RealTrace, a Bayesian procedure based on Gaussian prior process assuming that growth rate and production rate fluctuate as OU processes, was used to predict their best estimate at each time point (Kscheschinski et al. 2025). (A) Raw length and fluorescence measurements (black dots) along with their inferred ”true” values (red lines) from a randomly picked mother cell lineage. (B) Instantaneous derivatives calculation of the corresponding growth and production rates (black dots) and the corresponding inferred rates (red line). In A and B the grey ribbons surrounding the red line show the errors predicted by the inference.

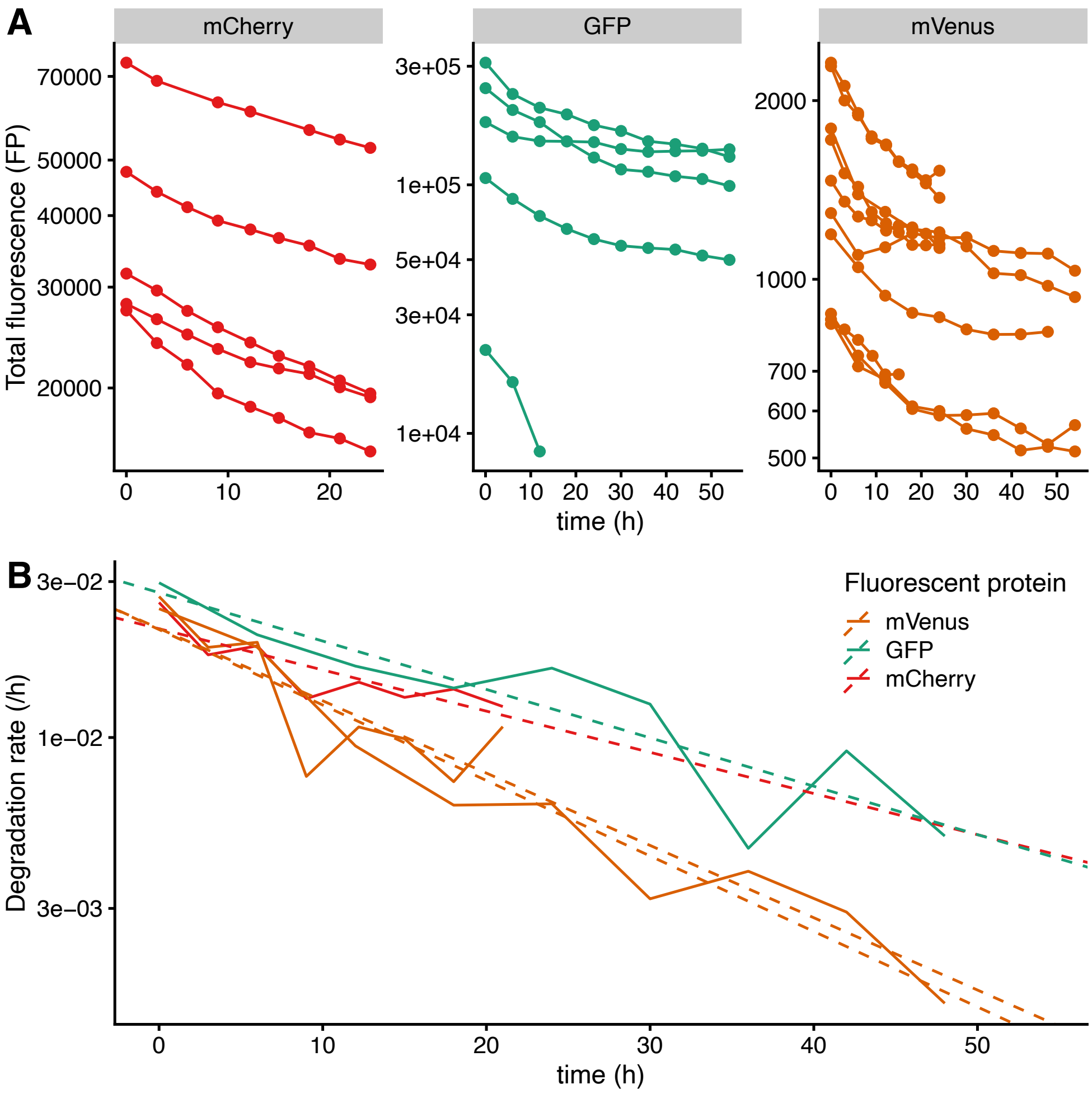

Appendix Figure S2. Estimation of the degradation rates of fluorescent proteins. Independent calibration experiments are performed with low acquisition frequency in order to ensure that photobleaching is negligible. (A) Random selection of traces of raw total fluorescence along time measured for starved bacteria carrying an inducible promoter, for which the inducer has been removed at t=0 (entry into starvation). The acquisition frequency was every 3 hours for mCherry2 and one mVenus replicate, and every 6 hours for the others. (B) Average instantaneous exponential degradation rate, defined as $\langle \frac{1}{F_c(t)} \frac{dF_c(t)}{dt} \rangle$ where $F_c(t)$ is the total fluorescence in cell c at time t. The dashed line corresponds to an exponential fit of this value as a function of time. Pearson correlation R^2 values of the fit are between 0.7 and 0.95. The initial values of the degradation rates at time zero ($0.03h^{-1}$) are comparable to the rates reported at vanishing growth (Calabrese et al. 2022).

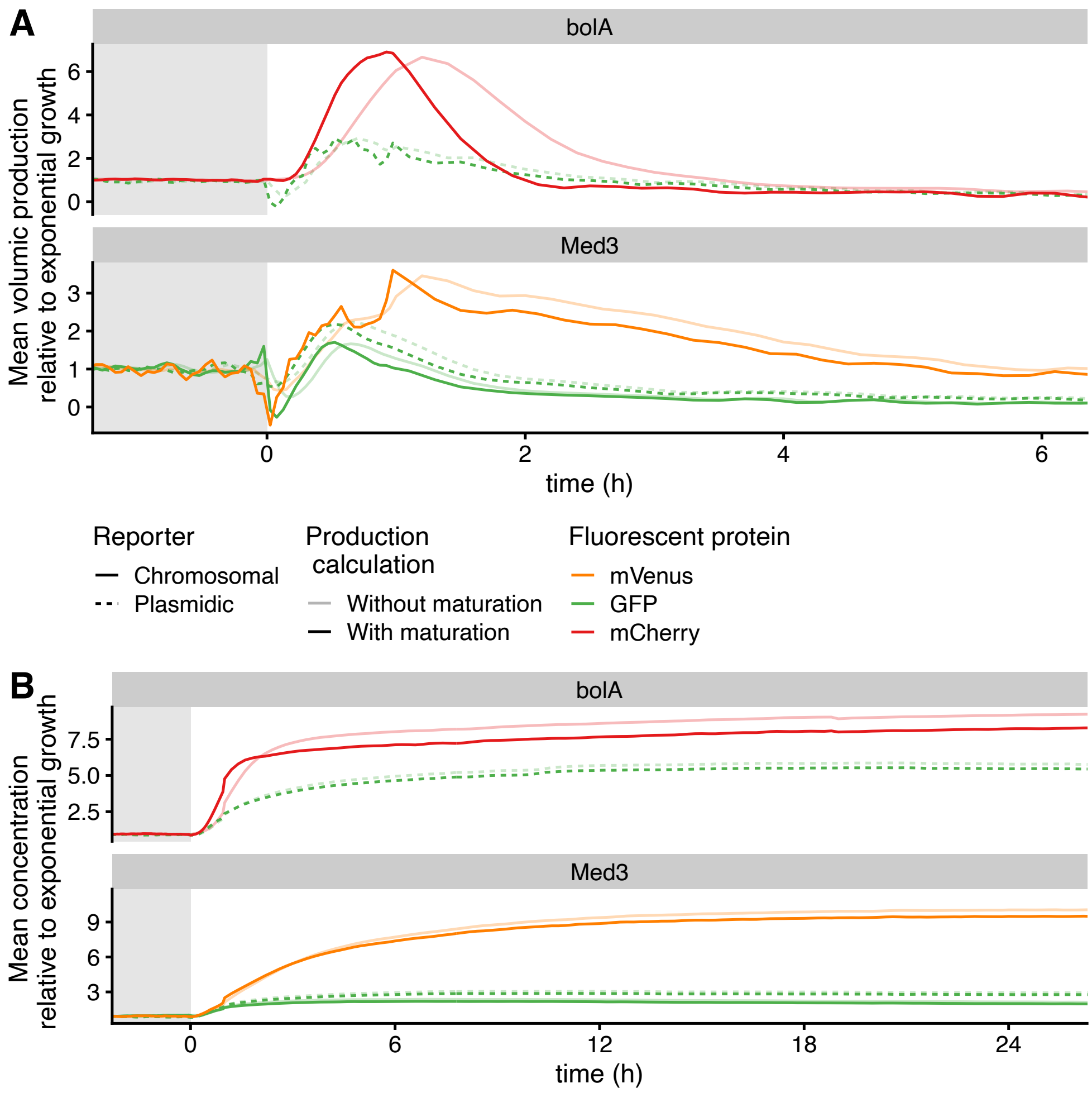

Appendix Figure S3. Comparison of fluorescent reporters and maturation dynamics. (A) Mean volumic production across time for two promoters (bolA and Med3) controlling the expression of different fluorescent proteins (colors: mCherry2-L, mVenus, GFPmut2) and/or with different reporter insertions (line type: plasmidic or chromosomal). The lines show the mean across all single cells normalized by the average value during exponential growth. The opaque lines correspond to the volumic production calculated by taking in account maturation time of the fluorescent protein (see Appendix Section 3), while the transparent lines do not take maturation time into account. (B) Mean fluorescence concentration across time for two promoters (bolA and Med3) controlling the expression of different fluorescent proteins (colors: mCherry2-L, mVenus, GFPmut2) and/or with different reporter insertions (line type: plasmidic or chromosomal). The lines show the mean across all single cells normalized by the average value during exponential growth. The opaque lines correspond to the volumic production calculated by taking in account maturation time of the fluorescent protein (see Appendix Section 3), while the transparent lines do not take maturation time into account. See the Reagents and Tools Table and Appendix Table S6 for more details on the strains used in the different experiments.

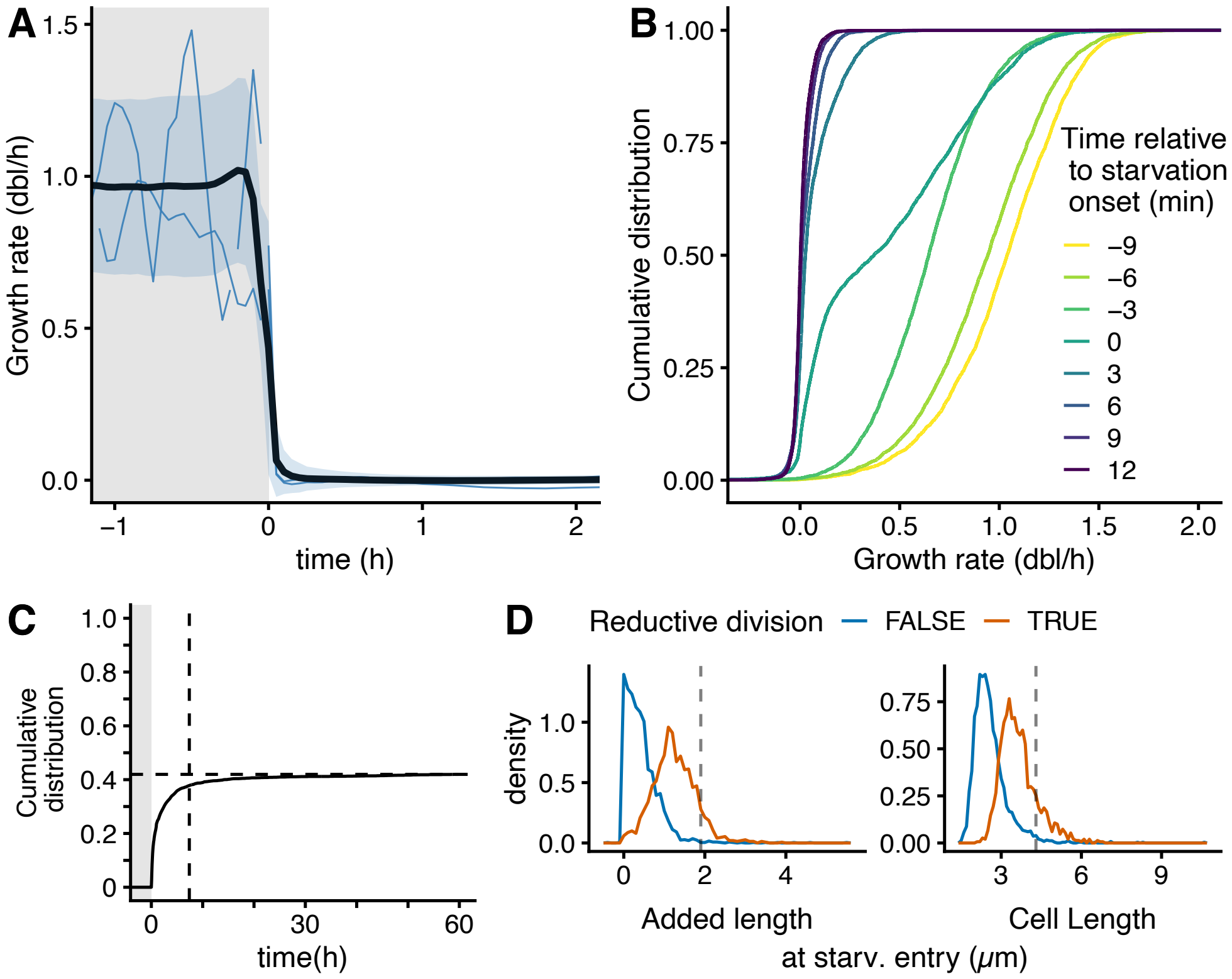

Appendix Figure S4. Growth dynamics upon entry into starvation. (A) After switching to "M9 zero" (defined as time 0), elongation stops almost instantaneously. The black line and ribbon show mean and mean ± s.d. of the instantaneous growth rate across all single cells, respectively; blue lines show randomly picked single cell traces. (B) Cumulative distribution of instantaneous growth rates across cells at each time point around the switch. All cells stop growing within 6 min. (C) Cumulative distribution of division across time, relative to the number of cells entering starvation. The horizontal dashed line corresponds to the final fraction of cells that divided (42.6%), and the vertical dashed line indicate the moment where 90% of the divisions have happened. (D) Distribution of added length since last division and of cell length, measured at the point of starvation entry. Colors indicate whether cells undergo a division during the first 10 hours of starvation (called "reductive division").

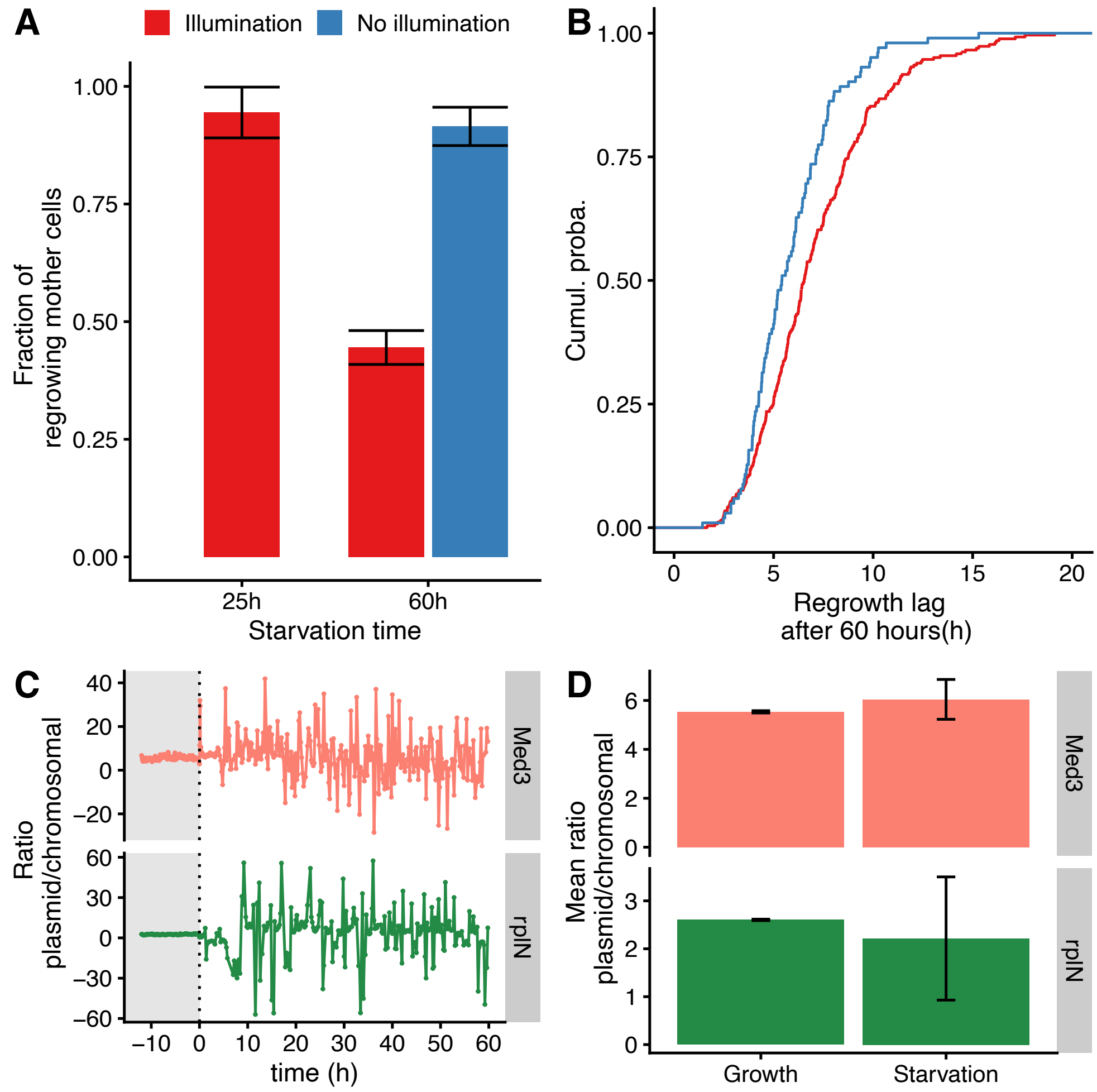

Appendix Figure S5. Experimental setup controls for the hard switch experiments. (A) To assess the phototoxicity of our fluorescence illumination protocol, we measured the fraction of cells surviving after 25 h or 60 h in starvation, with or without illumination. Survival is assessed for "mother cells" (at the closed tip of the Mother Machine growth channels) as the ability to regrow in fresh media within 20 h after the end of starvation. (B) Cumulative distribution of lag of regrowth for regrowing cells. The lag is estimated as the delay between the switch and the point when the exponential fit on the last 5 time points of a cell cycle crosses the mean length at the exit of starvation. (C) Plasmid copy number is inferred by comparing the ratio of average production q of the plasmidic reporter and that of the chromosomal reporters for 2 promoters. During starvation, the promoter activity approaches 0 and becomes noisier, which explains the increase of the fluctuations in ratio amplitude. (D) Average of volumic production ratio between plasmidic and chromosomal reporters, during growth and starvation. Because the ratio can be very noisy during starvation, certain extreme points are driving the mean to artifactually high/low values. Those statistics have therefore been computed after removing the 2.5% highest and lowest ratio values. The difference of ratio between promoter is of unknown origin, and, even though it does not affect the changes of activity observed from growth to starvation, calls for caution when comparing absolute production levels between promoters.

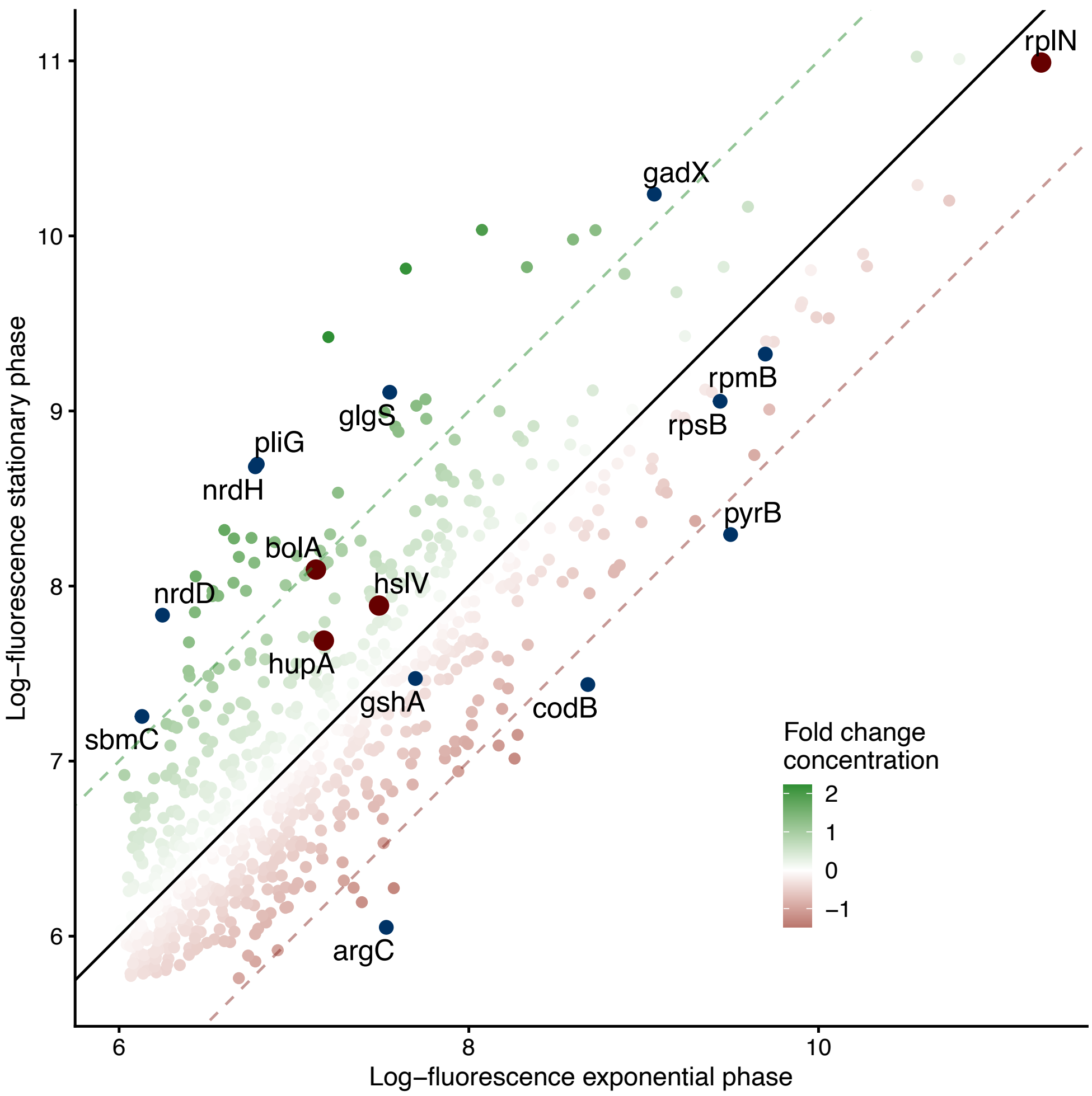

Appendix Figure S6. Selection of native promoters. Log of mean fluorescence measured in stationary phase cultures (after 2 days) as function of that measured in exponentially growing cultures (data from Urchueguía et al. (2021)). Every point corresponds to a promoter from the Zaslaver library (Zaslaver et al. 2006), with the fold change of concentration between the two conditions indicated in color. Promoters are chosen so as to cover a broad range of fluorescence fold change and absolute levels (dark points; dark red points indicate promoters used as examples in the main figures).

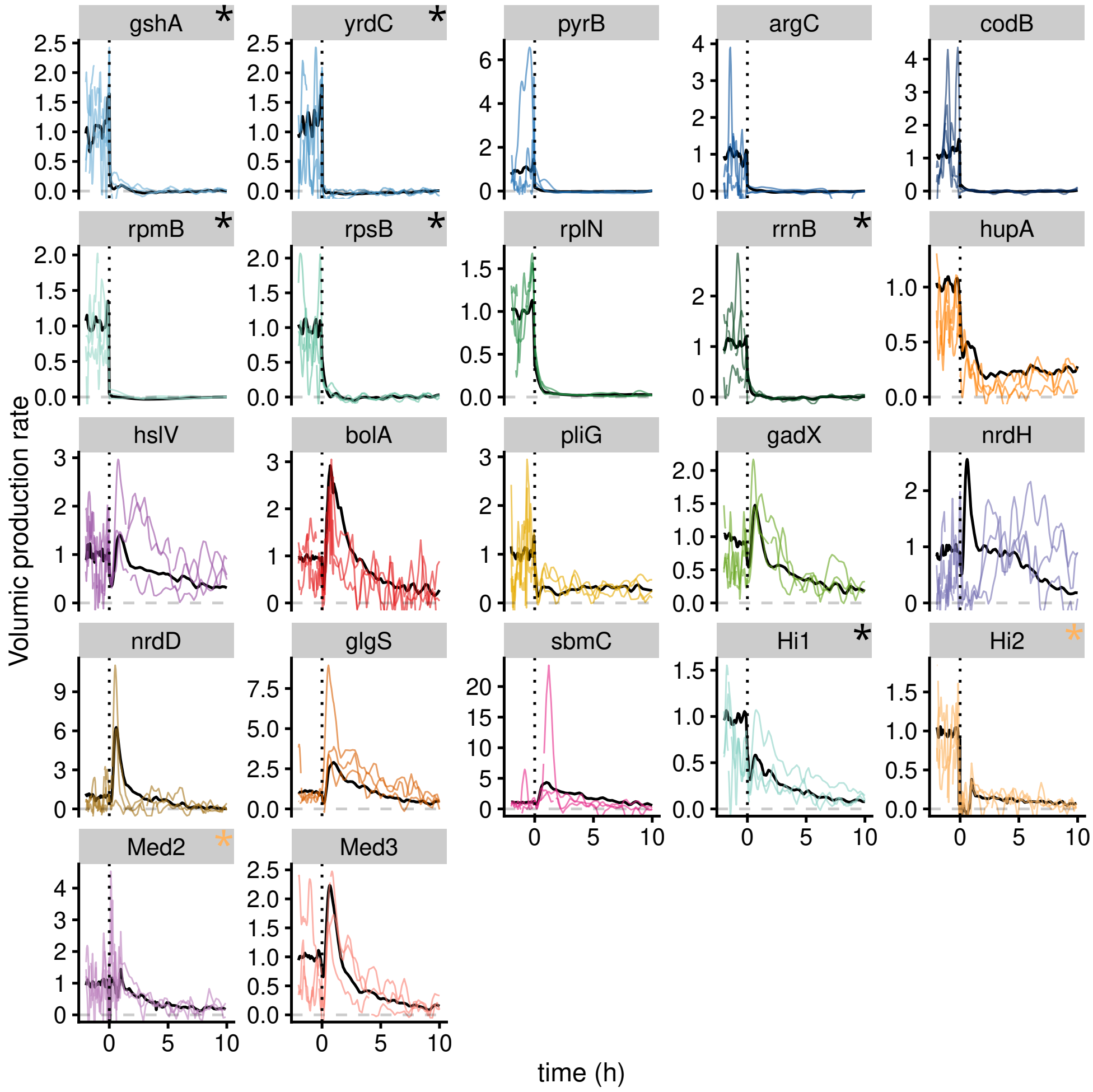

Appendix Figure S7. Production dynamics of individual cells for all promoters at the onset of starvation. Each panel shows example single-cell traces of volumic production q for one of the promoters used in this study (indicated at the top) with the dark line showing the time-dependent volumic production rate averaged over the population of single cells. We see that, although there is significant single-cell variability, the general time-dependent profile of each promoter is reproduced by each single cell. Unless otherwise indicated the fluorescent reporter is GFPmut2 transcribed from a low-copy number plasmid. Chromosomal promoters are indicated by an asterisk next to the name, with red and yellow asterisks indicating mCherry and mVenus, respectively.

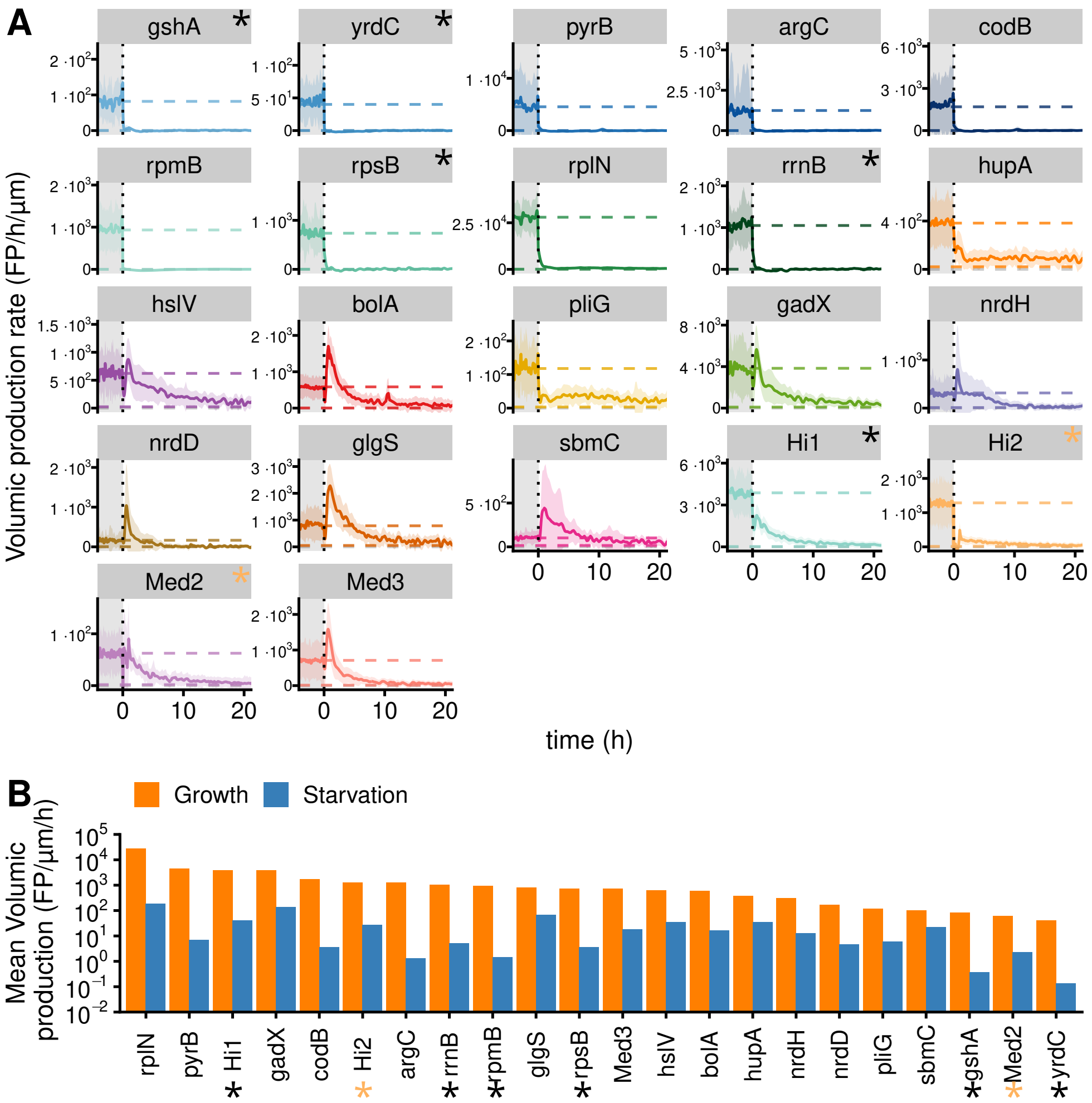

Appendix Figure S8. Production dynamics of all promoters at the onset of starvation. (A) Mean volumic production rate across time (only the first 20 hours of starvation are shown out of 60 hours). Plain lines correspond to average across all individual cells while ribbons correspond to mean ± s.d. The average of volumic production during exponential growth is represented as the upper dashed line. Average volumic production for the last 30h of starvation is represented as the lower dashed line. (B) Average volumic production is computed during exponential growth, and during the last 30h of starvation. Note that the production does not always reach a steady state during the last 30h of starvation (see Appendix Fig. S10). Unless otherwise indicated the fluorescent reporter is GFPmut2 transcribed from a low-copy number plasmid. Chromosomal promoters are indicated by an asterisk next to the name, with red and yellow asterisks indicating mCherry and mVenus, respectively.

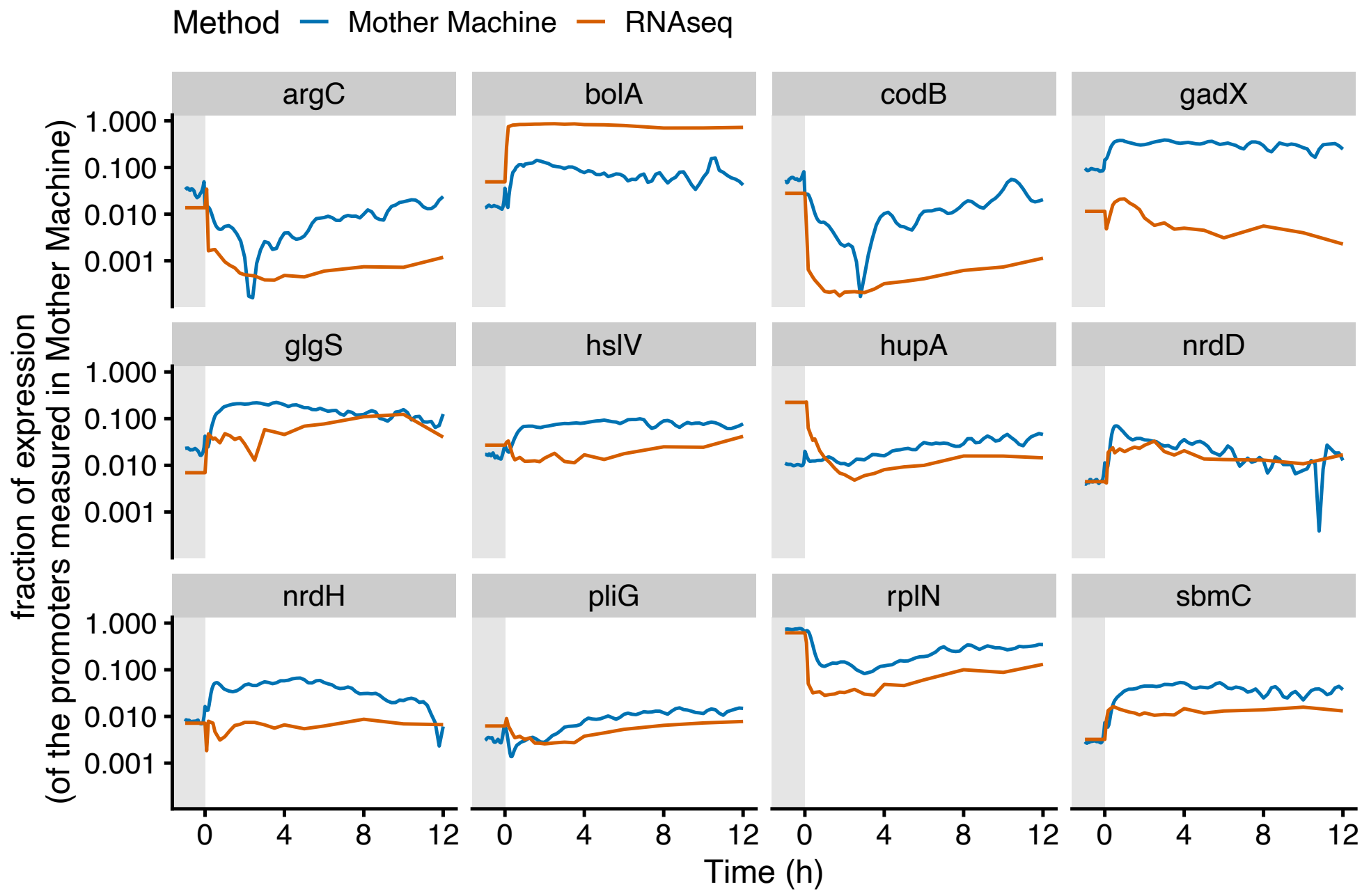

Appendix Figure S9. Comparison with the relative expression levels measured by transcriptomics. The relative expression levels of 12 promoters/genes were quantified using both fluorescent transcription reporters (our experiments) and transcriptomics (data from Lempp et al. (2019)). Each panel corresponds to one promoter/gene and shows the time-dependent volumic production $q(t)$ relative to the volumic production of all 12 promoters/genes (blue) and the mRNA levels relative to the summed mRNA level of all 12 promoters/genes (red). Note that the grey section of the time course corresponds to the exponential growth phase and that the vertical axes are shown on a logarithmic scale. For more details, see Appendix Section 6.

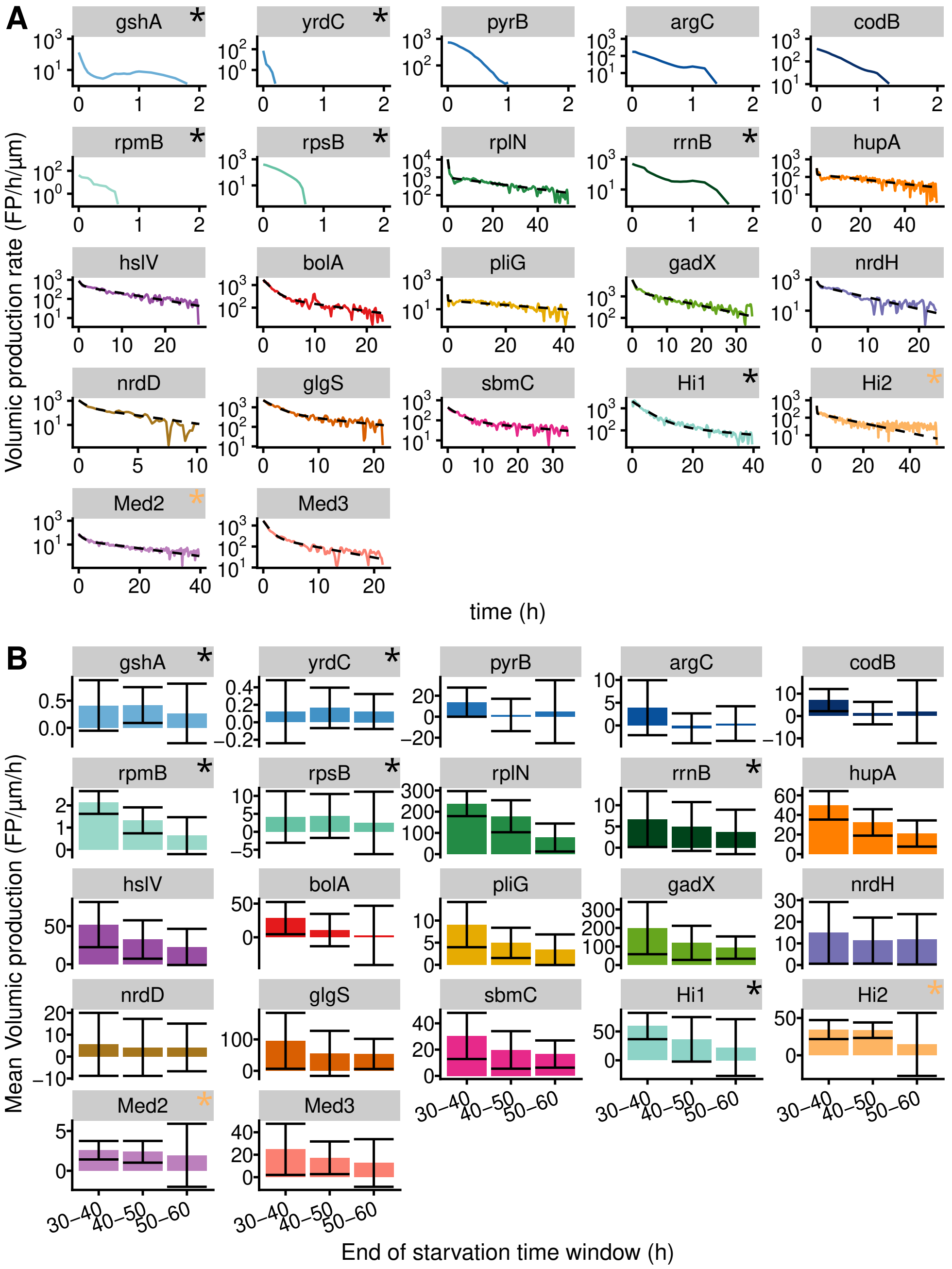

Appendix Figure S10. The late dynamics of volumic production is promoter dependent. (A) Average volumic production rates as a function of time starting from the time where production rate is maximal (defined as time 0) until the first time point where it fluctuates below 0. Solid colored lines correspond to the average of volumic protein production across the population, and the black dashed lines correspond to fits to a sum of two exponentials. Vertical axes are shown on a logarithmic scale. Only promoters with more than 30 observations are shown. (B) Average volumic production rate over three 10-hour segments of the last 30 hours of starvation (whiskers correspond to ± standard deviation of the population average production over the time points within the 10 hour segment). Unless otherwise indicated the fluorescent reporter is GFPmut2 transcribed from a low-copy number plasmid. Chromosomal promoters are indicated by an asterisk next to the name, with red and yellow asterisks indicating mCherry and mVenus, respectively. For more details, see Appendix Section 4.

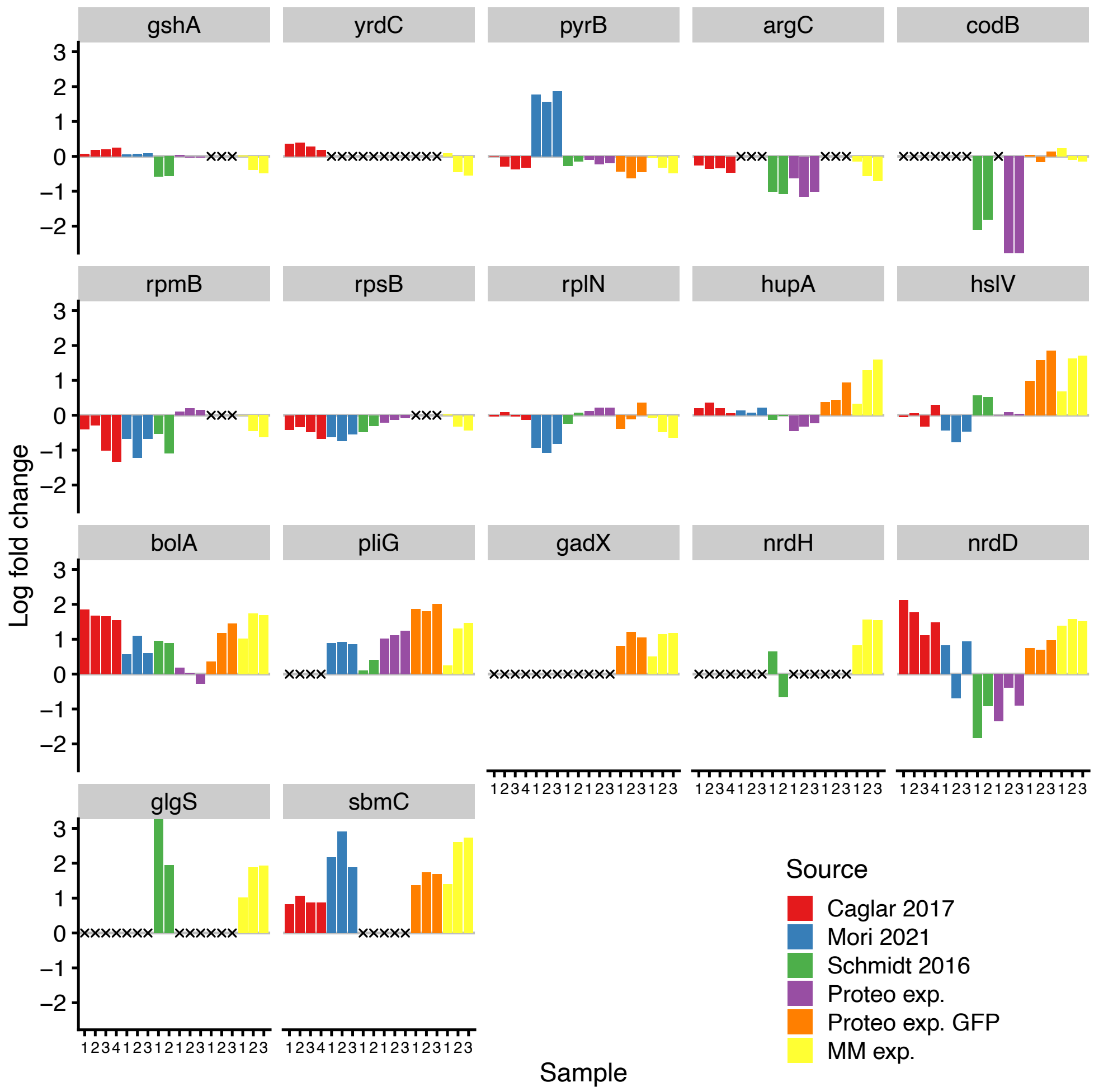

Appendix Figure S11. Analysis of protein concentration changes between growth and starvation using quantitative proteomics. In order to investigate the effects of protein-specific degradation on concentration levels, we performed quantitative proteomics experiments and compared our results with the results from publicly available proteomics datasets obtained under similar conditions, i.e. Schmidt et al. (2016); Caglar et al. (2017), and Mori et al. (2021). Since our experiments were performed with strains from the Zaslaver library (Zaslaver et al. 2006), we could track levels of GFP from different transcription reporters (Proteo exp. GFP) as well as the levels of the native proteins (Proteo exp.). In addition, we report the fold change observed in the Mother Machine experiments when using those reporters (MM exp.). Each panel corresponds to a gene and the bars show fold change of normalized mass spectrometry signal between exponential growth conditions and their corresponding starvation conditions. When multiple measures have been made in starvation conditions, we report them with increasing sample number (x axis). Note that some proteins were not quantified in some datasets due to technical issues. Such missing data are indicated by an "x". For more details, see Appendix Section 7.

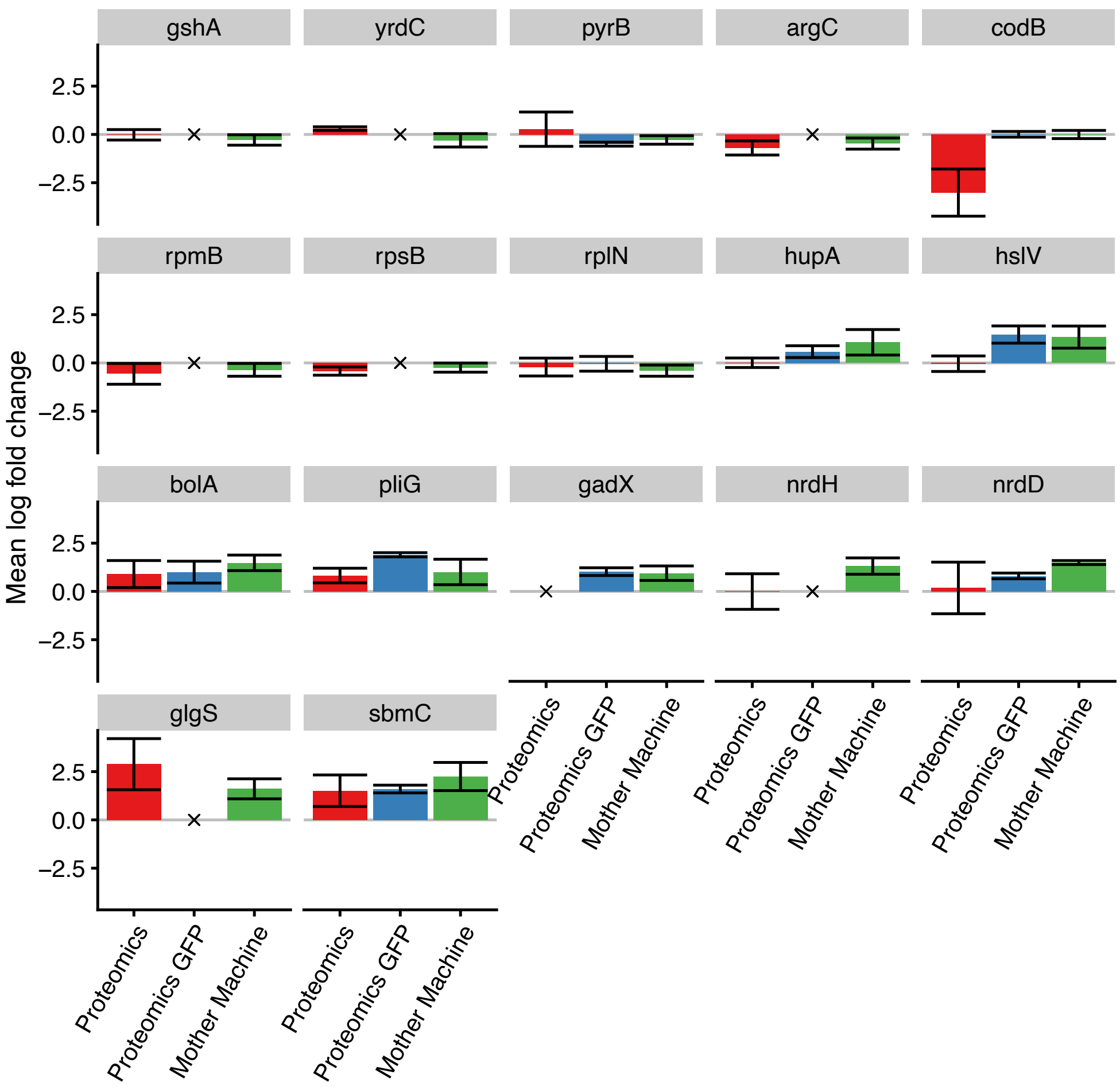

Appendix Figure S12. Analysis of the overall agreement in expression fold-changes between growth and starvation as measured by proteomics and fluorescent reporters. In order to estimate the general tendency of protein fold changes between exponential growth and starvation, we pooled the publicly available proteomics results with our own proteomics results on native proteins ("Proteomics"), and compared them with our proteomics results on GFP from different transcriptional reporters ("Proteomics GFP") and with the fold change calculated from our mother-machine experiments ("Mother Machine"). Each panel corresponds to a gene and shows the mean log fold change and its standard deviation across the samples defined on Appendix Fig. S11. Proteins that were not quantified by a given method are indicated with an "x". For more details, see Appendix Section 7.

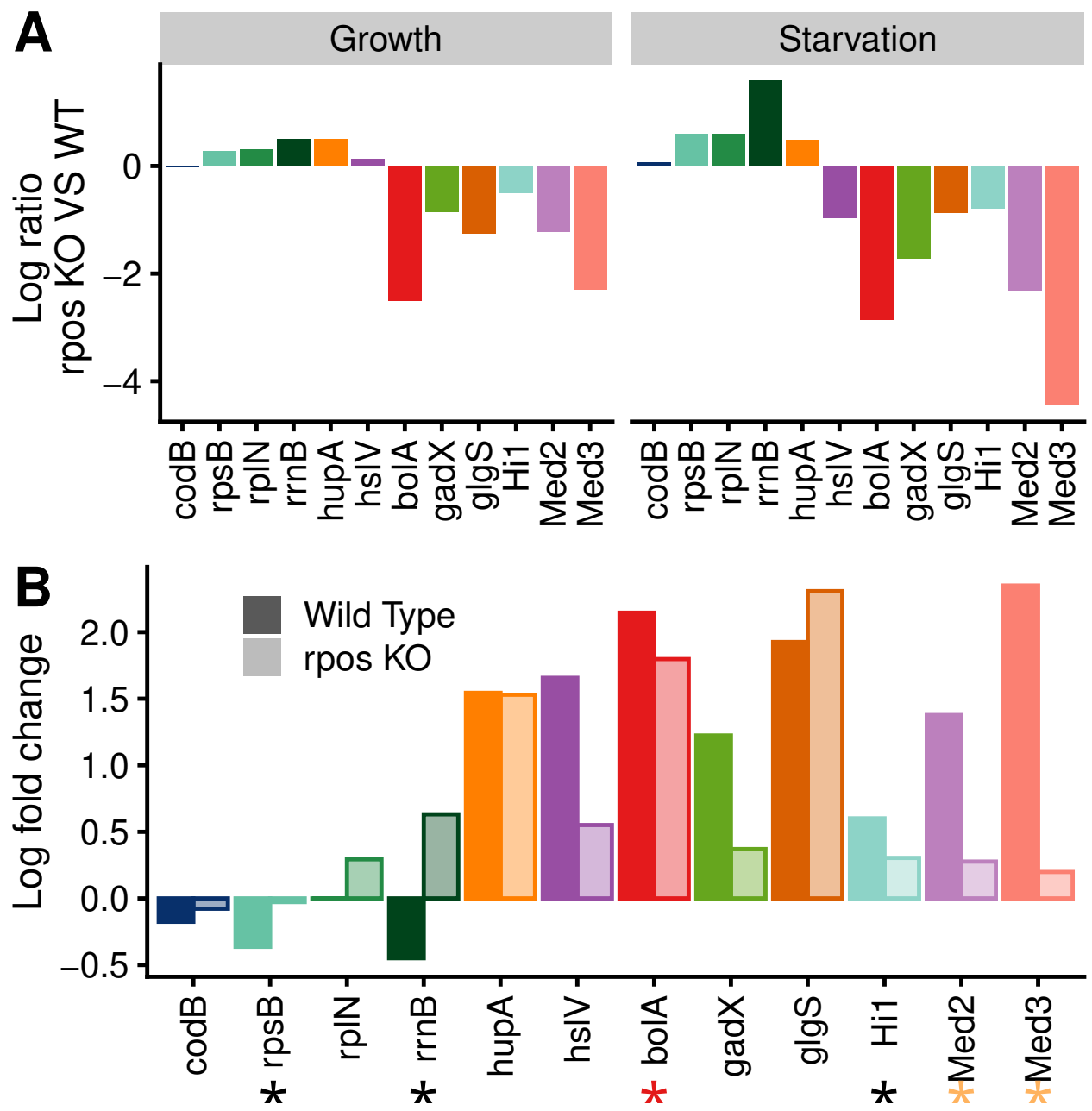

Appendix Figure S13. Expression levels of a selection of reporters in the ΔrpoS mutant. (A) Each bar shows the natural log-ratio of fluorescence concentration during the last 5 hours of growth (left) or starvation (right) for the ΔrpoS mutant *versus* the wild type background. (B) Log fold-changes of fluorescence concentration from exponential growth to starvation for the ΔrpoS mutant (semi-transparent bars) and the wild type background (solid bars). Unless otherwise indicated the fluorescent reporter is GFPmut2 transcribed from a low-copy number plasmid. Chromosomal promoters are indicated by an asterisk next to the name, with red and yellow asterisks indicating mCherry and mVenus, respectively.

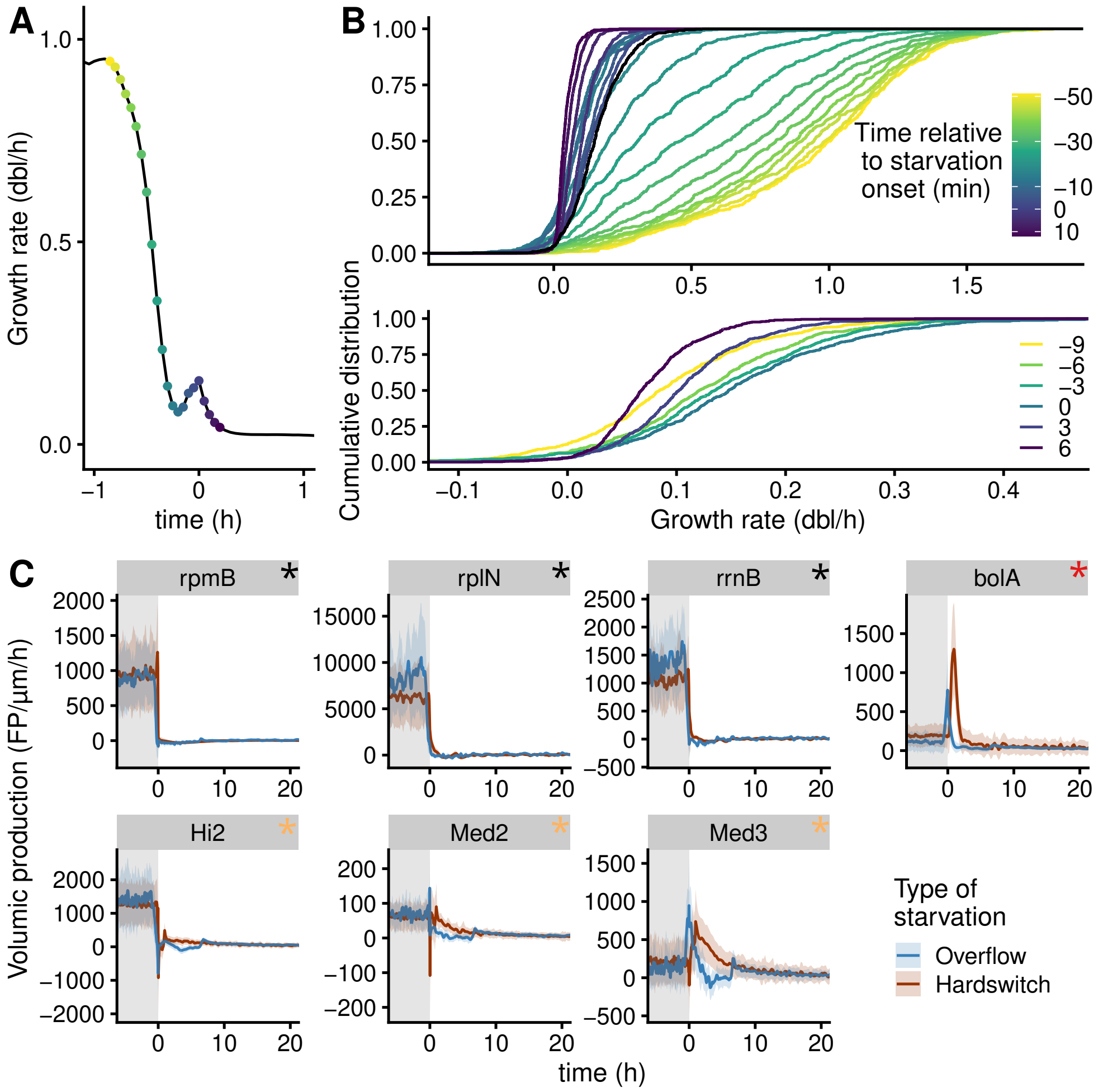

Appendix Figure S14. Comparison of a hard switch with a gradual entry into stationary phase. (A) The average instantaneous exponential growth rate of cells in the chip as a function of time in the overflow experiments. Individual time points during the sharp decrease in average growth rate, at 3 min resolution, are indicated with different colored dots. The color gradient is aligned with time. (B) Cumulative distribution of instantaneous growth rates across cells at each time point from panel A. Growth rates start decreasing 45 minutes before time zero, and by 10 minutes after time zero, essentially all cells have stopped growing (top panel). A zoom into a 15-minute window around time 0 shows a transient increase in growth rate and then a decrease to almost 0 for all cells (bottom panel). (C) Average volumic production rate versus time for every promoter that has been measured in the two microfluidic setups (indicated in colours). Only the first 20 hours are shown although the experiments lasted between 60 hours and 100 hours. Note that the precise temporal alignment of the profiles depends on the choice of time zero for the gradual switch. Chromosomal promoters are indicated by an asterisk next to the name, with black, red and yellow asterisks indicating GFPmut2, mCherry and mVenus, respectively.

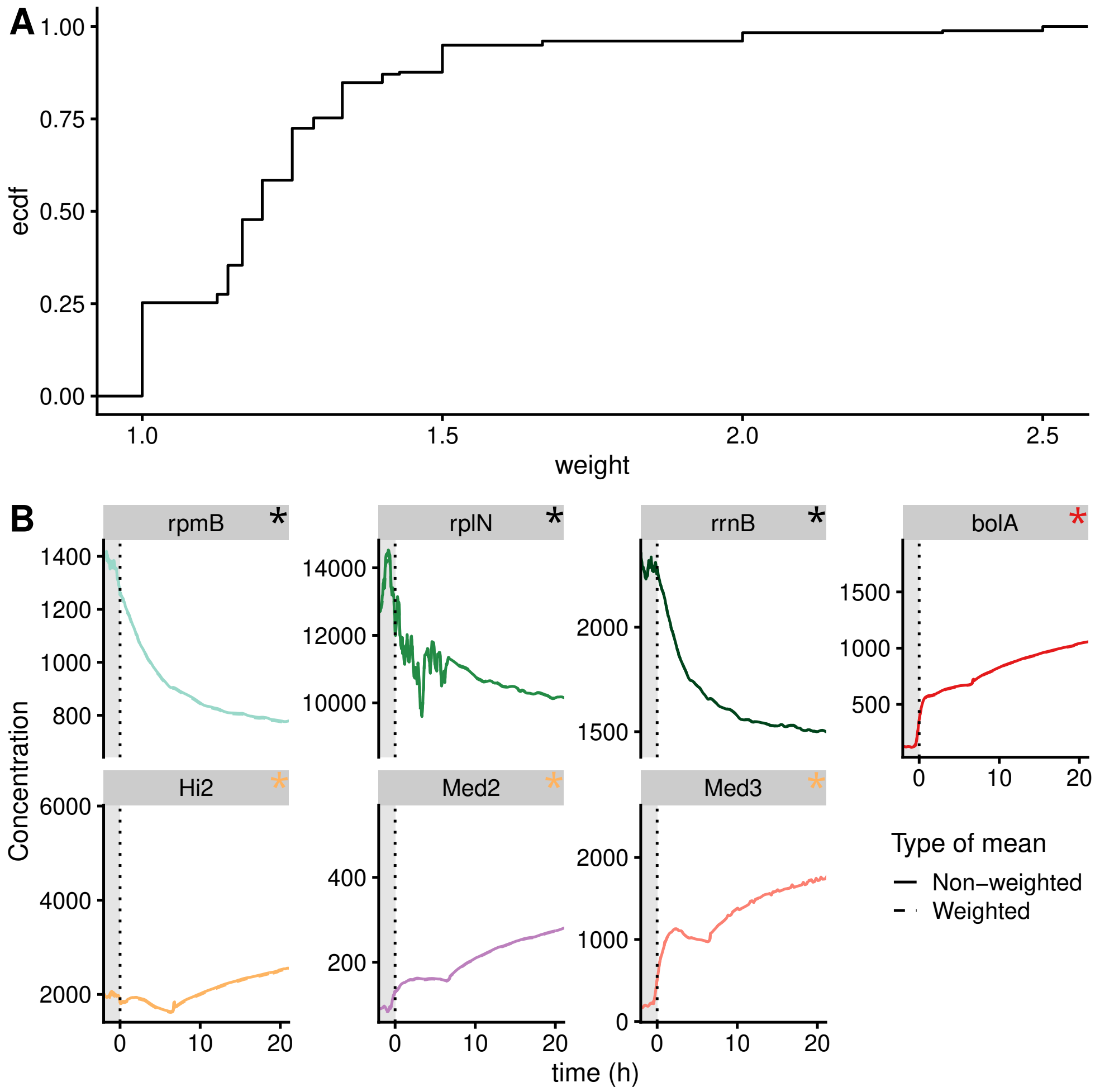

Appendix Figure S15. Controlling for population sampling bias in the Mother Machine during overflow experiments. (A) Distribution of weights over all individual cells. Since growing cells and their progeny are eventually exiting the Mother Machine channels, non-growing cells are overrepresented in Mother Machine experiments compared to batch culture experiment. To check how much this biases our observations on gene expression dynamics, we computed, for each channel, the weight $(n_s + n_t)/n_s$, where n_s is the number of cells present at the entry into starvation and n_t the number of cells pushed out of the channel during the growth rate transition phase (from -1 hour to +1 hour, see Methods). (B) Comparison of average concentration traces with and without the weights defined above for the promoters that were measured in the overflow experiments. Note that weighted and non-weighted averages are almost the same so that the curves are almost indistinguishable. Chromosomal promoters are indicated by an asterisk next to the name, with black, red and yellow asterisks indicating GFPmut2, mCherry and mVenus, respectively.

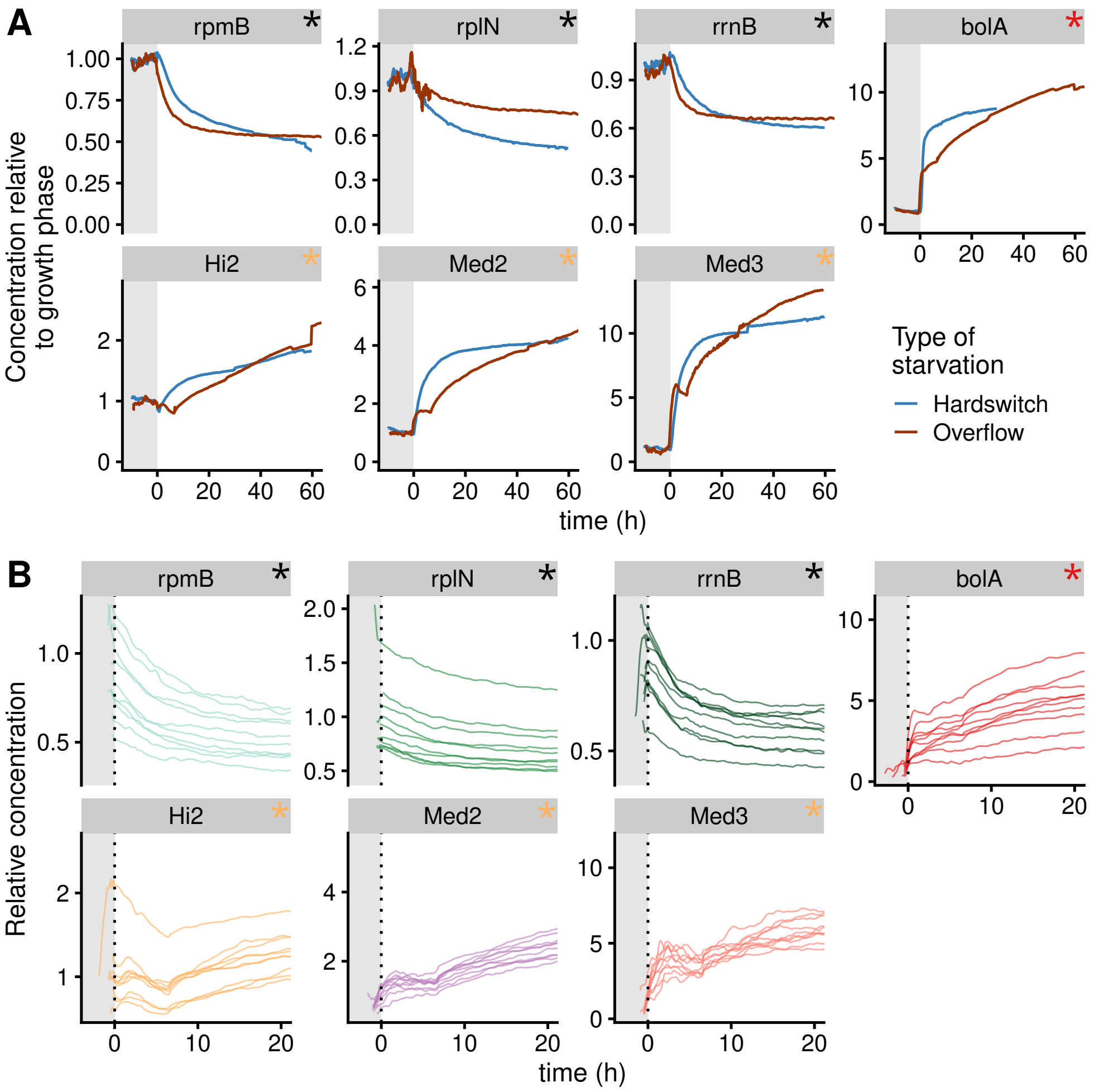

Appendix Figure S16. Comparison of protein concentration dynamics for the hard switch and gradual entry into stationary phase. (A) Average concentration relative to the average concentration during exponential growth as a function of time for every promoter that was measured for both the hard switch and overflow experiments (indicated in colors), calculated as in Fig. 2 (B) Relative protein concentration as a function of time for a random selection of single cells entering starvation. Each panel corresponds to a promoter used in the overflow experiments. The protein concentration of the single cells is scaled to the average concentration across the whole population during exponential growth. Only the first 20 hours of starvation are shown. Chromosomal promoters are indicated by an asterisk next to the name, with black, red and yellow asterisks indicating GFPmut2, mCherry and mVenus, respectively.

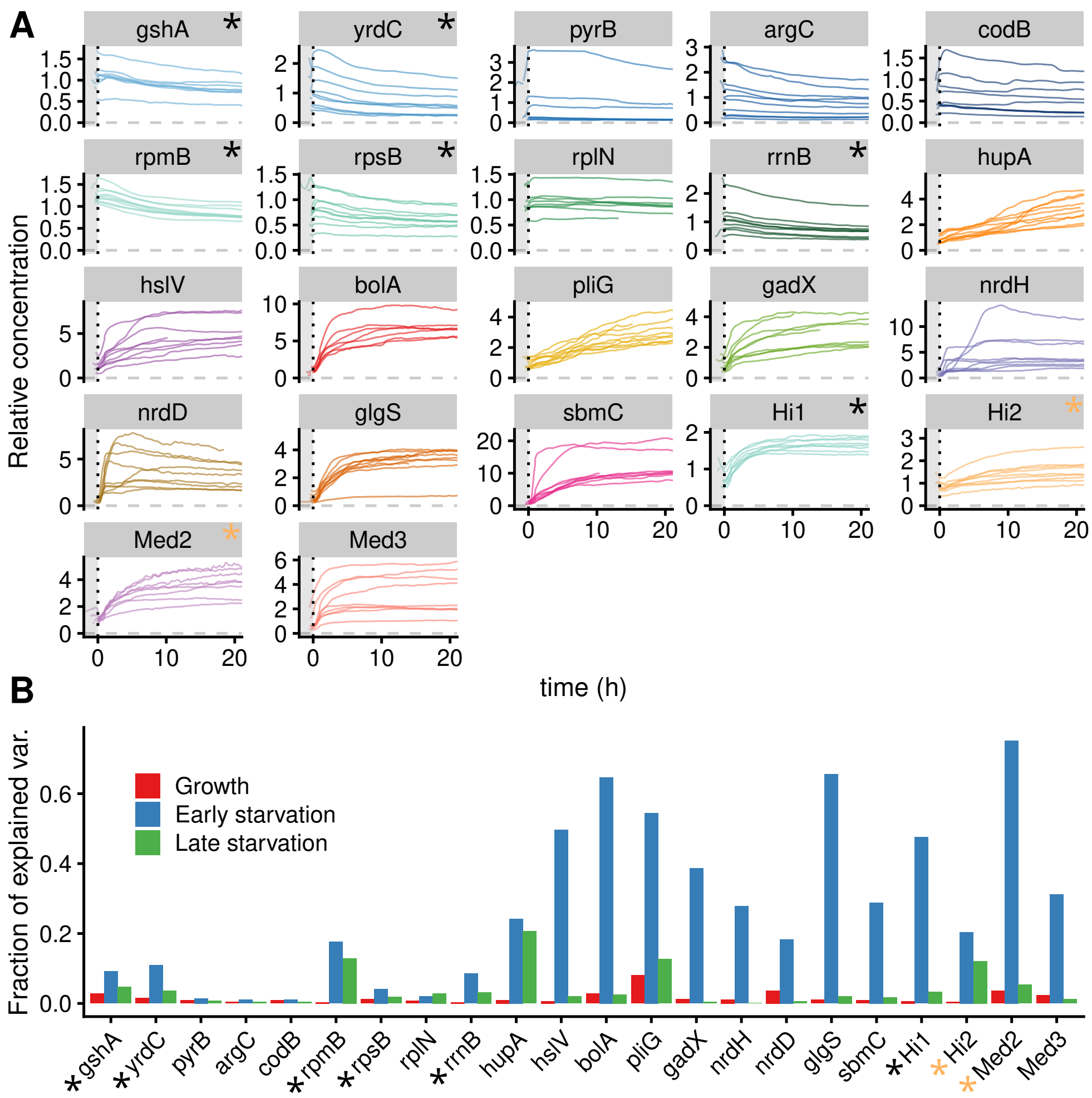

Appendix Figure S17. Changes of concentration upon entry into starvation are consistent across individual cells. (A) Protein concentration traces for a random selection of individual bacteria entering starvation. The concentration in single cells is normalized by the average concentration across the whole population during exponential growth. Only the first 20 out of 60 hours of starvation are shown. Each panel corresponds to one promoter (indicated at the top). (B) The fraction ρ of total variance captured by the variance in average concentration (computed as explained in the main text) is systematically higher during the first 10h of starvation (blue), compared to exponential growth (red) and late starvation (green). Unless otherwise indicated the fluorescent reporter is GFPmut2 transcribed from a low-copy number plasmid. Chromosomal promoters are indicated by an asterisk next to the name, with black, red and yellow asterisks indicating GFPmut2, mCherry and mVenus, respectively.

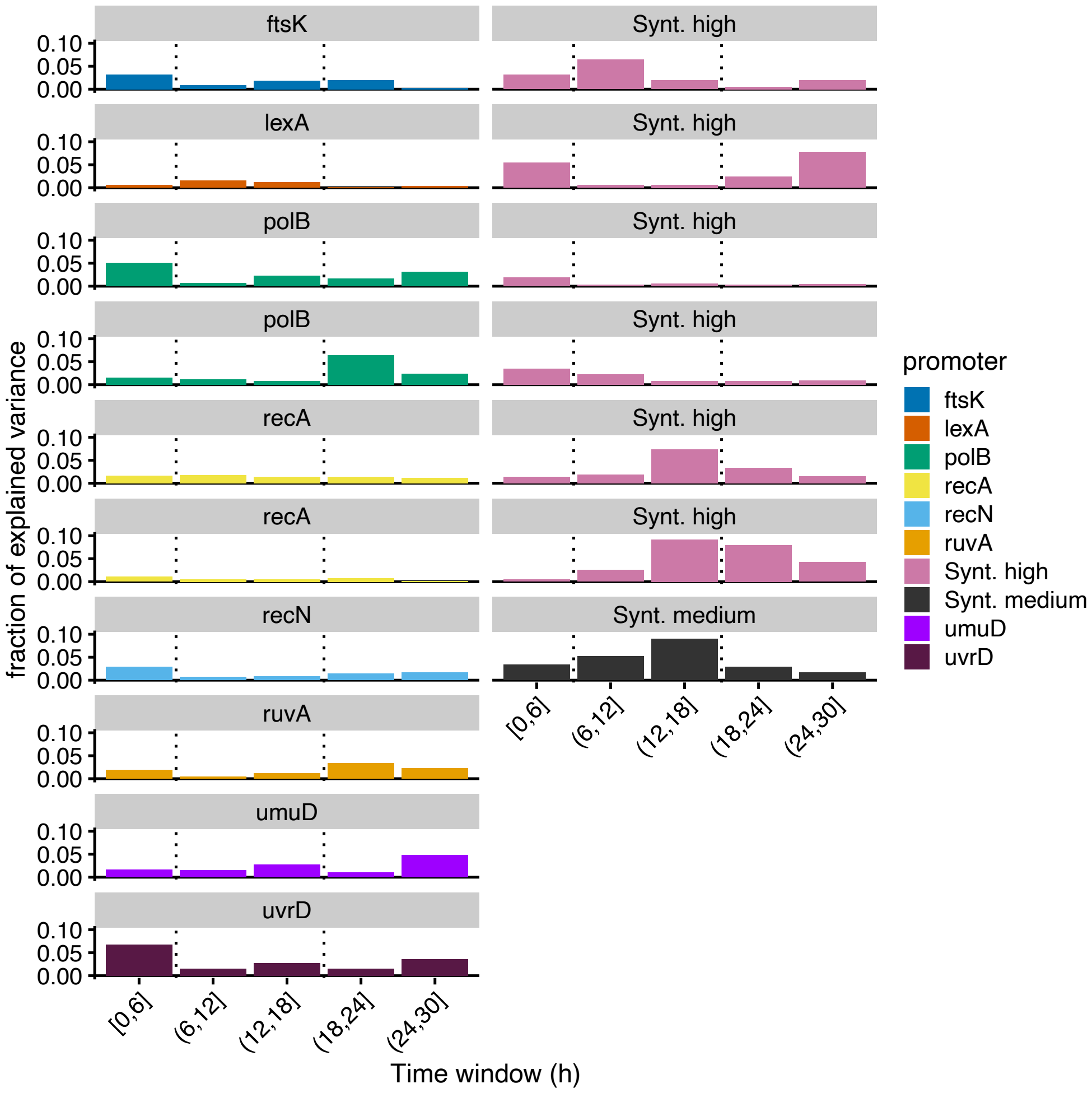

Appendix Figure S18. Fraction of explained variance ρ for target promoters of the LexA regulon under increasing subinhibitory doses of ciprofloxacin. We re-analysed data from Galbusera et al. (2025) where a very similar experimental protocol was used to measure the single-cell responses of LexA target promoters to low levels of DNA damage induced by ciprofloxacin. Cells were grown for 6 hours without cipro, followed by 12 hours at 1 ng/mL, and finally 12 hours at 2 ng/mL. Each panel shows the fraction ρ of the total variance in gene expression across all cells and time that is captured by the time-dependent population average expression (computed as explained in the main text), for one promoter in one replicate experiment and different time bins. The panels on the left correspond to LexA target promoters and the panels on the right to synthetic constitutive promoters as a reference. Note that, because responses are highly variable across single cells, ρ for LexA targets is virtually never above 5%

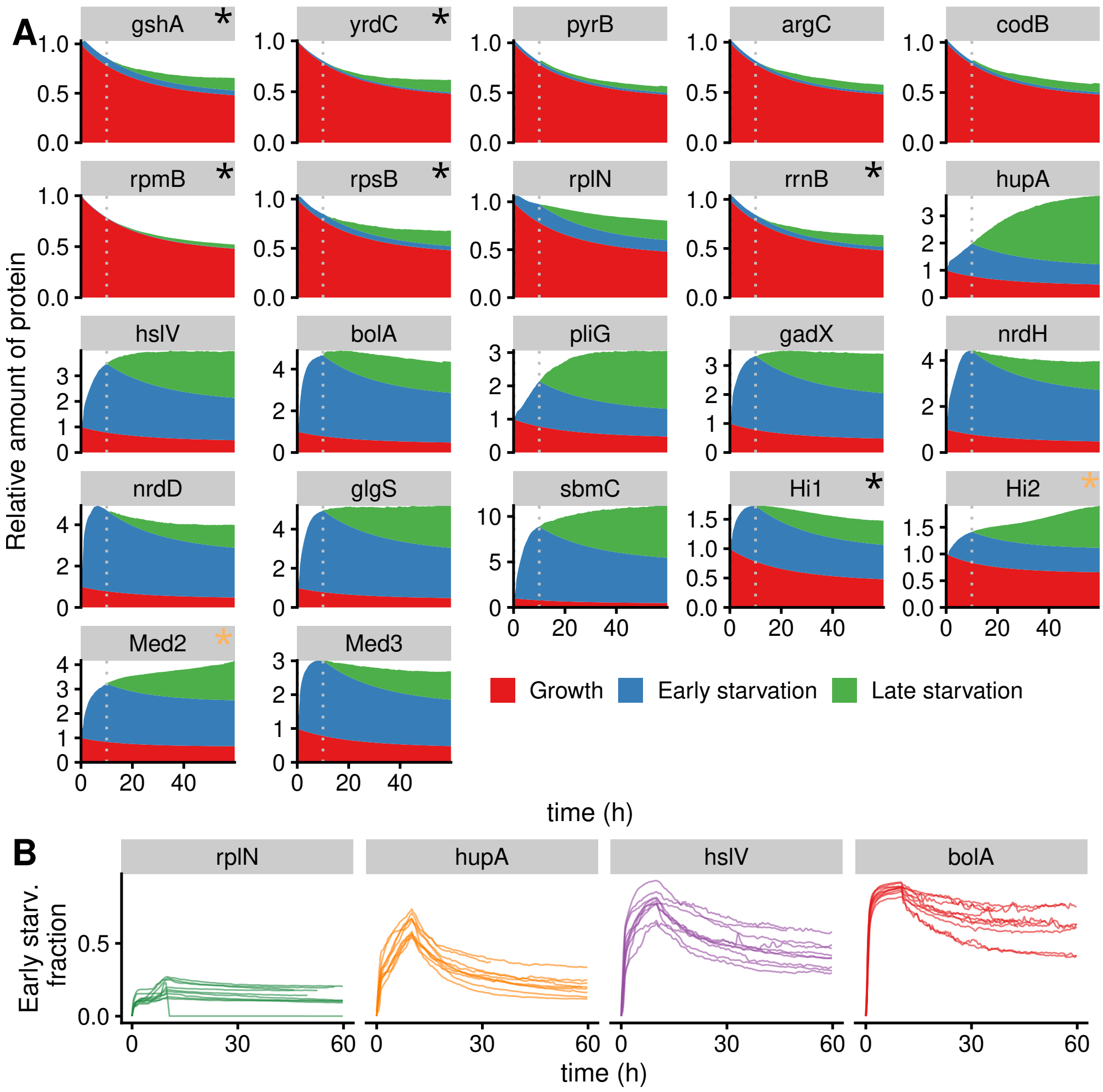

Appendix Figure S19. Relative contributions to protein concentrations from protein production during different periods. (A) Each panel shows the relative amount of total fluorescent protein at a given time during starvation that derives from production during growth, early starvation, and late starvation for a given promoter (i.e. as in Fig. 5A for all promoters). Early starvation is defined as $0 - 10$ h of starvation and late starvation as $10 - 60$ h. Amounts are shown relative to the amount of protein at the start of starvation. Unless otherwise indicated the fluorescent reporter is GFPmut2 transcribed from a low-copy number plasmid. Chromosomal promoters are indicated by an asterisk next to the name, with black, red and yellow asterisks indicating GFPmut2, mCherry and mVenus, respectively. (B) Fraction of the total fluorescent proteins existing at a given time that derive from production during the first 10 hours of starvation in individual cells. Each panel corresponds to one promoter and shows protein fractions for a randomly selected subset of cells. Only the 4 promoters we focus on in the main text are shown here.

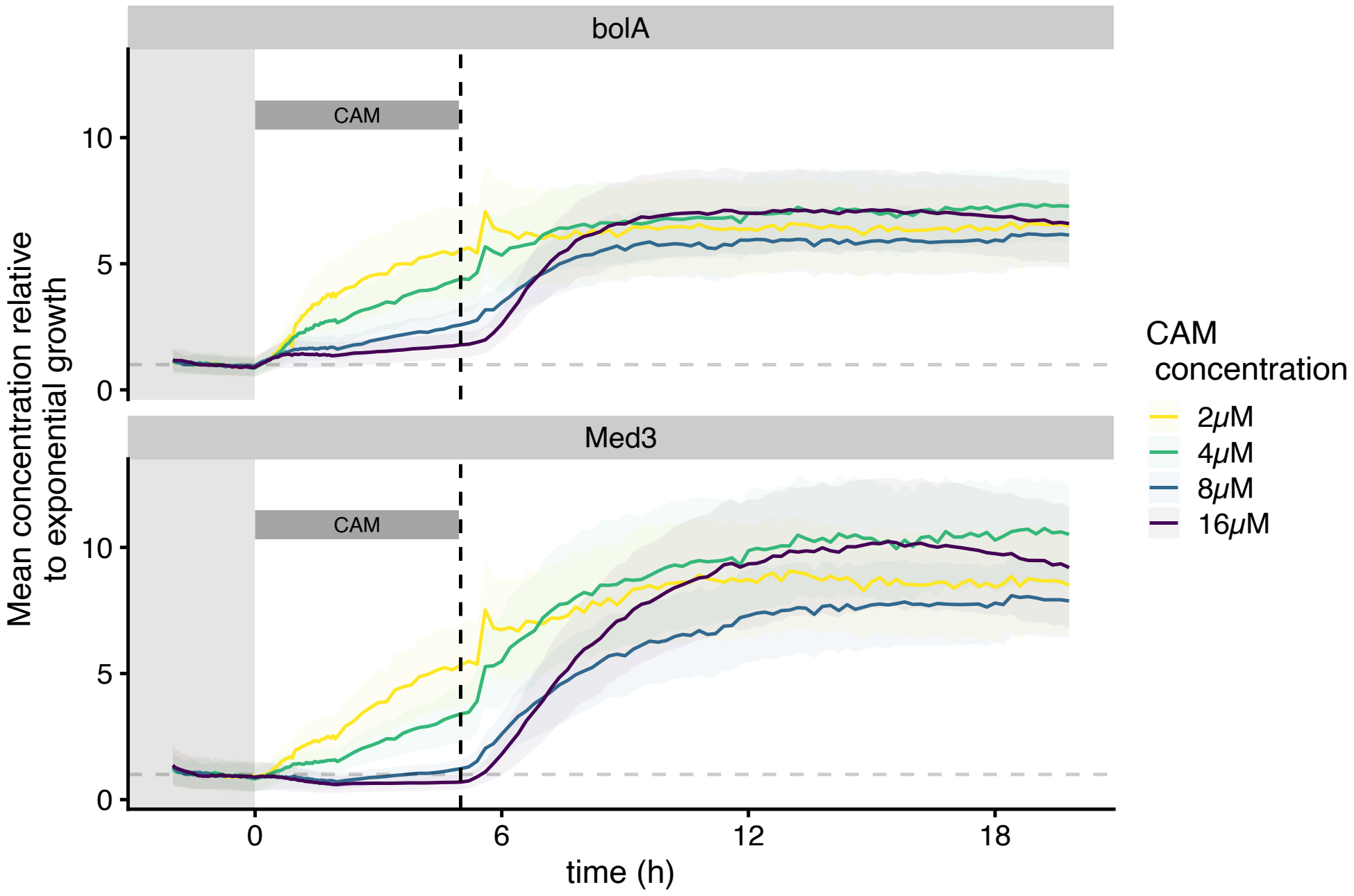

Appendix Figure S20. Gene expression dynamics of RpoS targets upon entry into starvation under increasing inhibition of protein synthesis. Chloramphenicol (CAM) was added at different concentrations (colors) at the start of starvation (time 0) and removed after 5 hours (indicated by the vertical dashed line and CAM box). Solid lines correspond to the population average concentration as a function of time, normalized by the average concentration during exponential growth (dotted line); ribbons correspond to mean ± s.d. Note that in these experiments, Med3 and bolA reporters are chromosomally integrated, and drive the expression of mVenus and mCherry2-L respectively, and not GFPmut2 (see the Reagents and Tools Table and Appendix Table S6 for more details on the strains used in the different experiments).

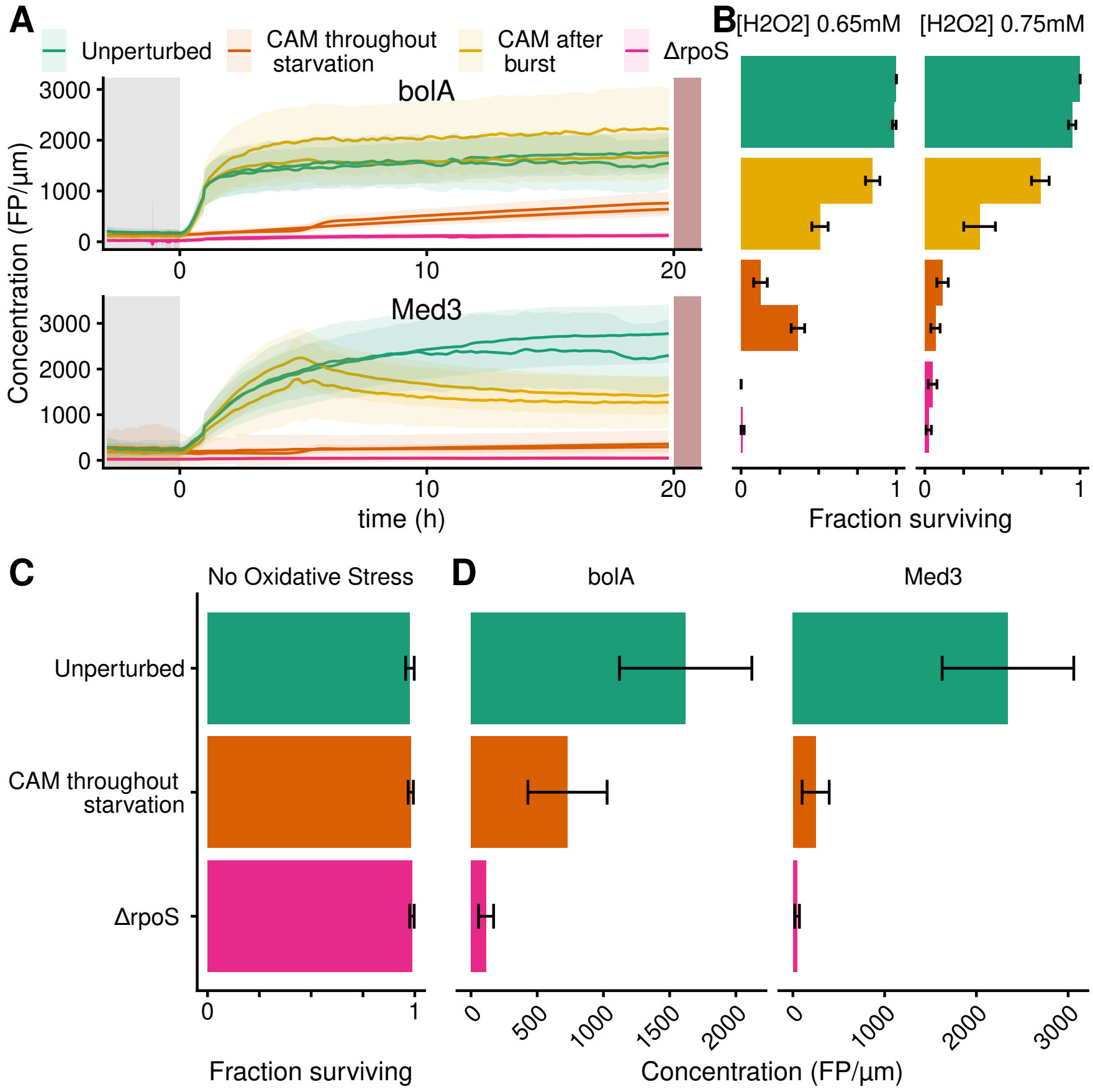

Appendix Figure S21. Impact of gene expression perturbations on survival to oxidative stress during starvation. (A) Average protein concentrations for bolA and Med3 reporters in the oxidative stress challenge experiments. Ribbons indicate mean ± s.d. The different colors correspond to different inhibition as described at the top and explained in Fig. 6 (2 replicates per treatment). (B) Fraction of surviving cells for two different peroxide concentrations (left and right) and different replicates (individual bars) for different treatments (colors). These data underlie the results shown in Fig. 6B. (C) Fraction of surviving cells for all control conditions without oxidative stress. These data underlie results shown in Fig. 6B. (D) Average concentration after 20 hours of starvation in control experiments where no stress was used. The results in panels C and D show that adding chloramphenicol or deleting rpoS does not by itself affect the survival of starved cells on this timescale. Note that in these experiments, Med3 and bolA reporters are chromosomally integrated, and control the expression of mVenus and mCherry2-L respectively, and not GFPmut2 (see the Reagents and Tools Table and Appendix Table S6 for more details on the strains used in the different experiments).
