# Supplementary material for: E. coli prepares for starvation by dramatically remodeling its proteome in the first hours after loss of nutrients
Source: Mol Syst Biol. 2026 Jul 1;22(10):1531–50. doi: 10.1038/s44320-026-00226-5 (PMC13639054; doi:10.1038/s44320-026-00226-5)
Supplement: Supplementary file 2 [file 44320_2026_226_MOESM2_ESM.pdf]

E. coli prepares for starvation by dramatically remodeling its proteome in the first hours after loss of nutrients

Théo Gervais, Bjoern Kscheschinski, Michael Mell, Nevil Goepfert, Erik van Nimwegen, and Thomas Julou

Corresponding author(s): Thomas Julou (thomas.julou@unibas.ch) , Erik van Nimwegen (erik.vannimwegen@unibas.ch)

Review Timeline:

Submission Date:	3rd Jun 25
Editorial Decision:	21st Jul 25
Revision Received:	29th Dec 25
Editorial Decision:	31st Mar 26
Revision Received:	29th Apr 26
Editorial Decision:	18th May 26
Revision Received:	27th May 26
Accepted:	9th Jun 26

Editor: Poonam Bheda

Transaction Report:

(Note: With the exception of the correction of typographical or spelling errors that could be a source of ambiguity, letters and reports are not edited. Depending on transfer agreements, referee reports obtained elsewhere may or may not be included in this compilation. Referee reports are anonymous unless the Referee chooses to sign their reports.)

21st Jul 2025

Manuscript Number: MSB-2025-13152-T
Title: E. coli prepares for starvation by dramatically remodeling its proteome in the first hours after loss of nutrients

Dear Dr. Julou,

Thank you again for submitting your work to Molecular Systems Biology. We have now heard back from the three reviewers who agreed to evaluate your study. As you will see from the reports below, the referees acknowledge the interest of the study and are overall supportive of your work; however, they also raise a series of concerns, which we would ask you to address in a major revision.

Without repeating all the comments listed below, some of the more fundamental issues raised are the following:
- additional controls are needed to ensure appropriate interpretation of the results and robustness of the conclusions
- discussion of previous literature, embedding of current results into past research, and additional discussion on potential reasons for inconsistencies with previous findings

All other issues raised would need to be satisfactorily addressed. Please let me know in case you would like to discuss in further detail any of the any of the reviewer comments or your proposed revisions, I would be happy to schedule a call.

When submitting your revised manuscript, please carefully review the instructions that follow below. We perform an initial quality control of all revised manuscripts before re-review; failure to include requested items will delay the evaluation of your revision.

We require:

1) A .docx formatted version of the manuscript text (including legends for main figures, EV figures and tables). Please make sure that the changes are highlighted to be clearly visible. Alternatively you may choose to submit your manuscript as a LaTeX file.

2) Individual production quality figure files as .eps, .tif, .jpg (one file per figure). For guidance, download the 'Figure Guide PDF' (https://www.embopress.org/page/journal/17574684/authorguide#figureformat).

3) At EMBO Press we ask authors to provide source data for the main figures. Our source data coordinator will contact you to discuss which figure panels we would need source data for and will also provide you with helpful tips on how to upload and organize the files.

4) A .docx formatted letter INCLUDING the reviewers' reports and your detailed point-by-point responses to their comments. As part of the EMBO Press transparent editorial process, the point-by-point response is part of the Peer Review File (PRF), which will be published alongside your paper.

5) A complete author checklist, which you can download from our author guidelines (https://www.embopress.org/page/journal/17574684/authorguide#submissionofrevisions). Please insert information in the checklist that is also reflected in the manuscript. The completed author checklist will also be part of the PRF.

6) Please note that all corresponding authors are required to supply an ORCID ID for their name upon submission of a revised manuscript.

7) It is mandatory to include a 'Data Availability' section after the Materials and Methods. Before submitting your revision, primary datasets produced in this study need to be deposited in an appropriate public database, and the accession numbers and database listed under 'Data Availability'. Please remember to provide a reviewer password if the datasets are not yet public (see https://www.embopress.org/page/journal/17574684/authorguide#dataavailability).

In case you have no data that requires deposition in a public database, please state so in this section as follows: "This study includes no data deposited in external repositories". Note that the Data Availability Section is restricted to new primary data that are part of this study.

8) All Materials and Methods need to be described in the main text using our 'Structured Methods' format, which is required for all research articles. According to this format, the Methods section includes a Reagents and Tools Table (listing key reagents, experimental models, software and relevant equipment and including their sources and relevant identifiers) followed by a Methods and Protocols section describing the methods using a step-by-step protocol format. The aim is to facilitate adoption of the methodologies across labs. Please upload the Reagents and Tools table as a separate document when submitting your revised manuscript. More information on how to adhere to this format as well as a downloadable template (.docx) for the

Reagents and Tools Table can be found in our author guidelines:
https://www.embopress.org/page/journal/17444292/authorguide#structuredmethods

An example of a Method paper with Structured Methods can be found here:
https://www.embopress.org/doi/10.15252/msb.20178071.

9) For data quantification: please specify the name of the statistical test used to generate error bars and p-values, the number (n) of independent experiments (specify technical or biological replicates) underlying each data point and the test used to calculate p-values in each figure legend. The figure legends should contain a basic description of n, p-values and the test applied. Graphs must include a description of the bars and the error bars (s.d., s.e.m.). Please provide exact p-values (in either the figure or figure legend).

10) Our journal encourages inclusion of *data citations in the reference list* to directly cite datasets that were re-used and obtained from public databases. Data citations in the article text are distinct from normal bibliographical citations and should directly link to the database records from which the data can be accessed. In the main text, data citations are formatted as follows: "Data ref: Smith et al, 2001" or "Data ref: NCBI Sequence Read Archive PRJNA342805, 2017". In the Reference list, data citations must be labeled with "[DATASET]". A data reference must provide the database name, accession number/identifiers and a resolvable link to the landing page from which the data can be accessed at the end of the reference. Further instructions are available at .

11) We replaced Supplementary Information with Expanded View (EV) Figures and Tables that are collapsible/expandable online. EV Figures should be cited as 'Figure EV1, Figure EV2" etc... in the text and their respective legends should be included in the main text after the legends of regular figures.
- For the figures that you do NOT wish to display as Expanded View figures, they should be bundled together with their legends in a single PDF file called *Appendix*, which should start with a short Table of Content. Appendix figures should be referred to in the main text as: "Appendix Figure S1, Appendix Figure S2" etc.
- Additional Tables/Datasets should be labeled and referred to as Table EV1, Dataset EV1, etc. Legends should be provided in a separate tab in case of .xls files. Alternatively, the legend can be supplied as a separate text file (README) and zipped together with the Table/Dataset file.
See detailed instructions here:
https://www.embopress.org/page/journal/17574684/authorguide#expandedview

12) Author contributions: CRediT has replaced the traditional author contributions section because it offers a systematic machine-readable author contributions format that allows for more effective research assessment. Please remove the Authors Contributions from the manuscript and use the free text boxes beneath each contributing author's name in our system to add specific details on the author's contribution. More information is available in our guide to authors.

13) Disclosure statement and competing interests: We updated our journal's competing interests policy in January 2022 and request authors to consider both actual and perceived competing interests. Please review the policy https://www.embopress.org/competing-interests and update your competing interests if necessary.

14) Every published paper now includes a 'Synopsis' to further enhance discoverability. Synopses are displayed on the journal webpage and are freely accessible to all readers. They include a short stand first (maximum of 300 characters, including space) as well as 2-5 one-sentences bullet points that summarizes the paper. Please write the bullet points to summarize the key NEW findings. They should be designed to be complementary to the abstract - i.e. not repeat the same text. We encourage inclusion of key acronyms and quantitative information (maximum of 30 words / bullet point). Please use the passive voice. Please attach these in a separate file or send them by email, we will incorporate them accordingly.

Please also suggest a striking image or visual abstract to illustrate your article as a PNG file 550 px wide x 300-600 px high.

Please note that these would be the final versions and changes during proofing are usually not allowed.

15) As part of the EMBO Publications transparent editorial process initiative (see our policy here: https://www.embopress.org/transparent-process#Review_Process), Molecular Systems Biology will publish online a Peer Review File (PRF) to accompany accepted manuscripts.
In the event of acceptance, this file will be published in conjunction with your paper and will include the anonymous referee reports, your point-by-point response and all pertinent correspondence relating to the manuscript. Let us know whether you agree with the publication of the PRF and as here, if you want to remove or not any figures from it prior to publication.
Please note that the Author checklist will be published at the end of the PRF.

Molecular Systems Biology has a "scooping protection" policy, whereby similar findings that are published by others during review or revision are not a criterion for rejection. Should you decide to submit a revised version, I do ask that you get in touch after three months if you have not completed it, to update us on the status.

Yours sincerely,

Poonam Bheda, PhD
Scientific Editor
Molecular Systems Biology

Reviewer #1:

The work presented in the manuscript by Gervais et al. explores the adaptation of Escherichia coli to carbon starvation at the single cell level, combining experiments of microfluidics and fluorescence microscopy with advanced image and data analysis. Using a multiplexed version of their previously developped Dual Input Mother Machine (DIMM) microfluidic device, the authors follow the growth and expression of more than 20 promoters in bacteria undergoing a shift from exponential growth in glucose-rich medium to growth arrest in a medium with no carbon source. Image analysis was performed using their automated software deepMoMA and data analysis was performed using a bayesian inference procedure that they previously developed to estimate instantaneous growth rates and protein production rates, as well as mathematical modeling. The authors found that upon loss of carbon source all cells stop growing immediately, with a subpopulation undergoing a division, but remain alive for an extended period of time. Gene expression dynamics is found to be very different for different promoters but with limited single-cell variability. In addition, the authors found that the first hours of starvation are particularly important to remodel the proteome and that this remodeling is important for stress protection during the starvation period.

In my opinion this is a really nice work, interesting and rigorous. As pointed out by the authors, starvation is likely to be a situation commonly faced by bacteria in nature, and is however not that much studied. Exploring starvation as it is done here is therefore relevant. In addition, this work combines state-of-the art experimental techniques with a very rigorous and advanced data analysis approach. Therefore, it provides "clean" and reliable data on both growth and gene expression that can constitute bases for future investigations on starvation by the authors or other scientists in the field.

For all these reasons I recommend publication. The paper is clearly written and well organized and in my opinion it can be published as it is. I'd like to share a few comments while leaving the authors free to decide whether they want to address them and change anything in the manuscript.

My main interrogation is about the way single-cell variability is analyzed and interpreted. There is some emphasis in the manuscript on the idea that there is little single-cell variability in the response (in particular in the dynamics of gene expression which is said to be "remarkably homogeneous" across single cells, "highly consistent"...). however the variability shown in the figures (e.g. fig 1C, fig4 , fig S4) seems quite large to me and the statement that it is very homogeneous therefore seems subjective. Although I do agree that the fraction of explained variance is an interesting quantity to look at, and that fig 4C shows that single cells respond in the same way in an "organized" response, here also I feel that the reader is finally left with a subjective decision on whether having 50% of explained variance is high or not. Comparing it to the value in exponential growth does not help since it is a stationary regime and the variance of the average thus vanishes. I would find it very interesting if the explained variance could be compared to the explained variance in other well characterized stress responses. More generally I think it would be interesting if the authors could elaborate more on the quantification of single-cell variability. Otherwise, the text could also simply be reworded to tone down the claim of remarkable homogeneity.

I found interesting the fact that quantitative proteomics was not accurate enough, did the authors compare also their results to transcriptomics data? (the average response for the different promoters)

Reviewer #2:

Review of paper by T Gervais et al.
Title: E. coli prepares for starvation by dramatically remodelling its proteome in the first hours after loss of nutrients

Summary
This study explores how Escherichia coli adapts to carbon starvation at the single-cell level. Using a microfluidic platform, the authors track gene expression dynamics from 22 different transcriptional promoters under conditions of both abrupt and gradual starvation. They aim to analyse the variability of response dynamics across cells and across promoter types, and dissect how early expression changes predict long-term survival and adaptation in the stationary phase.

Key Conclusions

Individual promoters respond distinctly to starvation, but cell-to-cell variability for a given promoter is low.
Growth arrest is nearly immediate after nutrient withdrawal in abrupt starvation, yet the population survives for extended periods without detectable cell death.
Short-term expression dynamics are predictive of longer-term expression patterns, and these early events are posited to shape the stationary-phase proteome.
Gradual and abrupt starvation result in similar long-term expression profiles, leading the authors to suggest convergent adaptive outcomes.

Methodology and Model System
The authors use a mother machine microfluidic device to monitor growth and fluorescence of single E. coli cells expressing fluorescent transcriptional reporters. They compare abrupt vs. gradual removal of carbon sources and examine the dynamics of fluorescent reporter expression as a proxy for gene expression.

General Remarks
Are you convinced of the key conclusions?
While the experimental design is robust and the data provide useful insights into starvation dynamics at the single-cell level, several key conclusions, particularly regarding proteome remodelling, equivalence of starvation modes, accurate expression dynamics inference from the proposed model, and predictive value of early expression, are insufficiently supported by the current data and analysis. Below, I indicate possible sources of mis-interpretations and have suggested some control experiments and necessary refinements in the data interpretation to address these issues.

Context
This work contributes to a long-standing interest in bacterial survival strategies under starvation. Prior studies have investigated maintenance energy requirements, mortality dynamics, and gene expression reprogramming during nutrient limitation. What differentiates this study is the high-resolution, time-resolved single-cell tracking of gene expression in response to starvation. The discussion should highlight this aspect more clearly.

Nature and Significance of the Advance
The study provides a technical advance in applying microfluidics and single-cell imaging to track starvation responses dynamically. Conceptually, it attempts to link early transcriptional events to long-term physiological adaptation. However, the claims about proteome remodelling and the convergence of starvation trajectories require more stringent evidence. Technically the work builds on the platform developed in two previous studies (S Moreno-Gámez et al., PNAS 2020, and Bakshi et al., Nature microbiology, 2021) and integrates the analysis of transcriptional reporters. The analysis of the data builds upon the previous work from the same group. The experimental and analytical approaches are integrated well. The proposed model will benefit from more intuitive discussion of the used dynamic types.

Audience
Researchers in bacterial physiology, systems microbiology, synthetic biology, and microbial ecology will find this work of interest, particularly those focused on stress responses, single-cell dynamics, and growth regulation.

Major Points
Terminology: "Proteome Remodelling" needs to be carefully stated or avoided.
The manuscript refers to proteome remodelling, yet all experimental observations are based on fluorescent transcriptional reporters. These reporters do not account for mRNA half-lives, translation dynamics, or protein degradation, particularly problematic given that GFP-like proteins are known to resist proteolysis. Without proteomics data (e.g., mass spectrometry), the conclusions are better framed in terms of transcriptional programming or gene expression dynamics, rather than proteome changes.

Critical Oversight: Fluorescent Protein Maturation Kinetics
The fluorescence dynamics observed in this study may be strongly confounded by the maturation delay of fluorescent proteins, which is not addressed in the analysis.

Example: For a fluorescent protein like RFP with a maturation time of ~40 minutes, only ~33% of proteins are mature in a cell growing with a 20-minute doubling time. Upon sudden growth arrest, the remaining immature proteins mature without dilution, leading to a ~3-fold increase in fluorescence without any new protein synthesis. This could easily be mistaken for transcriptional induction.

The authors should use databases like Balleza et al., Nature Methods, 2018, to estimate the maturation properties of the FPs they use and incorporate these parameters into their fluorescence-to-expression estimation models.

A systematic comparison using fast- and slow-maturing FPs for the same promoters is recommended to validate whether the dynamics observed reflect biological responses or FP artefacts.

Lack of Methodological Details in the Main Paper Limits Interpretation
There is no dedicated Methods section, making it difficult to assess or replicate the work. Important missing details include:

Which reporters and FP variants were used (along with their known maturation times)
Imaging settings (exposure, intervals, illumination intensity), needed to assess photobleaching and noise
Calibration of OD sensors, fluidic switching protocols, and thresholds used for growth halt detection

This information is essential for understanding the trade-offs in time resolution, photobleaching, and interpretability of the fluorescence data.

Claimed Convergence of Abrupt and Gradual Starvation Is Not Fully Supported
The authors report similar long-term transcriptional reporter dynamics between abruptly and gradually starved cells. However, this alone is insufficient to conclude similar physiological states or proteome outcomes.

A comparison of lag time distributions during resuscitation is necessary to support the claim of functional equivalence. Previous studies, including work from Nathalie Balaban's lab, have shown that starvation dynamics crucially influence recovery kinetics.

Similarly, quantitative proteomic comparisons between the two modes of starvation would strengthen the conclusion about convergence.

Use of Diverse Reporters Without Systematic Controls
The authors use a variety of fluorescent reporters for different promoters, but the choice appears ad hoc. Since different FPs have different maturation and photobleaching rates, this introduces uncontrolled variation. To ensure reliability:

The same promoter should be tested with multiple FPs to isolate promoter dynamics from reporter artefacts.
All promoter-FP pairs should be benchmarked against a common standard (e.g., sfGFP or YFP or mCherry) to allow cross-comparison.

Speculative Interpretations of Degradation and Resource Reallocation
The manuscript suggests that protein degradation slows in starvation and that ribosome repression frees up resources for stress genes. However:

GFP signals do not reflect degradation dynamics, as these proteins resist native proteolysis.
Translation and mRNA decay rates differ across genes and are not captured by the reporters.
Such interpretations are speculative without direct measurements and should be clearly labeled as hypotheses.

Inadequate Consideration of rpoS Dynamics
The observation that rpoS-regulated genes are expressed before starvation is puzzling, given that RpoS is actively degraded in the exponential phase (half-life ~30 s). The mechanism underlying pre-starvation expression requires clarification or supporting evidence.

Minor Points
Introduction: The claim that exponential phase is studied "because it is easy" misrepresents the rationale. It is used because it represents a reproducible, steady-state growth condition suitable for perturbation studies.

Literature: The statement that little is known about mortality during starvation ignores recent key studies: Biselli et al., mBio - showing longer survival with slower growth, Schink et al., eLife - modeling death rates under starvation. These should be cited and discussed in context.

Figure 2b: The schematic of promoter classes is helpful, but it would benefit from hypothesized underlying mechanisms or regulatory motifs.

Mother machine implications: Since the device imposes no selective pressure and cells in gradual starvation arrest asynchronously, it would be useful to discuss how this affects the comparison with bulk measurements.

Terminology and clarity: Distinguish clearly between gene expression, protein abundance, and proteome-level changes throughout the text.

Conclusion
This paper presents a technically elegant and potentially valuable dataset on how E. coli responds to nutrient starvation at the single-cell level. However, the analysis of results ignores the considerations for the photophysics of the reporter proteins which can substantially affect the interpretations, and several key conclusions, particularly regarding proteome remodelling and convergence of starvation trajectories, are not sufficiently substantiated.

The study would benefit substantially from:

Reframing claims to reflect transcriptional dynamics rather than proteomic changes;
Incorporating maturation-aware modeling of fluorescence dynamics;
Adding methodological transparency and systematic controls; e.g. Controls for similar FPs for different promoters and different FPs for the same promoters are needed to verify the results.
Including additional functional comparisons (e.g., lag time, resuscitation dynamics) to validate claimed physiological similarities between abrupt and gradual starvation.
Studying other growth media for general claims about the observed dynamics and their implications

Addressing these concerns will improve the clarity, rigor, and impact of the study.

Reviewer #3:

Gervais et al. combine microfluidics with fluorescence microscopy to quantify gene expression in E. coli stationary phase. They show that protein production and degradation prepares cells for stress during the first 5-10 hours, which is important for stress survival. Importantly, they demonstrate at the single cell level that there is not a large heterogeneity in gene expression. These are interesting results, and the work could in principle be well suited for MSB. However, we have major concerns about the experimental methodology and inconsistencies with previous findings. Important control experiments are also missing.
Major points

1. It is important to understand to what degree bacteria in the experiment are starved. It has been observed consistently that E. coli and many other bacteria die when carbon runs out (see Yang et al., 2019 [DOI: 10.1126/sciadv.aaw2069], Reeve et al, 1984 [https://journals.asm.org/doi/epdf/10.1128/jb.157.3.758-763.1984], Biselli et al 2020 [https://doi.org/10.15252/msb.20209478]). The experiments by Gervais et al. are more consistent with findings that bacteria survive (yet not grow) if there is a small amount of nutrient left in the medium (see e.g. Phaiboun et al. 2015, [https://doi.org/10.1371/journal.pcbi.1004198]). Microfluidics can cause leaking nutrients into the medium, and a small amount of nutrients may strongly affect survival. Nevertheless, Yang et al. 2019 [DOI: 10.1126/sciadv.aaw2069] succeeded to show bacterial aging in microfluidics - in their experiments E. coli dies off by around 50% at 60h in starvation, which may not be consistent with these findings. Therefore, the authors should perform control experiments to measure viability during the first 60h of starvation in batch, after performing a hard shift with washing with carbon free medium. If batch culture E. coli die substantially faster in batch after a hard shift than in the mother machine for ~60h, then we propose the authors remove the claim that they are measuring starvation and instead label their experiments as stationary phase (similar to previous work PMID: 24344288).
2. We have concerns that the illumination is affecting cellular physiology. Fig. S16A in the supporting Information appears to indicate that only half of the bacteria are able to regrow after 60h with illumination, but >90% without illumination? The remaining viable bacteria could also be affected by the illumination. Such stress could affect the bacteria's ability to synthesize proteins. If this is the case, the reader should be made aware of this potential limitation, and it should be discussed in the main text, possibly with additional control experiments testing the impact of this limitation. Such control experiments should show the difference between mother machine and batch culture without illumination and show the magnitude of the effect on protein-fold changes. The batch control could either be proteomics or measurements using the authors' fluorescence reporters. Measurements of the fluorescence reporters in bulk could be done by taking a sample prior to starvation and a sample at 60h into starvation and measuring single-cell fluorescence under the microscope. The authors may design their own experiments, but it is important that these experiments clarify the effect of the experimental setup on the measurements.
3. In the 'soft shift' experiment a glucose batch culture is connected to the microfluidics. However, acetate is excreted to the medium during growth on glucose, which means that cells may not really be in starvation. While cells are not growing, acetate can be utilized as an energy source, which may affect phenotypes. This experiment may be fine as a characterization of a non-growing stationary phase state, but if the authors want to claim that cells are in starvation, this issue should be addressed.

Minor points
1. Overall, the paper needs to embedded better into the scientific literature. The introduction and discussion fail to connect the work to past research on microbial starvation. Key topics are missing, such as prior work using microfluidics to study starvation and prior work on gene expression during starvation. The discrepancy with other works where bacteria die faster such as Yang et al. 2019 may need to be discussed.

Detailed responses to the comments of reviewer #1

Comment 1.1: *The work presented in the manuscript by Gervais et al. explores the adaptation of Escherichia coli to carbon starvation at the single cell level, combining experiments of microfluidics and fluorescence microscopy with advanced image and data analysis. [...]*

In my opinion this is a really nice work, interesting and rigorous. As pointed out by the authors, starvation is likely to be a situation commonly faced by bacteria in nature, and is however not that much studied. Exploring starvation as it is done here is therefore relevant. In addition, this work combines state-of-the art experimental techniques with a very rigorous and advanced data analysis approach. Therefore, it provides "clean" and reliable data on both growth and gene expression that can constitute bases for future investigations on starvation by the authors or other scientists in the field.

For all these reasons I recommend publication. The paper is clearly written and well organized and in my opinion it can be published as it is. I'd like to share a few comments while leaving the authors free to decide whether they want to address them and change anything in the manuscript.
Response 1.1: We thank the reviewer for these graceful comments.
Comment 1.2: *My main interrogation is about the way single-cell variability is analyzed and interpreted. There is some emphasis in the manuscript on the idea that there is little single-cell variability in the response (in particular in the dynamics of gene expression which is said to be "remarkably homogeneous" across single cells, "highly consistent"...). However the variability shown in the figures (e.g. fig 1C, fig4 , fig S4* ***(now S7)****) seems quite large to me and the statement that it is very homogeneous therefore seems subjective. Although I do agree that the fraction of explained variance is an interesting quantity to look at, and that fig 4C shows that single cells respond in the same way in an "organized" response, here also I feel that the reader is finally left with a subjective decision on whether having 50% of explained variance is high or not. Comparing it to the value in exponential growth does not help since it is a stationary regime and the variance of the average thus vanishes.*

I would find it very interesting if the explained variance could be compared to the explained variance in other well characterized stress responses. More generally I think it would be interesting if the authors could elaborate more on the quantification of single-cell variability. Otherwise, the text could also simply be reworded to tone down the claim of remarkable homogeneity.
Response 1.2: We agree with the reviewer that the amount of variability in the response across single cells is a bit a case of 'glass half full or glass half empty'. We were genuinely struck by the fact that, although there is of course substantial variability across single cells, the qualitative shape of the response of each promoter is quite consistent across cells, even though it is highly variable across promoters (i.e. as shown in Fig S7). Note that, in the revision we improved the readability of the individual traces in this figure by adjusting the shading.

To address the reviewer's comment, we now contrast the observed consistency of the starvation response with that of another regulatory response to stress. In particular, our lab recently analyzed in detail the single-cell responses of the LexA regulon to DNA damage as induced by low levels of ciprofloxacin [1].

We find that, although the fold-changes at the population level are comparable, there is far greater variability across single cells in the DNA damage response than in the starvation response. Specifically, the fraction of variance explained by the population average is less than 5% for the DNA damage response compared to up to 50% for the starvation response (Figs S17 and S18). The reason for the low consistency of the DNA damage response is that, as analyzed

in detail in [1], the response consists of asynchronous stochastic bursts of expression of LexA target promoters that are likely driven by individual DNA damage events in single cells.

We thank the reviewer for raising this issue and believe this additional analysis provides important context to our observation of the consistency of the single cell responses.

Comment 1.3: *I found interesting the fact that quantitative proteomics was not accurate enough, did the authors compare also their results to transcriptomics data? (the average response for the different promoters)*

Response 1.3:

Our initial motivation for performing and analysing quantitative proteomics experiments was to confirm that the protein concentration changes predicted by our measurements of transcriptional reporters quantitatively match native protein levels. Similar to the reviewer, we were also disappointed that time-resolved concentration measurements of individual proteins are not sufficiently accurate for this task. However, for the revision we have made an overall summary comparison with proteomics, integrating data from multiple datasets, to show proteomics results are at least consistent with our observations.

Unfortunately, transcriptomics cannot directly address the issue of protein concentration fold-changes because protein concentrations depend not only on mRNA levels but also on translation and protein decay rates.

However, we agree with the reviewer that it is instructive to compare our single-cell measurements of transcriptional reporters with bulk transcriptome measurements. In our revision, we have included a comparison with transcriptome measurements performed in similar conditions [2]. Although, as discussed in Appendix section 6 of our revision, there are several challenges to making quantitative comparisons (e.g. the unknown relative capture rates in RNA-seq, the fact that RNA-seq does not measure absolute but only relative levels, and the fact that volumic production rates also depend on translation rates), we show that the transcriptome data are generally consistent with our observations (Fig. S9).

Detailed responses to the comments of reviewer #2

Comment 2.1: ***Summary***

This study explores how Escherichia coli adapts to carbon starvation at the single-cell level. Using a microfluidic platform, the authors track gene expression dynamics from 22 different transcriptional promoters under conditions of both abrupt and gradual starvation. They aim to analyse the variability of response dynamics across cells and across promoter types, and dissect how early expression changes predict long-term survival and adaptation in the stationary phase.

Key Conclusions

Individual promoters respond distinctly to starvation, but cell-to-cell variability for a given promoter is low. Growth arrest is nearly immediate after nutrient withdrawal in abrupt starvation, yet the population survives for extended periods without detectable cell death. Short-term expression dynamics are predictive of longer-term expression patterns, and these early events are posited to shape the stationary-phase proteome. Gradual and abrupt starvation result in similar long-term expression profiles, leading the authors to suggest convergent adaptive outcomes.

Methodology and Model System

The authors use a mother machine microfluidic device to monitor growth and fluorescence of single E. coli cells expressing fluorescent transcriptional reporters. They compare abrupt vs.

gradual removal of carbon sources and examine the dynamics of fluorescent reporter expression as a proxy for gene expression.

Response 2.1: While this is a reasonable summary of some of our work, we note that this summary leaves out various important novel results that we establish, including: the way in which both protein degradation and protein production rates change with time, the way the latter differs across promoters, the reductive divisions in cells further along their cell cycle, and the crucial role of expression early in stationary phase for stress resistance later on. We note that because we feel that, unfortunately, this down-playing of our results is pervasive in this reviewer's review.

Second, when mentioning our comparison of gene expression responses to abrupt and gradual starvation, the reviewer claims that we 'suggest convergent outcomes'. Although this point will be further discussed below, we already like to mention that we made no such claims. In fact, we took care to *only* claim that the expression responses are similar. The main focus of our work is on the abrupt switch and we mainly studied the gradual response to preempt concerns that the much more controlled abrupt switch would be 'too unnatural', i.e. that the response of the cells under such an abrupt switch cannot be compared with what would happen under a more 'natural' loss of carbon source. Our comparison shows this not to be the case: the expression responses are highly similar. But we are not making any claims beyond this, and we paid special attention to describe this comparison as restricted to the gene expression dynamics, rather than suggesting implications on survival or regrowth dynamics.

Given that the reviewer seems to nonetheless have gotten the impression that we make larger claims, we have for the revision carefully reviewed every sentence related to this comparison and made sure to stress its limitations.

Comment 2.2: ***General Remarks***

Are you convinced of the key conclusions? While the experimental design is robust and the data provide useful insights into starvation dynamics at the single-cell level, several key conclusions, particularly regarding proteome remodelling, equivalence of starvation modes, accurate expression dynamics inference from the proposed model, and predictive value of early expression, are insufficiently supported by the current data and analysis. Below, I indicate possible sources of mis-interpretations and have suggested some control experiments and necessary refinements in the data interpretation to address these issues.

Response 2.2: We reply below in the places where the reviewer expresses his concrete concerns. However, with regard to the reviewer's claims of 'insufficient support', 'misinterpretations' and 'control experiments and necessary refinements', we would like to already point out that several of the controls that the reviewer claims were missing were in fact present in our manuscript (e.g. proteomics experiments, comparison of transcription reporters with different fluorescent reporters). Other criticisms of this reviewer were either misguided (e.g. the presumed importance of photophysics) or factually inaccurate (e.g. that GFP degradation is exceptional or that rpoS targets are not expressed in exponential phase).

Comment 2.3: ***Context***

This work contributes to a long-standing interest in bacterial survival strategies under starvation. Prior studies have investigated maintenance energy requirements, mortality dynamics, and gene expression reprogramming during nutrient limitation. What differentiates this study is the high-resolution, time-resolved single-cell tracking of gene expression in response to starvation. The discussion should highlight this aspect more clearly.

Nature and Significance of the Advance

The study provides a technical advance in applying microfluidics and single-cell imaging to track starvation responses dynamically. Conceptually, it attempts to link early transcriptional events to long-term physiological adaptation. However, the claims about proteome remodelling and the convergence of starvation trajectories require more stringent evidence. Technically the work builds on the platform developed in two previous studies (S Moreno-Gámez et al., PNAS 2020, and Bakshi et al., Nature microbiology, 2021) and integrates the analysis of transcriptional reporters. The analysis of the data builds upon the previous work from the same group. The experimental and analytical approaches are integrated well. The proposed model will benefit from more intuitive discussion of the used dynamic types.

Audience

Researchers in bacterial physiology, systems microbiology, synthetic biology, and microbial ecology will find this work of interest, particularly those focused on stress responses, single-cell dynamics, and growth regulation.

Response 2.3: We respectfully feel that this summary again fails to properly recognize the advance we provide by ignoring many important results that we present.

First, we are the first to track and quantify gene expression at the single-cell level across time for a wide selection of promoters. Second, the reviewer ignores our novel results on growth arrest and reductive divisions. Third, the reviewer also ignores our novel results on long-term protein production, its variation across promoters (e.g. Fig 1E), and the correlation of late and early production (Fig 5). Fourth, the reviewer completely ignores our results that establish that expression early in stationary phase is crucial for surviving stresses later in stationary phase (Fig 6). Describing our results as merely 'attempting to link' our observations to long-term physiological adaptation in spite of the concrete results presented in Fig. 6 is not just unfair, but comes across as hostile to our work.

With regard to the way our study 'builds' on technical developments, we feel that it is rather arbitrary to single out the use of overflow of batch culture media, which concerns only a minor fraction of our experimental results. Moreover, we clearly cited Gefen et al. [3] and Bakshi et al. [4] for the coupling of batch culture with single-cell microfluidic devices to study bacterial stationary phase. From a technical point of view, our work builds directly on the combined microfluidic/image-analysis setup we first described in [5] and developed further in a number of works since, e.g. [6,7]. For this work, we developed our own "overflow" setup independently, building on this prior work in our lab. Notably, an equally important technical development in this work is the use of multiplexed microfluidic chips that allow monitoring multiple reporters in parallel. Finally, arguably the main technological advance used in this study is the new method for careful quantification of instantaneous rates of growth and gene expression, which is now described in detail in Kscheschinski et al. 2025 (`https://doi.org/10.1101/2025.09.12.675772`).

We will respond to the comments about proteome remodeling claims below (when the reviewer specifies concrete criticisms). We already commented above on what it is we claim about the similarity of the responses to sudden or more gradual loss of carbon source.

Comment 2.4: ***Major Points***

Terminology: "Proteome Remodelling" needs to be carefully stated or avoided. The manuscript refers to proteome remodelling, yet all experimental observations are based on fluorescent transcriptional reporters. These reporters do not account for mRNA half-lives, translation dynamics, or protein degradation, particularly problematic given that GFP-like proteins are known to resist proteolysis.

Without proteomics data (e.g., mass spectrometry), the conclusions are better framed in terms of transcriptional programming or gene expression dynamics, rather than proteome changes.

Response 2.4: While we agree with the reviewer that care needs to be taken regarding exactly what the transcriptional reporters imply about proteome changes, this comment again feels unnecessarily hostile and is factually incorrect in several respects.

First, the reviewer objects to the use of the phrase 'proteome remodelling' based on measurements with transcriptional reporters, apparently suggesting that we cannot assume that major changes in transcription would affect the proteome in any way. This is not reasonable. It is well established that transcriptional changes are the main driver of changes of protein concentrations, e.g. [8]. Indeed, this is the main reason why transcriptional reporters are used widely to study regulatory responses in bacteria [9]. Surely the reviewer must accept that major changes in transcription will remodel the proteome.

Second, contrary to the claims of the reviewer, we actually did include results from proteomics experiments (i.e. what is now Fig S11 in the revision). As we mentioned in the original manuscript (and the reviewer apparently overlooked), proteomics measurements are unfortunately too variable (also across various studies as shown in Fig S11) to make a meaningful quantitative comparisons of the predicted fold-changes. However, large-scale patterns (like down-regulation of ribosomal proteins and up-regulation of RpoS target promoters) are also seen in the mass spectrometry data. We expanded our analysis and showed that, taken together, the different proteomics studies available are consistent with our conclusions drawn from the mother machine experiments (Fig S12 in the revision).

The statement that "GFP-like proteins are known to resist proteolysis" is not supported by the literature as we discuss in Response 2.10, which addresses a similar, more detailed comment.

Besides consistency with proteomics, for the revision we now further show consistency with transcriptomic measurements (Fig S9), which alleviates concerns that variation in mRNA stability is a major driver of expression changes. In addition, we note that although we use transcriptional reporters, the readout is still in terms of protein level (i.e amount of GFP) which, in contrast to transcriptomics or FISH, also depends on the overall time-dependent rate of protein production. Indeed, our measurements also capture how protein production decays to low levels after the first 10-15 hours in starvation.

Comment 2.5: ***Critical Oversight: Fluorescent Protein Maturation Kinetics***

The fluorescence dynamics observed in this study may be strongly confounded by the maturation delay of fluorescent proteins, which is not addressed in the analysis.

Example: For a fluorescent protein like RFP with a maturation time of 40 minutes, only 33% of proteins are mature in a cell growing with a 20-minute doubling time. Upon sudden growth arrest, the remaining immature proteins mature without dilution, leading to a 3-fold increase in fluorescence without any new protein synthesis. This could easily be mistaken for transcriptional induction.

The authors should use databases like Balleza et al., Nature Methods, 2018, to estimate the maturation properties of the FPs they use and incorporate these parameters into their fluorescence-to-expression estimation models.

Response 2.5: We completely agree with the criticism of the reviewer that we ignored to properly discuss the role of fluorescent protein maturation. We did consider this aspect in our experimental design. In particular, the GFP that was used for most reporters (GFPmut2) has a very short maturation time on the order of 5 minutes [10], which is fast compared to the doubling time in exponential phase (about 65 minutes). That said, it is true that, in some experiments

where we had dual reporter strains, we also used transcriptional reporters with other fluorescent proteins (mVenus and mCherry2-L) and these have larger maturation times, which we wrongly ignored in our analysis.

For the revision we have redone our analysis of volumic production and protein concentrations to explicitly take the maturation of fluorescent proteins into account (Appendix sections 1.3 and 3, and Fig S3). As shown in Fig S3, the correction for maturation has a minimal effect for production estimated using GFPmut2, but for mVenus and mCherry there is a noticeable time-shift in the inferred production traces. In the revision we have now explicitly corrected the inferred production traces and protein concentration estimates for results from mVenus and mCherry reporters (using the maturation rates reported in Balleza, et al. 2018 [10]).

Comment 2.6: *A systematic comparison using fast- and slow-maturing FPs for the same promoters is recommended to validate whether the dynamics observed reflect biological responses or FP artefacts.*

Response 2.6: As with the proteomics measurements, the reviewer also failed to note that we already did such comparisons of fluorescent reporters. In the various figures showing production and concentration traces we included different panels from reporters using different fluorescent proteins for the same promoter, and specifically noted this.

However, given that the reviewer missed this, we decided to re-organize these results in the revision. Whenever we have multiple reporters for the same protein, we now show only the results with our default GFPmut2 reporter. Instead, we added a figure (Fig S3) where we explicitly compare the protein production and concentration traces of different reporters, and show the effect of explicitly correcting for maturation. These results show that the effects of different maturation times (and also the effect of low copy number plasmid vs chromosomal reporter) are detectable but very moderate. Finally, in all figures that show results from reporters other than GFPmut2 we explicitly note this.

Comment 2.7: *Lack of Methodological Details in the Main Paper Limits Interpretation*

There is no dedicated Methods section, making it difficult to assess or replicate the work. Important missing details include:

Which reporters and FP variants were used (along with their known maturation times) Imaging settings (exposure, intervals, illumination intensity), needed to assess photobleaching and noise Calibration of OD sensors, fluidic switching protocols, and thresholds used for growth halt detection.

This information is essential for understanding the trade-offs in time resolution, photobleaching, and interpretability of the fluorescence data.

Response 2.7: Virtually all of this information was provided in the supplement of our manuscript. We specifically referred to particular sections and figures in this supplement in many locations in the manuscript, and are thus puzzled that the reviewer failed to take note of this.

Although the reviewer's criticisms are thus misplaced, we wanted to make sure that there can be no misunderstandings and for the revision we have included a specific 'Methods' section in the main text, in addition to a more extensive appendix with details of the methods, analysis procedures, and interpretation notes.

Comment 2.8: *Claimed Convergence of Abrupt and Gradual Starvation Is Not Fully Supported*

The authors report similar long-term transcriptional reporter dynamics between abruptly and gradually starved cells. However, this alone is insufficient to conclude similar physiological states or proteome outcomes.

A comparison of lag time distributions during resuscitation is necessary to support the claim

of functional equivalence. Previous studies, including work from Nathalie Balaban's lab, have shown that starvation dynamics crucially influence recovery kinetics.

Similarly, quantitative proteomic comparisons between the two modes of starvation would strengthen the conclusion about convergence.

Response 2.8: The reviewer clearly got the impression that we are making claims about the similarity between the two types of switches to carbon starvation (i.e. abrupt versus gradual loss of carbon source) that go beyond the observation that the gene expression response is similar. We did not intend to make any such claims in our manuscript, and don't believe we did. The word 'convergence' does not appear in our manuscript. We only claimed that we observe similar gene expression dynamics for the abrupt and more gradual starvation. We precisely quantified the similarity (and differences) of the responses in Figures 3, S14, S15, and S16 of the revision). These are experimental observations and we draw no further conclusions about physiological similarity.

We also do not say anything about lag times in the manuscript, let alone claiming that they are similar for the two stationary phases. We feel that a quantitative analysis of lag times and comparison of lag times across the two stationary states is beyond the scope of this manuscript (this would not only require a major analysis effort but also require new experiments).

In summary, we do not understand what 'claims of convergence' the reviewer is referring to. If the reviewer feels that we are making unsupported claims, (s)he would have to specify more concretely what claims in the manuscript they object to.

Nonetheless, in the revision we have taken pains to stress that we *only* claim similarity in the gene expression dynamics for these two modes of starvation.

Comment 2.9: *Use of Diverse Reporters Without Systematic Controls*

The authors use a variety of fluorescent reporters for different promoters, but the choice appears ad hoc. Since different FPs have different maturation and photobleaching rates, this introduces uncontrolled variation. To ensure reliability:

The same promoter should be tested with multiple FPs to isolate promoter dynamics from reporter artefacts. All promoter-FP pairs should be benchmarked against a common standard (e.g., sfGFP or YFP or mCherry) to allow cross-comparison.

Response 2.9: The controls that the reviewer claims were missing were in fact present. Results of the same promoter using different fluorescent proteins were listed in table S1 and Figs S4 and S5 of the original manuscript. As in previous comments, the reviewer failed to take note of the supplementary information (which is explicitly referred to all over the manuscript).

As explained in response 2.6, to make these comparisons more explicit, we now present comparisons of the same promoter with different reporters, including effects of correcting for maturation kinetics, together in Fig S3 of the revision.

We stress, however, that the large majority of results is based on a common reporter construct, so that even though there is some unavoidable variation across reporters (which we explicitly show), this does not materially affect our results and observations.

Comment 2.10: *Speculative Interpretations of Degradation and Resource Reallocation*

The manuscript suggests that protein degradation slows in starvation and that ribosome repression frees up resources for stress genes. However:

GFP signals do not reflect degradation dynamics, as these proteins resist native proteolysis. Translation and mRNA decay rates differ across genes and are not captured by the reporters. Such interpretations are speculative without direct measurements and should be clearly labeled as hypotheses.

Response 2.10: First, we agree with the fact that translation and mRNA decay rates *may* differ across genes and are not captured by the reporters. We stress this ourselves in the manuscript

However, we do not understand how the reviewer can claim that fluorescent proteins "do not reflect degradation dynamics, as these proteins resist native proteolysis". Our manuscript describes at length the control experiments that we performed to measure photobleaching rates and degradation rates of the different fluorescent proteins used. In addition, the manuscript specifically reports the degradation rates that we estimated from these experiments and explicitly pointed out that these rates are in line with previously reported overall protein degradation rates in *E. coli*. It is true that there may be some variability in degradation rate across proteins but we very clearly stress this limitation in the manuscript. In addition, we performed quantitative proteomics experiments to further investigate this and these also did not suggest that the GFP degradation is out of the ordinary. The reviewer's claim that fluorescent proteins are degraded exceptionally slowly (for which the reviewer provides no reference) are thus simply at odds with our measurements.

Given the reviewer's comments, we reviewed the relevant literature again, but have been unable to find a study measuring the degradation rates of both native and fluorescent proteins in bacteria. Quantitative measurements of fluorescent proteins degradation during stationary phase or starvation are scarce (which underscores the value of the degradation measurements we provide in our manuscript). The decay of GFPmut3 was measured to be "longer than 1 day" during the lag phase [11]. Assuming a half life of 2 days corresponds to a decay rate of 0.014/h, which is similar to the value we find. Regarding the differences between GFP variants, observations in *B. subtilis* indicate that the decay rate of GFPmut2 is twice as fast as for GFPmut3 [12], i.e. of the same order of magnitude. These numbers may seem low, but they are in fact close to estimated degradation rates for *native* proteins at low growth rates, i.e. around 0.035/h [13].

In summary, our measurements of degradation rates are well in line with results in the literature, and the degradation rates of fluorescent proteins are of the same order of magnitude as those estimated for native proteins.

Comment 2.11: *Inadequate Consideration of rpoS Dynamics*

The observation that rpoS-regulated genes are expressed before starvation is puzzling, given that RpoS is actively degraded in the exponential phase (half-life 30 s). The mechanism underlying pre-starvation expression requires clarification or supporting evidence.

Response 2.11: The reviewer expresses skepticism about our experimental observations that RpoS-regulated genes have non-zero expression during exponential phase based on the claim that RpoS is actively degraded and decays on a 30 second time scale (for which, again, no reference is provided). However, the observation that targets of RpoS are expressed during exponential growth is not just our experimental observation, there are many other datasets, both transcriptional reporters, transcriptomics (e.g. [2]), and proteomics (e.g. [14]), that confirm RpoS-regulated genes are indeed expressed during exponential phase. For the reviewer's reference, we prepared a figure showing the expression levels during growth and starvation of classically used RpoS reporters, bolA and blc [15], compared to the distribution of expression levels of all genes (data from [2]). This unequivocally shows that these RpoS-regulated genes are expressed during exponential growth (Fig. 1).

We also note that our experiments with transcriptional reporters measured in a ΔrpoS background indicate that the levels of RpoS reporters (e.g. bolA and med3 promoters) decrease not only during starvation, but also during growth (Fig. S13 in the revision).

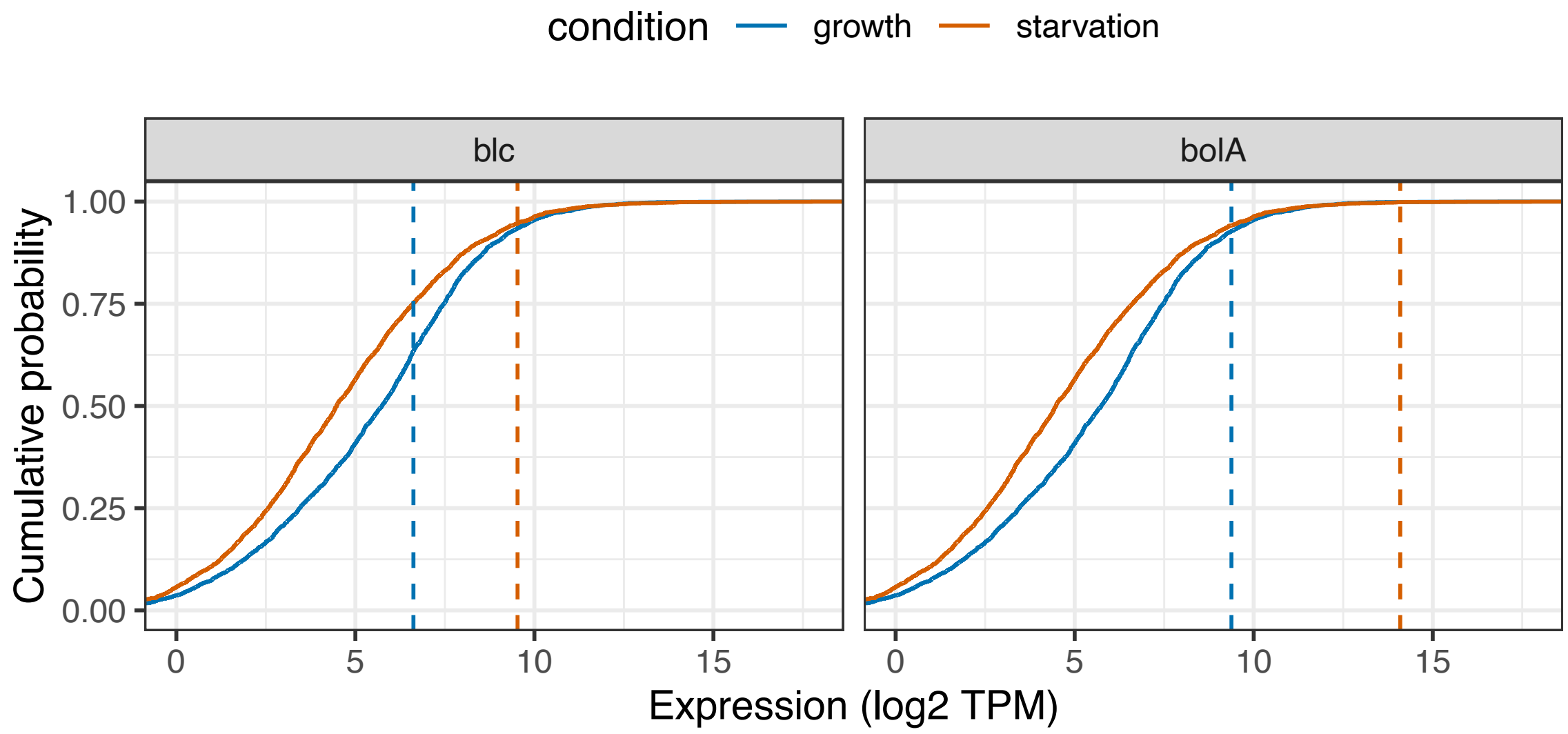

Figure 1: **RNAseq measurements of RpoS targets commonly used as reporters of RpoS activity.** In our revision, we used raw data from Lempp, et al. [2] to compute the TPM of each gene (see Appendix). Here we show the expression levels measured for blc and bolA during growth (blue dashed lines) and after 1 h in starvation (red dashed lines) against the background of the cumulative distribution of expression levels across all genes (blue and red curves). All technical replicates have been averaged for each gene and each time point. Note that both bolA and blc are among the top quartile of genes already in exponential phase, even though they are both clearly up-regulated during starvation.

Finally, the half-life of RpoS mentioned by the reviewer is one order of magnitude lower than the values we found in the literature (e.g. 4.6 min half-life of RpoS in Becker et al. [16]). We are frankly at a loss at how the reviewer can make so confidently claim facts that are clearly at odds with the existing literature.

Comment 2.12: *Minor Points*

Introduction: The claim that exponential phase is studied "because it is easy" misrepresents the rationale. It is used because it represents a reproducible, steady-state growth condition suitable for perturbation studies.

Response 2.12: We thank the reviewer for drawing our attention to the fact that this statement could be misunderstood, and have now rephrase the corresponding sentence.

Comment 2.13: *Literature: The statement that little is known about mortality during starvation ignores recent key studies: Biselli et al., mBio - showing longer survival with slower growth, Schink et al., eLife - modeling death rates under starvation. These should be cited and discussed in context.*

Response 2.13: We agree with the reviewer that references to the literature needed to be expanded here and have done so in the revision. We note, however, that different works report quite different mortality dynamics. We now highlight this as well in the revision, as detailed in Response 3.2.

Comment 2.14: *Figure 2b: The schematic of promoter classes is helpful, but it would benefit*

from hypothesized underlying mechanisms or regulatory motifs.

Response 2.14: We agree with the reviewer and have included such hypotheses in the revision.

Comment 2.15: *Mother machine implications: Since the device imposes no selective pressure and cells in gradual starvation arrest asynchronously, it would be useful to discuss how this affects the comparison with bulk measurements.*

Response 2.15: We agree that this could indeed introduce a systematic differences in the statistics for the mother machines and bulk populations. In the revision we have now included a calculation to estimate the size of this effect and find that the effect is so tiny that it is virtually indiscernible (see end of the section "Microfluidic experiments with batch culture overflow" in the Methods, and Appendix Fig S15).

Comment 2.16: *Terminology and clarity: Distinguish clearly between gene expression, protein abundance, and proteome-level changes throughout the text.*

Response 2.16: We agree that this is important and did attempt to clearly distinguish between these in our initial submission. However, for the revision we have carefully gone over the text again and made extra efforts to explain what our assay measures precisely, and to distinguish between these terms.

Comment 2.17: *Conclusion*

This paper presents a technically elegant and potentially valuable dataset on how E. coli responds to nutrient starvation at the single-cell level. However, the analysis of results ignores the considerations for the photophysics of the reporter proteins which can substantially affect the interpretations, and several key conclusions, particularly regarding proteome remodelling and convergence of starvation trajectories, are not sufficiently substantiated.

The study would benefit substantially from:

Reframing claims to reflect transcriptional dynamics rather than proteomic changes;

Incorporating maturation-aware modeling of fluorescence dynamics; Adding methodological transparency and systematic controls; e.g. Controls for similar FPs for different promoters and different FPs for the same promoters are needed to verify the results. Including additional functional comparisons (e.g., lag time, resuscitation dynamics) to validate claimed physiological similarities between abrupt and gradual starvation. Studying other growth media for general claims about the observed dynamics and their implications

Addressing these concerns will improve the clarity, rigor, and impact of the study.

Response 2.17:

This final comment mostly reiterates comments already made by this reviewer and our response will thus necessarily reiterate some of our earlier responses. First, while we agree that we did not pay enough attention to 'photophysics', i.e. the effects of maturation kinetics of fluorescent proteins, these do not substantially affect the interpretations at all. Our comparison of production dynamics for the same promoters with different fluorescent proteins in the original manuscript already confirmed that maturation dynamics do not dominate any of the observed time-dependent production dynamics. For the revision we now developed a method for explicitly correcting for maturation kinetics. In the revision we present a systematic comparison of results with and without maturation correction, confirming that maturation kinetics does not substantially affect our results.

Second, the controls that the reviewer asks for (same FP for different promoters and different FPs for same promoter) were in fact already done. The reviewer apparently failed to take note of them, despite the numerous references in the main text. Third, it is virtually inconceivable that the dramatic changes in expression of the transcriptional reporters would *not* be accompanied by

similarly substantial changes in the proteome. In the original manuscript we already provided analysis of proteomics data to support that the proteome is indeed substantially remodeled. In the revision we now explicitly show that the changes we observe with transcriptional reporters are consistent with proteomics data obtained by ourselves and others. In addition, we now also present a comparison with transcriptomics data, showing that these are also consistent with our observations. We of course agree that transcriptional reporters do not measure changes in mRNA stability and protein degradation across genes, and that therefore there are limitations to the interpretation of protein concentration fold-changes predicted by transcriptional reporters. We stress this ourselves in the manuscript and have further strengthened this in the revision. However, both proteomics and transcriptomics support the broad patterns of gene expression changes that we report.

Finally, we never made any claims of 'physiological similarities between abrupt and gradual starvation'. We merely pointed out the similarity of gene expression responses and never claimed any convergence in the physiology, mortality, or resuscitation of gradual and abrupt starvation. We have made sure to stress this even more in the revision. Since we make no claims of physiological 'convergence', we feel that the reviewer's request for additional experiments to investigate physiological similarity in terms of lag times and resuscitation dynamics are beyond the scope of our manuscript.

Detailed responses to the comments of reviewer #3

Comment 3.1: *Gervais et al. combine microfluidics with fluorescence microscopy to quantify gene expression in E. coli stationary phase. They show that protein production and degradation prepares cells for stress during the first 5-10 hours, which is important for stress survival. Importantly, they demonstrate at the single cell level that there is not a large heterogeneity in gene expression. These are interesting results, and the work could in principle be well suited for MSB. However, we have major concerns about the experimental methodology and inconsistencies with previous findings. Important control experiments are also missing.*

Response 3.1: We thank the reviewer for this summary. As the reviewer's concerns are only specified later in the review, we postpone our responses until the comments in which these concerns are specified.

Comment 3.2: *Major points*

1. It is important to understand to what degree bacteria in the experiment are starved. It has been observed consistently that E. coli and many other bacteria die when carbon runs out (see Yang et al., 2019 [DOI: 10.1126/sciadv.aaw2069], Reeve et al, 1984 [https://journals.asm.org/doi/epdf/10.1128/jb. 763.1984], Biselli et al 2020 [https://doi.org/10.15252/msb.20209478]). The experiments by Gervais et al. are more consistent with findings that bacteria survive (yet not grow) if there is a small amount of nutrient left in the medium (see e.g. Phaiboun et al. 2015, [https://doi.org/10.1371/journal.pcbi.1(Microfluidics can cause leaking nutrients into the medium, and a small amount of nutrients may strongly affect survival. Nevertheless, Yang et al. 2019 [DOI: 10.1126/sciadv.aaw2069] succeeded to show bacterial aging in microfluidics - in their experiments E. coli dies off by around 50% at 60h in starvation, which may not be consistent with these findings.

Therefore, the authors should perform control experiments to measure viability during the first 60h of starvation in batch, after performing a hard shift with washing with carbon free medium. If batch culture E. coli die substantially faster in batch after a hard shift than in the

mother machine for 60h, then we propose the authors remove the claim that they are measuring starvation and instead label their experiments as stationary phase (similar to previous work PMID: 24344288).

Response 3.2:

Based on our observed lack of mortality for 30-48 hours, the reviewer suggests that our cells might not be 'starving', citing works where cells were observed to start dying immediately after loss of nutrients. However, the reports in the literature on mortality dynamics are more complex than the reviewer paints them to be. There certainly are also studies that report a longer period before cells start to die, including in batch assays where it is unlikely that there is any carbon left, e.g Bergkessel & Delavaine [17]. In addition, results on mortality obtained by Yang and colleagues in microfluidics are less contradictory with ours than the reviewer reports. First the mortality in Yang, et al. 2019 is closer to 35% than to 50% after 60 h [18]. More importantly, in their subsequent study, the same authors report a much lower mortality of 10% after 60 h (using the same strain and media, MG1655 in M9 minimal media [19]), i.e. very similar to what we observe.

Most crucially, the only study measuring a survival curve in batch with the strain and media we used here reports that survival is very high during the first days of starvation, before it starts decaying exponentially [20]. In summary, the mortality dynamics we observe are fully consistent with other studies, including studies in batch cultures, and there is thus no reason to assume that our cells in M9 zero have considerably more trace levels of carbon available than in other experiments. However, we agree with the reviewer that it is important to stress this point and in the revision, we have now included more explicit citations to this literature.

With regards to the use of the term 'starvation', we note that given the size of Avogadro's number, carbon levels in the media are essentially a continuous quantity and will never be completely zero. It is not clear to us how one would decide how low carbon levels would have to be for it to be considered true 'starvation'. But that there is a critical 'low' carbon level below which cells go into growth arrest is a reproducible experimental fact: in all our experiments, *all* cells go into growth-arrest within 3 minutes of the switch from M9 glucose to M9 zero, and remain growth arrested until media with carbon return. Clearly, since the only change to the media is the removal of carbon, this consistent immediate growth arrest is due to the loss of carbon, and we feel it is completely appropriate to describe this response as carbon starvation.

Comment 3.3: *2. We have concerns that the illumination is affecting cellular physiology. Fig. S16A in the supporting Information appears to indicate that only half of the bacteria are able to regrow after 60h with illumination, but >90% without illumination?*

The remaining viable bacteria could also be affected by the illumination. Such stress could affect the bacteria's ability to synthesize proteins. If this is the case, the reader should be made aware of this potential limitation, and it should be discussed in the main text, possibly with additional control experiments testing the impact of this limitation. Such control experiments should show the difference between mother machine and batch culture without illumination and show the magnitude of the effect on protein-fold changes.

The batch control could either be proteomics or measurements using the authors' fluorescence reporters.

Measurements of the fluorescence reporters in bulk could be done by taking a sample prior to starvation and a sample at 60h into starvation and measuring single-cell fluorescence under the microscope. The authors may design their own experiments, but it is important that these experiments clarify the effect of the experimental setup on the measurements.

Response 3.3:

We fully understand the reviewer's concerns regarding possible phototoxicity effects. In fact, we had this on our radar screen during the project development, but we addressed it poorly in our initial manuscript.

For the revision, we have extended our discussion of phototoxicity and included a new figure (Appendix Fig S5) that shows:

1. Even with illumination, there is almost no mortality after 25h (95% ± 5% of the cells regrow in fresh media.

2. Without illumination, mortality is still low after 60h (92% ± 4% regrow).

3. With illumination, about half of the cells do not regrow after 60h starvation, indicating a clear phototoxicity effect late in starvation, which is also visible in the detectable shift of regrowth lag for the surviving cells.

We agree with the reviewer that we cannot exclude that the illumination could have some effects on gene expression. However, there are several reasons why we are confident that these effects must be minor:

1. There are no discernible effects of phototoxicity for 25 hours and virtually all expression changes occur much earlier, i.e. in the first 5-10 hours.

2. In the revision, we now show that the long-term fold-changes we observe are generally consistent with those observed in both transcriptomics and proteomics datasets (where there was no illumination), i.e. Appendix Figs S9 and S12.

3. There is a high correlation between gene expression early in starvation (where major phototoxicity effects are unlikely) and late in starvation (where we observed substantial phototoxicity effects), i.e. Fig. 5B.

In the revision we now explicitly point out that we are assuming that the illumination has no major effects on gene expression but that we cannot exclude some effects.

Comment 3.4: *3. In the 'soft shift' experiment a glucose batch culture is connected to the microfluidics. However, acetate is excreted to the medium during growth on glucose, which means that cells may not really be in starvation. While cells are not growing, acetate can be utilized as an energy source, which may affect phenotypes. This experiment may be fine as a characterization of a non-growing stationary phase state, but if the authors want to claim that cells are in starvation, this issue should be addressed.*

Response 3.4:

This is a good point and one that we indeed failed to sufficiently acknowledge in our original manuscript. As mentioned in a response to another reviewer, the main reason for performing the batch culture overflow experiments was to preempt concerns that our 'hard switch' was too artificial to represent gene expression responses that occur when nutrients run out in the wild.

We completely agree with the reviewer, however, that while in the M9 zero conditions there should virtually be no carbon source, in the batch overflow we do expect acetate to be present. If this presence of acetate were an important source of energy, one would thus expect large differences between the gene expression responses under these two switches.

But that is not what we see. In fact, the two responses are very similar (Fig 3 and Appendix Figs S14-S16). In addition, if we inhibit translation using chloramphenicol during the first 5 hours after the switch to M9 zero, we *still* see essentially the same response in RpoS targets once the chloramphenicol is removed (Fig S20). Thus, the picture that emerges from our experiments is that, when carbon availability drops sufficiently, cells go into growth-arrest and, rather than trying to rely on whatever low levels of nutrients might still be available in the environment, they initiate a tightly regulated gene expression program that is fueled by turnover of their own internal resources. In the revision we have added comments to make this point clear.

Comment 3.5: *Minor points*

1. Overall, the paper needs to embedded better into the scientific literature. The introduction and discussion fail to connect the work to past research on microbial starvation. Key topics are missing, such as prior work using microfluidics to study starvation and prior work on gene expression during starvation. The discrepancy with other works where bacteria die faster such as Yang et al. 2019 may need to be discussed.

Response 3.5:

We agree with the reviewer that the introduction and discussion could be improved in these respect. We have edited the introduction and in the revision now mention past research on microbial starvation, including work based on microfluidics, and also refer explicitly to prior work on gene expression during starvation. In addition we added a paragraph to the discussion to explain discrepancies between studies regarding *E. coli* mortality during starvation.

References

[1] Luca Galbusera, Gwendoline Bellement-Theroue, Thomas Julou, and Erik van Nimwegen. Rapid transcription factor fluctuations drive nonequilibrium gene regulatory dynamics in bacteria. *PRX Life*, 3(3):033006, July 2025.

[2] Martin Lempp, Niklas Farke, Michelle Kuntz, Sven Andreas Freibert, Roland Lill, and Hannes Link. Systematic identification of metabolites controlling gene expression in *E. coli*. *Nature Communications*, 10(1):4463, October 2019.

[3] Orit Gefen, Ofer Fridman, Irine Ronin, and Nathalie Q. Balaban. Direct observation of single stationary-phase bacteria reveals a surprisingly long period of constant protein production activity. *Proceedings of the National Academy of Sciences of the United States of America*, 111(1):556–561, January 2014.

[4] Somenath Bakshi, Emanuele Leoncini, Charles Baker, Silvia J. Cañas-Duarte, Burak Okumus, and Johan Paulsson. Tracking bacterial lineages in complex and dynamic environments with applications for growth control and persistence. *Nature Microbiology*, 6(6):783–791, June 2021.

[5] Matthias Kaiser, Florian Jug, Thomas Julou, Siddharth Deshpande, Thomas Pfohl, Olin K. Silander, Gene Myers, and Erik van Nimwegen. Monitoring single-cell gene regulation under dynamically controllable conditions with integrated microfluidics and software. *Nature Communications*, 9(1):212, January 2018.

[6] Guillaume Witz, Erik van Nimwegen, and Thomas Julou. Initiation of chromosome replication controls both division and replication cycles in *E. coli* through a double-adder mechanism. *eLife*, 8:e48063, nov 2019.

[7] Thomas Julou, Ludovit Zweifel, Diana Blank, Athos Fiori, and Erik van Nimwegen. Subpopulations of sensorless bacteria drive fitness in fluctuating environments. *PLoS Biology*, 18(12):e3000952, December 2020. Number: 12.

[8] Rohan Balakrishnan, Matteo Mori, Igor Segota, Zhongge Zhang, Ruedi Aebersold, Christina Ludwig, and Terence Hwa. Principles of gene regulation quantitatively connect DNA to RNA and proteins in bacteria. *Science (New York, N.Y.)*, 378(6624):eabk2066, December 2022.

[9] Alon Zaslaver, Anat Bren, Michal Ronen, Shalev Itzkovitz, Ilya Kikoin, Seagull Shavit, Wolfram Liebermeister, Michael G. Surette, and Uri Alon. A comprehensive library of fluorescent transcriptional reporters for *Escherichia coli*. *Nature Methods*, 3(8):623–628, August 2006.

[10] Enrique Balleza, J. Mark Kim, and Philippe Cluzel. Systematic characterization of maturation time of fluorescent proteins in living cells. *Nature methods*, 15(1):47–51, January 2018.

[11] J B Andersen, C Sternberg, L K Poulsen, S P Bjorn, M Givskov, and S Molin. New unstable variants of green fluorescent protein for studies of transient gene expression in bacteria. *Appl. Environ. Microbiol.*, 64(6):2240–2246, June 1998.

[12] Alex Rosenberg, Lior Sinai, Yoav Smith, and Sigal Ben-Yehuda. Dynamic expression of the translational machinery during bacillus subtilis life cycle at a single cell level. *PLoS One*, 7(7):e41921, July 2012.

[13] Ludovico Calabrese, Jacopo Grilli, Matteo Osella, Christopher P. Kempes, Marco Cosentino Lagomarsino, and Luca Ciandrini. Protein degradation sets the fraction of active ribosomes at vanishing growth. *PLoS Computational Biology*, 18(5):e1010059, May 2022.

[14] Alexander Schmidt, Karl Kochanowski, Silke Vedelaar, Erik Ahrné, Benjamin Volkmer, Luciano Callipo, Kèvin Knoops, Manuel Bauer, Ruedi Aebersold, and Matthias Heinemann. The quantitative and condition-dependent *Escherichia coli* proteome. *Nature Biotechnology*, 34(1):104–110, January 2016.

[15] Om Patange, Christian Schwall, Matt Jones, Casandra Villava, Douglas A. Griffith, Andrew Phillips, and James C. W. Locke. Escherichia coli can survive stress by noisy growth modulation. *Nature Communications*, 9(1):5333, December 2018.

[16] G Becker, E Klauck, and R Hengge-Aronis. Regulation of RpoS proteolysis in escherichia coli: the response regulator RssB is a recognition factor that interacts with the turnover element in RpoS. *Proc. Natl. Acad. Sci. U. S. A.*, 96(11):6439–6444, May 1999.

[17] Megan Bergkessel and Laurent Delavaine. Diversity in starvation survival strategies and outcomes among heterotrophic proteobacteria. *Microbial Physiology*, 31(2):146–162, 2021.

[18] Yifan Yang, Ana L. Santos, Luping Xu, Chantal Lotton, François Taddei, and Ariel B. Lindner. Temporal scaling of aging as an adaptive strategy of *Escherichia coli*. *Science Advances*, 5(5):eaaw2069, May 2019.

[19] Yifan Yang, Omer Karin, Avi Mayo, Xiaohu Song, Peipei Chen, Ana L. Santos, Ariel B. Lindner, and Uri Alon. Damage dynamics and the role of chance in the timing of E. coli cell death. *Nature Communications*, 14(1):2209, April 2023.

[20] S. Dukan and T. Nyström. Oxidative stress defense and deterioration of growth-arrested Escherichia coli cells. *The Journal of Biological Chemistry*, 274(37):26027–26032, September 1999.

31st Mar 2026

Manuscript Number: MSB-2025-13152R
Title: E. coli prepares for starvation by dramatically remodeling its proteome in the first hours after loss of nutrients

Dear Dr Julou,

Thank you again for submitting your revised work to Molecular Systems Biology. We have now heard back from two of the original three reviewers who evaluated your study. As you will see below, the reviewers are supportive on the value of the datasets and manuscript; however Reviewer 2 continues to have concerns on how some comments of theirs and Reviewer 3's were only partially addressed, mainly which pertain to conclusions that can be drawn with the experimental datasets. We would therefore ask you to address their concerns in a revision. In particular it will be necessary to tone down the conclusions and discuss the limitations for concerns that were not experimentally addressed. Please let me know in case you would like to discuss in further detail any of the any of the reviewer comments, I would be happy to schedule a call.

We remind you that we have the following formatting requirements:
-
1) A .docx formatted version of the manuscript text (including legends for main figures, EV figures and tables). Please make sure that the changes are highlighted to be clearly visible. Alternatively you may choose to submit your manuscript as a LaTeX file.

2) Individual production quality figure files as .eps, .tif, .jpg (one file per figure). For guidance, download the 'Figure Guide PDF' (https://media.springernature.com/original/springer-cms/rest/v1/content/27825798/data/v1).

3) At EMBO Press we ask authors to provide source data for the main figures. Our source data coordinator will contact you to discuss which figure panels we would need source data for and will also provide you with helpful tips on how to upload and organize the files.

4) A .docx formatted letter INCLUDING the reviewers' reports and your detailed point-by-point responses to their comments. As part of the EMBO Press transparent editorial process, the point-by-point response is part of the Peer Review File (PRF), which will be published alongside your paper.

5) A complete author checklist, which you can download from our author guidelines (https://link.springer.com/journal/44320/submission-guidelines#cms). Please insert information in the checklist that is also reflected in the manuscript. The completed author checklist will also be part of the PRF.

6) Please note that all corresponding authors are required to supply an ORCID ID for their name upon submission of a revised manuscript.

7) It is mandatory to include a 'Data Availability' section after the Materials and Methods. Before submitting your revision, primary datasets produced in this study need to be deposited in an appropriate public database, and the accession numbers and database listed under 'Data Availability'. Please remember to provide a reviewer password if the datasets are not yet public (see https://link.springer.com/partners/embo-press/editorial-policies#Data%20availability%20statement).

In case you have no data that requires deposition in a public database, please state so in this section as follows: "This study includes no data deposited in external repositories". Note that the Data Availability Section is restricted to new primary data that are part of this study.

8) All Materials and Methods need to be described in the main text using our 'Structured Methods' format, which is required for all research articles. According to this format, the Methods section includes a Reagents and Tools Table (listing key reagents, experimental models, software and relevant equipment and including their sources and relevant identifiers) followed by a Methods and Protocols section describing the methods using a step-by-step protocol format. The aim is to facilitate adoption of the methodologies across labs. Please upload the Reagents and Tools table as a separate document when submitting your revised manuscript. More information on how to adhere to this format as well as a downloadable template (.docx) for the Reagents and Tools Table can be found in our author guidelines: https://link.springer.com/journal/44320/submission-guidelines#cms

9) For data quantification: please specify the name of the statistical test used to generate error bars and p-values, the number (n) of independent experiments (specify technical or biological replicates) underlying each data point and the test used to calculate p-values in each figure legend. The figure legends should contain a basic description of n, p-values and the test applied. Graphs must include a description of the bars and the error bars (s.d., s.e.m.). Please provide exact p-values (in either the figure or figure legend).

10) Our journal encourages inclusion of *data citations in the reference list* to directly cite datasets that were re-used and obtained from public databases. Data citations in the article text are distinct from normal bibliographical citations and should directly link to the database records from which the data can be accessed. In the main text, data citations are formatted as follows: "Data ref: Smith et al, 2001" or "Data ref: NCBI Sequence Read Archive PRJNA342805, 2017". In the Reference list, data citations must be labeled with "[DATASET]". A data reference must provide the database name, accession number/identifiers and a resolvable link to the landing page from which the data can be accessed at the end of the reference. Further instructions are available at .

11) We replaced Supplementary Information with Expanded View (EV) Figures and Tables that are collapsible/expandable online. EV Figures should be cited as 'Figure EV1, Figure EV2" etc... in the text and their respective legends should be included in the main text after the legends of regular figures.
- For the figures that you do NOT wish to display as Expanded View figures, they should be bundled together with their legends in a single PDF file called *Appendix*, which should start with a short Table of Content. Appendix figures should be referred to in the main text as: "Appendix Figure S1, Appendix Figure S2" etc.
- Additional Tables/Datasets should be labeled and referred to as Table EV1, Dataset EV1, etc. Legends should be provided in a separate tab in case of .xls files. Alternatively, the legend can be supplied as a separate text file (README) and zipped together with the Table/Dataset file.
See detailed instructions here:
https://link.springer.com/journal/44320/submission-guidelines#cms

12) Author contributions: CRediT has replaced the traditional author contributions section because it offers a systematic machine-readable author contributions format that allows for more effective research assessment. Please remove the Authors Contributions from the manuscript and use the free text boxes beneath each contributing author's name in our system to add specific details on the author's contribution. More information is available in our guide to authors.

13) Disclosure statement and competing interests: We updated our journal's competing interests policy in January 2022 and request authors to consider both actual and perceived competing interests. Please review the policy https://link.springer.com/partners/embo-press/editorial-policies#Competing%20interest%20disclosures and update your competing interests if necessary.

14) Every published paper now includes a 'Synopsis' to further enhance discoverability. Synopses are displayed on the journal webpage and are freely accessible to all readers. They include a short stand first (maximum of 300 characters, including space) as well as 2-5 one-sentences bullet points that summarizes the paper. Please write the bullet points to summarize the key NEW findings. They should be designed to be complementary to the abstract - i.e. not repeat the same text. We encourage inclusion of key acronyms and quantitative information (maximum of 30 words / bullet point). Please use the passive voice. Please attach these in a separate file or send them by email, we will incorporate them accordingly.

Please also suggest a striking image or visual abstract to illustrate your article as a PNG file 550 px wide x 300 px high.

Please note that these would be the final versions and changes during proofing are usually not allowed.

15) As part of the EMBO Publications transparent editorial process initiative (see our policy here: https://link.springer.com/partners/embo-press/editorial-policies#Peer%20review), Molecular Systems Biology will publish online a Peer Review File (PRF) to accompany accepted manuscripts.
In the event of acceptance, this file will be published in conjunction with your paper and will include the anonymous referee reports, your point-by-point response and all pertinent correspondence relating to the manuscript. Let us know whether you agree with the publication of the PRF and as here, if you want to remove or not any figures from it prior to publication.
Please note that the Author checklist will be published at the end of the PRF.

Molecular Systems Biology has a "scooping protection" policy, whereby similar findings that are published by others during review or revision are not a criterion for rejection. Should you decide to submit a revised version, I do ask that you get in touch after three months if you have not completed it, to update us on the status.

Yours sincerely,

Poonam Bheda, PhD
Scientific Editor
Molecular Systems Biology

Reviewer #1:

As I said in my previous report I think this is a nice and interesting work and the manuscript is clear and well organized. The revisions made it even clearer and I recommend its publication as it is

I thank the authors for having addressed my comments.

Reviewer #2:

Comments - Gervais et al

I have carefully evaluated the revised manuscript together with the authors' responses. The study presents a technically strong and valuable dataset on single-cell gene expression dynamics during carbon starvation. I appreciate the authors' efforts in revision, particularly the incorporation of maturation-aware analysis, improved presentation of reporter comparisons, and inclusion of a Methods section, which together strengthen the clarity and technical robustness of the work.

At the same time, my main remaining concern relates to the scope of several conclusions relative to the measurements presented. The study relies on transcriptional reporter data combined with model-based inference, yet some interpretations, particularly those concerning proteome remodelling, resource allocation, and broader adaptive mechanisms, are framed at a level that appears to extend beyond what can be directly supported. I believe the manuscript would be further strengthened by consistently aligning the strength of its conclusions with the scope of the underlying measurements.

Responses to authors' replies and revisions

Response to Comment 2.1

Thank you for the clarification.

The summary provided in Comment 2.1 was intended as a concise overview of the study's scope, aims, and primary conclusions, rather than a comprehensive enumeration of all findings. No downplaying of specific results was intended.

Regarding the comparison between abrupt and gradual starvation, I appreciate the authors' clarification that their claims are restricted to similarities in gene expression dynamics and do not extend to broader adaptive or survival outcomes. If this distinction is central to the interpretation of the work, I agree that ensuring this limitation is stated clearly and consistently throughout the manuscript is important to avoid potential misinterpretation by readers.

Response to Comment 2.2

Thank you for the clarification. My general remarks were intended to evaluate whether the conclusions are fully supported by the scope of the data and analyses presented. The concern is not primarily about the absence of specific controls, but rather about the strength of the interpretations relative to what is directly measured or inferred.

In particular, several conclusions, such as those relating to proteome remodelling, the equivalence of abrupt and gradual starvation responses, the accuracy of inferred expression dynamics, and the predictive or causal role of early expression, appeared to extend beyond what can be unambiguously supported by transcriptional reporter measurements and model-based inference alone.

If the authors have included additional controls or analyses that address these points (e.g. proteomics measurements or comparisons across fluorescent reporters), it would be helpful for the manuscript to more explicitly demonstrate how these results substantiate the corresponding claims. At present, the connection between the data and some of the broader interpretations presented in the results and their discussion is not yet clearly established.

The specific comments below are intended to clarify these points and suggest ways to strengthen the linkage between data and conclusions.

Response to Comment 2.3

Thank you for the clarification. The intent of this comment was to provide a high-level contextualisation of the work, rather than to catalogue all individual results. No omission was intended to diminish specific findings presented in the manuscript.

I agree that the study brings together several valuable elements, particularly the systematic and time-resolved tracking of

transcriptional responses across multiple promoters at the single-cell level. This combination of measurements and analysis is a clear strength of the work.

At the same time, my comments aimed to place these contributions in the context of prior studies. Several aspects, such as the use of multi lane mother-machine microfluidics and under changing conditions (in papers cited in this manuscript), single-cell lineage tracking and inference of fluorescence time series (A Pavlou et al 2025), and observations related to growth arrest and reductive division (T Shimaya et al 2021), have been explored in earlier work. In this light, the advance here appears to lie primarily in the integration, scale, and quantitative analysis of these measurements across multiple promoters and transition modes between conditions.

My suggestion was therefore that the manuscript could more clearly articulate this positioning, distinguishing between what is newly enabled in this study and what builds on established approaches. This would help sharpen the conceptual contribution and make the advance more transparent to readers.

The point regarding the strength of claims related to proteome remodelling and interpretation of starvation responses remains a separate issue concerning how conclusions are framed relative to the data, which is addressed in the detailed comments below.

Response to Comment 2.4
Thank you for the clarification. The concern raised here is not that transcriptional changes are unrelated to protein levels, but rather that the strength of the conclusions regarding proteome remodelling appears to extend beyond what can be directly supported by transcriptional reporter measurements and the associated model-based inference.

While it is well established that transcription contributes to shaping protein abundances, protein levels in starvation conditions are also influenced by additional factors, including translation efficiency, protein stability, and degradation dynamics. These factors become particularly relevant in non-growing or energy-limited states, where turnover and resource constraints can substantially affect protein concentrations.

With regard to the proteomics measurements, the manuscript notes that these data are not sufficiently reproducible to provide reliable quantitative estimates of fold-changes in individual proteins. In this context, it is unclear to what extent they can serve as a strong validation of correspondence between reporter-based measurements and proteome-level dynamics. The reported qualitative agreement or overlap in trends, while suggestive, does not fully resolve this limitation.

The suggestion was therefore to more carefully qualify the use of the term proteome remodelling, or to frame the conclusions more explicitly in terms of transcriptional or reporter-based dynamics, unless stronger direct evidence of proteome-level changes is provided.

Response to Comment 2.5

Thank you for addressing this point and for incorporating fluorescent protein maturation into the revised analysis.

I appreciate the clarification regarding the use of GFPmut2 and the explicit correction applied to mVenus and mCherry reporters. The updated analysis accounting for maturation kinetics, along with the reference to Balleza et al. (2018), satisfactorily addresses the concern raised.

Response to Comment 2.6

Thank you for the clarification and for reorganising these results.

The inclusion of a dedicated comparison between reporters with different maturation kinetics (Fig. S3), together with the explicit correction for maturation effects, provides a useful validation of the reported dynamics. Presenting these comparisons more clearly strengthens the analysis.

Response to Comment 2.7

Thank you for adding a dedicated Methods section to the main text.

The intent of this comment was not to suggest that the information was entirely absent, but rather that its placement primarily in the supplementary material made it difficult to assess key methodological aspects and their implications for data interpretation. The inclusion of a Methods section in the main manuscript significantly improves clarity and accessibility.

Response to Comment 2.8

I appreciate that the authors intend to restrict their conclusions to similarities in gene expression dynamics between abrupt and gradual starvation. The concern raised here was primarily about how these results may be interpreted by readers, particularly in the context of broader discussion of adaptation mechanisms.

Even when explicitly limited to transcriptional dynamics, similarities in reporter behaviour can be interpreted as implying a degree of functional or physiological equivalence, especially when these observations are discussed in the context of general adaptive responses to starvation. The suggestion was therefore to ensure that the manuscript clearly and consistently delineates the scope of this comparison and avoids potential overinterpretation beyond gene expression dynamics.

Response to Comment 2.9

Thank you for the clarification and for reorganising these comparisons. The intent of this comment was not to suggest that such controls were entirely absent, but rather that the use of multiple reporters with different properties was not presented in a sufficiently systematic way to clearly assess their impact on the reported dynamics. The inclusion of a consolidated comparison (Fig. S3), together with explicit correction for maturation kinetics, significantly improves the clarity of this aspect of the analysis.

Response to Comment 2.10

Thank you for the clarification regarding the measurements of fluorescent protein degradation and their comparison to literature values.

The primary concern raised here is not limited to the absolute degradation rate of fluorescent proteins, but rather the extent to which reporter-based measurements can support interpretations about protein turnover and resource allocation at the proteome level. Even if fluorescent protein degradation rates are of the same order of magnitude as native proteins on average, fluorescent proteins are generally relatively stable and are not subject to the same diversity of regulated degradation mechanisms that apply to native proteins.

As a result, reporter measurements do not capture potential gene-specific differences in degradation, translation efficiency, or mRNA dynamics. In particular, extrapolations from reporter-based degradation measurements to proteome-wide behaviour, and to quantitative estimates of resource availability, rely on assumptions that are not directly validated by the presented data.

The suggestion was therefore that interpretations linking the observed dynamics to mechanisms such as resource reallocation, endogenous metabolism, or proteolysis-driven energy supply would benefit from being clearly framed as hypotheses or plausible explanations, rather than as conclusions directly established by the data.

Response to Comment 2.11

Thank you for the clarification and for providing additional evidence regarding the expression of RpoS-regulated genes during exponential growth.

The intent of this comment was to express surprise in light of the established literature showing rapid RpoS turnover during exponential phase, and therefore to highlight that the observed pre-starvation expression raises a mechanistic question. I did not mean to suggest that expression of these promoters in the exponential phase is impossible, but rather that it would benefit from clarification.

In particular, it would be helpful to discuss whether this signal reflects basal RpoS activity despite low steady-state levels, contributions from other regulatory inputs or sigma factors, or possible background effects in the fluorescence readout, such as cellular autofluorescence. Explicitly acknowledging these possibilities would strengthen the interpretation of these observations.

Response to Comment 2.17

I appreciate the efforts made to address several of the points raised, in particular the incorporation of maturation-aware analysis of fluorescence dynamics, the clearer presentation of comparisons across different reporters, and the inclusion of a Methods section in the main text. These changes improve the clarity and technical robustness of the manuscript.

The remaining concern relates primarily to the strength and scope of the conclusions relative to the data presented. In particular, interpretations concerning proteome remodelling, resource allocation, and broader adaptive mechanisms appear to extend beyond what can be directly supported by transcriptional reporter measurements and model-based inference alone. While the additional comparisons to proteomics and transcriptomics are useful for establishing qualitative consistency, they do not fully resolve these limitations at the level of quantitative or mechanistic interpretation.

Similarly, although the manuscript now clarifies that the comparison between abrupt and gradual starvation is limited to gene expression dynamics, care in framing remains important to avoid potential overinterpretation in terms of broader physiological

equivalence.

Overall, I believe the manuscript presents a valuable and carefully executed dataset, and would be strengthened further by consistently aligning the framing of conclusions with the scope of the measurements.

****

I found that several of Reviewer 3's comments raise important experimental and interpretational questions that, in my view, are not fully addressed in the current responses. I summarise a few key points below that may be helpful in guiding further revision.

Response to comment 3.2 (degree of starvation / nutrient carryover in microfluidics):
The reviewer's concern appears to be whether the microfluidic setup allows a sufficiently clean transition to nutrient-free conditions. In bulk experiments, done in the cited studies, cells are typically centrifuged, washed, and resuspended in fresh medium, which provides a relatively well-controlled removal of residual nutrients. In contrast, in microfluidic devices, cells reside in confined geometries (e.g. trenches), and residual medium may persist due to incomplete exchange or diffusion from surrounding materials (e.g. PDMS), potentially leading to trace nutrient availability over extended times.

The response focuses on variability in mortality reported in the literature and on the definition of "starvation," but does not directly address this physical aspect of the system. A more direct assessment (e.g. of media exchange efficiency, or controls comparing batch and device conditions under a hard switch) or a clearer discussion of this limitation would strengthen the interpretation.

Response to comment 3.3 (phototoxicity):
The revised analysis indicates a substantial phototoxicity effect at longer times (e.g. reduced regrowth after 60 h with illumination). While the authors argue that early dynamics are unaffected, the reviewer's concern is broader-namely, whether illumination may influence physiological state and mortality. The current phrasing ("we cannot exclude some effects") seems weaker than warranted given that measurable effects on viability are already observed. A more explicit acknowledgement of the magnitude of phototoxicity and its potential impact on interpretation would be appropriate.

Response to comment 3.4 (soft shift and residual carbon sources such as acetate):
The reviewer raises a valid point that overflow from batch culture likely contains alternative carbon sources (e.g. acetate), as well as other changes associated with gradual nutrient depletion (e.g. pH shifts, accumulation of by-products). These factors can influence cellular physiology even in the absence of growth.

The response emphasises the similarity of gene expression dynamics between hard and soft shifts and proposes a model in which adaptation is driven primarily by internal resource turnover. However, this interpretation does not directly rule out contributions from residual external nutrients or environmental changes present in the soft-shift condition.

A more direct test, for example, introducing defined low concentrations of carbon sources in a controlled manner, or more explicitly discussing the limitations of the comparison, would help clarify the extent to which the observed dynamics are intrinsic versus environmentally driven.

More generally, across these responses, there is a recurring pattern in which concerns about experimental interpretation are addressed by comparison to literature or by reframing the question, rather than by directly engaging with the specific experimental limitations raised. Clarifying these points would, in my view, significantly improve the rigor and interpretability of the manuscript.

Detailed responses to the comments of reviewer #1

Comment 1.1: *As I said in my previous report I think this is a nice and interesting work and the manuscript is clear and well organized. The revisions made it even clearer and I recommend its publication as it is*

I thank the authors for having addressed my comments.

Response 1.1: We are delighted the reviewer appreciated our revisions and thank them for their constructive comments.

Detailed responses to the comments of reviewer #2

Comment 2.0: ***Comments - Gervais et al***

I have carefully evaluated the revised manuscript together with the authors' responses. The study presents a technically strong and valuable dataset on single-cell gene expression dynamics during carbon starvation. I appreciate the authors' efforts in revision, particularly the incorporation of maturation-aware analysis, improved presentation of reporter comparisons, and inclusion of a Methods section, which together strengthen the clarity and technical robustness of the work.

At the same time, my main remaining concern relates to the scope of several conclusions relative to the measurements presented. The study relies on transcriptional reporter data combined with model-based inference, yet some interpretations, particularly those concerning proteome remodelling, resource allocation, and broader adaptive mechanisms, are framed at a level that appears to extend beyond what can be directly supported. I believe the manuscript would be further strengthened by consistently aligning the strength of its conclusions with the scope of the underlying measurements.

Response 2.0:

We thank the reviewer for acknowledging that our revision addressed many of their concerns. Regarding the remaining expression of concern, we of course agree that the scope of our conclusions should be commensurate with our results. However, the topics of the reviewer's concerns appear to be the same as concerns already expressed in their first round of reviews and we feel that, already in our first revision, we took great care to ensure that the scope of our conclusions matches the evidence in our results. Nonetheless, in this second round of revision, we have gone over all the relevant sections in the manuscript and have updated the phrasing in a number of places to make sure our interpretations and conclusions stay firmly within the scope of our results.

To comment specifically on the topics regarding which the reviewer expresses concern:

- We use the time-dependent protein production rates, as measured by our transcriptional reporters, in combination with protein degradation and bleaching measurements, to infer protein concentration changes and we very explicitly acknowledge that, in order for the inferred protein concentration changes to apply to native proteins, we have to make several assumptions (e.g. that degradation rates of native proteins are similar to those of the fluorescent reporter proteins). To validate these assumptions we use proteomics measurements from ourselves and multiple other studies, as well as existing transcriptomic data, and find that the overall picture provided by the transcriptome and proteomics measurements match what we infer from our transcriptional reporters. Below we illustrate this with quotes from our revision that demonstrate the care we took in our formulations.

- Regarding resource allocation, our results firmly establish that there is very substantial gene expression and proteolysis occurring in the first 5-10 hours after the loss of carbon source, that these are similar between an abrupt and more gradual switch, that this gene expression is crucial for stress resistance later in stationary phase, and that if protein production is inhibited during the first hours of starvation with chloramphenicol, a similar gene expression response is *still* observed after the chloramphenicol is removed. It is only in the Discussion section that we present some of our proposed interpretations of these observations for resource allocation, e.g. that we believe that it is likely that the cells fuel this gene expression program during starvation from the resources obtained by proteolysis. We feel it is entirely appropriate to present such interpretations in the Discussion section.
- We assume that the reviewer's phrase 'broader adaptive mechanisms' is meant to refer to our observations that the gene expression responses under abrupt vs gradual entry into starvation are very similar. Here too, we paid specific attention in the revision to phrase the description of our results to apply *only* to the similarity of the gene expression responses, and avoided concluding that the responses must be similar in all physiological aspects.

It is unfortunate that the reviewer does not point to any specific statements in our revision that they feel are problematic (in fact, the reviewer is strictly speaking not even saying that there *are* problematic statements left, but merely claims that there are statements that *appear* to extend beyond what can be directly supported). In the absence of concrete examples of statements that the reviewer feels are problematic,it is difficult to respond to this expression of concern.

As mentioned already, we have carefully gone through all the relevant sections in the manuscript and ensured the phrasings, interpretations, and conclusions, all remain within what is directly supported by our results.

Response to Comment 2.1: *Thank you for the clarification.*

The summary provided in Comment 2.1 was intended as a concise overview of the study's scope, aims, and primary conclusions, rather than a comprehensive enumeration of all findings. No downplaying of specific results was intended.

Regarding the comparison between abrupt and gradual starvation, I appreciate the authors' clarification that their claims are restricted to similarities in gene expression dynamics and do not extend to broader adaptive or survival outcomes. If this distinction is central to the interpretation of the work, I agree that ensuring this limitation is stated clearly and consistently throughout the manuscript is important to avoid potential misinterpretation by readers.

Second Response 2.1: Regarding the reviewer leaving out results in their summary, we will only note that the reviewer still nowhere acknowledges our experiments demonstrating the dramatic effects on stress resistance of the gene expression early in stationary phase (which, crucially, unambiguously prove the major physiological consequences of this expression program).

We will comment more extensively on the comparison between abrupt and gradual starvation in Comment 2.8. We note, however, that while it is indeed important to make clear that our results only show the similarity in gene expression changes under abrupt and gradual starvation, and that we haven't directly demonstrated that other physiological aspects are also the same, there is also no reason to assume that the physiological responses will be different. In fact, arguably the simplest assumption is that other physiological aspects will also be the same.

Response to Comment 2.2: *Thank you for the clarification. My general remarks were intended to evaluate whether the conclusions are fully supported by the scope of the data and analyses presented. The concern is not primarily about the absence of specific controls, but rather about the strength of the interpretations relative to what is directly measured or inferred.*

In particular, several conclusions, such as those relating to proteome remodelling, the equivalence of abrupt and gradual starvation responses, the accuracy of inferred expression dynamics, and the predictive or causal role of early expression, appeared to extend beyond what can be unambiguously supported by transcriptional reporter measurements and model-based inference alone.

If the authors have included additional controls or analyses that address these points (e.g. proteomics measurements or comparisons across fluorescent reporters), it would be helpful for the manuscript to more explicitly demonstrate how these results substantiate the corresponding claims. At present, the connection between the data and some of the broader interpretations presented in the results and their discussion is not yet clearly established.

The specific comments below are intended to clarify these points and suggest ways to strengthen the linkage between data and conclusions.

Second Response 2.2:

As the reviewer is mostly commenting on their own previous comment from the first round of reviews, it is difficult to tell whether the reviewer feels further changes are required, i.e. the reviewer says 'several conclusions... *appeared* to extend beyond...', i.e. past tense (emphasis ours).

The phrasing in the second paragraph unfortunately again creates the impression that we have not done proteomics measurements or comparisons across fluorescent reporters. Whereas, in fact, we did perform proteomics measurements. We did explicit comparisons across fluorescent reporters. We described all these in the original manuscript. We further stressed this in our revision and discussed explicitly how they support results from our transcriptional reporters. In the revision we also added a careful comparison with transcriptomics results, which further strengthen our conclusions. And we pointed all this out in our response to the previous review (where this reviewer had wrongly claimed we had not performed proteomics or comparisons across fluorescent reporters).

Below is a list of locations in the manuscript where these issues are discussed:

- Validation with proteomics: lines 382-400.
- Consistency with transcriptomics measurements: lines 320-327.
- Comparison of different fluorescent reporters: line 224-232 and in particular Fig S3.

Response to Comment 2.3: *Thank you for the clarification. The intent of this comment was to provide a high-level contextualisation of the work, rather than to catalogue all individual results. No omission was intended to diminish specific findings presented in the manuscript.*

I agree that the study brings together several valuable elements, particularly the systematic and time-resolved tracking of transcriptional responses across multiple promoters at the single-cell level. This combination of measurements and analysis is a clear strength of the work.

At the same time, my comments aimed to place these contributions in the context of prior studies. Several aspects, such as the use of multi lane mother-machine microfluidics and under changing conditions (in papers cited in this manuscript), single-cell lineage tracking and inference

of fluorescence time series (A Pavlou et al 2025), and observations related to growth arrest and reductive division (T Shimaya et al 2021), have been explored in earlier work. In this light, the advance here appears to lie primarily in the integration, scale, and quantitative analysis of these measurements across multiple promoters and transition modes between conditions.

My suggestion was therefore that the manuscript could more clearly articulate this positioning, distinguishing between what is newly enabled in this study and what builds on established approaches. This would help sharpen the conceptual contribution and make the advance more transparent to readers.

The point regarding the strength of claims related to proteome remodelling and interpretation of starvation responses remains a separate issue concerning how conclusions are framed relative to the data, which is addressed in the detailed comments below.

Second Response 2.3:

As in previous comments, the reviewer appears to mainly explain their comment in the first round of review, which indeed asked to better place our contributions in the context of prior studies, in particular the works of Schink, et al. and of Moreno-Gomez, et al. We feel that we successfully addressed this request in our revision. In particular, we added a more comprehensive description of previous studies on survival dynamics and changes at the transcriptomic level in the introduction:

> ~~Although not much is known about the mortality dynamics of starving bacteria, it~~ While bacterial survival has been observed ~~that~~ to exhibit simple exponential decay in certain conditions (Schink et al. 2019), other studies report an initial phase with almost no death (Dukan and Nyström 1999) and different bacterial species die at different rates and with diverse dynamics (Bergkessel and Delavaine 2021), suggesting that differences in both conditions and bacterial physiology can have a strong impact on mortality.

In addition, we added a dedicated paragraph on mortality dynamics reported in bulk and microfluidic experiments to the discussion section, together with a detailed comparison of these to the results we report here:

> Third, ~~with~~ we observed that the vast majority of cells survive during the 60 h of starvation in our experiment. This may seem contradictory with recent articles on *E. coli* starvation, in which the survival was reported to decrease exponentially from the onset of starvation (leading to ca. 50% mortality after 2 days; (Phaiboun et al. 2015; Schink et al. 2019)). However, it has been observed that survival dynamics depend on strains and conditions (Bergkessel and Delavaine 2021). In a previous study using the same strain (*E. coli* MG1655) and same conditions (M9 minimal media), it has been observed that survival curves measured in bulk only start to decay exponentially after several days (Dukan and Nyström 1999). Similarly, results from microfluidics experiments also report more than 90% survival during the first 60 h of starvation (Yang et al. 2023). Our observations are thus in agreement with previous reports. However, the differences in survival between strains and conditions calls for a more comprehensive study of the strain- and condition-dependence of *E. coli* survival under starvation.

The reviewer does not comment on these revisions but instead raises two additional references: Pavlou et al. 2025 as an example of single-cell tracking of fluorescence time series, and

Shimaya et al. 2021 for observations of reductive divisions. Respectfully, we do not feel that either of these references are particularly relevant.

Regarding Pavlou et al. 2025, there are quite a number of works by now on single-cell tracking of fluorescence time series and the Pavlou et al paper appeared only 9 months after our work was already on BioRxiv. Clearly Pavlou et al. 2025 played no role in the methodology used in our study. Second, the methodology for single-cell tracking and inference from fluorescence time series in this paper was mainly based on methodological developments by other groups. Third, the focus of the current work is not on methodological development but on the single-cell response to carbon starvation.

As to Shimaya et al. 2021, even though the authors refer to their switch as 'abrupt', the specifics of the microfluidic chip used in that study cause the E. coli cells to keep growing for several hours after the media is switched to remove nutrients (likely because nutrients persist for a significant amount of time). Therefore, the switch in this experiment is thus more akin to the gradual switch in the experiments of Bakshi et al. [1], which we already cite.

Response to Comment 2.4: *Thank you for the clarification. The concern raised here is not that transcriptional changes are unrelated to protein levels, but rather that the strength of the conclusions regarding proteome remodelling appears to extend beyond what can be directly supported by transcriptional reporter measurements and the associated model-based inference.*

While it is well established that transcription contributes to shaping protein abundances, protein levels in starvation conditions are also influenced by additional factors, including translation efficiency, protein stability, and degradation dynamics. These factors become particularly relevant in non-growing or energy-limited states, where turnover and resource constraints can substantially affect protein concentrations.

With regard to the proteomics measurements, the manuscript notes that these data are not sufficiently reproducible to provide reliable quantitative estimates of fold-changes in individual proteins. In this context, it is unclear to what extent they can serve as a strong validation of correspondence between reporter-based measurements and proteome-level dynamics. The reported qualitative agreement or overlap in trends, while suggestive, does not fully resolve this limitation.

The suggestion was therefore to more carefully qualify the use of the term proteome remodelling, or to frame the conclusions more explicitly in terms of transcriptional or reporter-based dynamics, unless stronger direct evidence of proteome-level changes is provided.

Second Response 2.4:

We agree with the reviewer that there are clear limitations to the accuracy with which the fold-changes of native proteins can be inferred from the transcriptional reporters. However, we believe that we took care to indicate these limitations in our manuscript. For example, in the revision we wrote:

> ~~Note that , because ribosome concentration is believed to be approximately constant independent of cell volume, and~~ We use the volumic production rate $q(t)$ as a proxy for the expression of the corresponding gene since it captures both the promoter-specific transcriptional activity as well as the global decrease of transcription and translation that occurs during starvation. In particular, because the fluorescent reporters of different promoters have essentially the same mRNA, the production rate $q(t)$ is proportional to the product of the ~~mRNA concentration of the reporter~~ reporter mRNA concentration and the total protein production rate per mRNA.

> We thus also aimed to track ~~how protein concentrations change across time for our promoters~~what our transcriptional reporter observations imply for the protein concentrations of the corresponding genes.
>
> To obtain time traces of protein concentration for ~~our fluorescent promoters~~ the genes corresponding to our reporters (Fig. 2A) we numerically integrated our estimates of the true time-dependent ~~protein production rates~~ production rates of fluorescent proteins $q(t)$ accounting also for the time-dependent protein degradation rate $\gamma(t)$ (~~SI section~~see Appendix section 3 for more details on the calculation). This approach relies on two main assumptions: that gene expression is primarily regulated at the transcriptional level in bacteria (Balakrishnan et al. 2022), i.e. translation and mRNA decay rates do not strongly differ across genes, and second that the rate of proteolysis of native proteins during starvation is similar to that of the fluorescent proteins.

For our second revision, we have gone through the manuscript and rephrased to systematically stress that we are using fluorescent reporters, that our degradation measurements are based on fluorescent reporters, and what the assumptions are upon which our inferences of protein concentration fold-changes rely. In particular:

> Here we combine microfluidics with quantitative fluorescence microscopy of transcriptional reporters to comprehensively quantify growth and gene expression at the single-cell level in *E. coli* during carbon starvation. (Abstract)
>
> In a previous study, it was reported that the expression of a transcriptional reporter of a synthetic inducible promoter exhibited constant gene expression, at $\approx 10\%$ of the level in exponentially growing cells, over 50 h in stationary phase (Gefen et al. 2014). (lines 102-105)
>
> Using separate measurements of protein degradation, we ~~found that protein degradation rates also decay~~ find that the rate at which fluorescent proteins are degraded itself decays exponentially with time~~and, using mathematical modeling, we infer~~. Assuming that this exponential decay of the degradation rate is a global feature that applies to other proteins as well, we developed a mathematical model that infers the dynamics of protein concentration ~~for each promoter and show~~ for each promoter. This model shows how protein concentrations in late starvation are determined by the combination of growth arrest (which halts dilution), the extent of transient increase in expression, the rate of exponential decay in protein production, and the exponential decay in protein degradation. (lines 131-140)
>
> and these uncovered that the protein degradation rate of the fluorescent proteins is decreasing approximately exponentially with time during the starvation phase, i.e. $\gamma(t) = \gamma_0 e^{-\omega_\gamma t}$, if we define ~~$t = 0$~~ $t = 0$ as the start of the starvation phase. ~~To infer the~~ (lines 208-211)
>
> This approach relies on two main assumptions~~: that gene expression is primarily regulated at the transcriptional level in bacteria (Balakrishnan et al. 2022), i.e. translation~~

and mRNA decay rates do not strongly differ across genes, and second . First, we assume that the rate of proteolysis degradation of native proteins during starvation is similar to that of the fluorescent proteins. As Second, the translational efficiency and mRNA stability of the fluorescent reporters may differ from that of the corresponding native proteins. However, what matters for the predicted protein concentration fold-changes is how the translational efficiency and mRNA stability change as the cells switch from exponential to stationary phase. As long as these changes are similar for the fluorescent reporters and native proteins, the predicted fold-changes will still be accurate.(lines 361-369)

However, although the proteomics measurements are not reproducible enough to quantify concentration fold-changes for individual proteins, we find that the trends are consistent between proteomics and Mother Machine measurementsthe proteomics measurements, and the measurements in the Mother Machine with fluorescent reporters. In particular, for almost all proteins the range of observed fold-changes in proteomics measurements overlaps with the fold-changes observed in fluorescence measurements of the reporters in the Mother Machine (Appendix section 7 and Fig. S12). This suggests that both the degradation rates as well as the changes in translation efficiency and mRNA decay cannot be too different between our fluorescent reporters and native proteins.(lines 391-400)

While the entry into growth arrest is clearly more gradual, the dynamics of protein production rates (Appendix Fig. S14C) as well as the inferred resulting dynamics of protein concentrations (lines 529-531)

Here we have shown that, contrary to the hypothesis that bacteria need to adapt their expression state before growth stops, marked phenotypic gene expression changes take place during the first 10 h of starvation. (lines 740-742)

Regarding the use of the term proteome remodeling, we note that 'remodeling' is a generic term and there is no debate that the proteome *is* in fact remodeled. Our proteomics experiments also demonstrate that the proteome is remodeled. Moreover, the trends seen in the fluorescent reporters match those of both transcriptomic and proteomic measurements. So we feel fully justified in claiming that our fluorescent reporter measurements are indicative of the proteome remodeling. The limitation is on the precise quantitative fold-changes and we fully acknowledge this in the manuscript.

Finally, the reviewer claims that factors like translation efficiency, protein stability, and degradation dynamics become particularly relevant in non-growing states, but provides no reference for this claim. We are not aware of any quantitative measurements supporting this claim. Instead, we feel that the fact that the transcriptomics and proteomics measurements (although noisy) are still consistent with our observations on fluorescent reporters demonstrates that the variation in translation efficiency and degradation cannot be a dominating effect.

Response to Comment 2.7: *Thank you for adding a dedicated Methods section to the main text.*

The intent of this comment was not to suggest that the information was entirely absent, but rather that its placement primarily in the supplementary material made it difficult to assess key

methodological aspects and their implications for data interpretation. The inclusion of a Methods section in the main manuscript significantly improves clarity and accessibility.

Second Response 2.7:

Although not relevant for our current revision, we feel that accuracy in the representation of the review process is important, and want to point out that, as can be easily checked, the reviewer did in fact explicitly state that this information was absent.

Response to Comment 2.8: *I appreciate that the authors intend to restrict their conclusions to similarities in gene expression dynamics between abrupt and gradual starvation. The concern raised here was primarily about how these results may be interpreted by readers, particularly in the context of broader discussion of adaptation mechanisms.*

Even when explicitly limited to transcriptional dynamics, similarities in reporter behaviour can be interpreted as implying a degree of functional or physiological equivalence, especially when these observations are discussed in the context of general adaptive responses to starvation. The suggestion was therefore to ensure that the manuscript clearly and consistently delineates the scope of this comparison and avoids potential overinterpretation beyond gene expression dynamics.

Second Response 2.8:

We feel that we were very careful about delineating the scope of the comparison of the abrupt and gradual switch already in our original manuscript. In particular, we wrote (bold added for emphasis):

> we also show that the ***gene expression response*** is similar between abrupt and gradual growth arrest.
>
> the dynamics of ***protein production rates*** (Appendix Fig. 14C) as well as the ***resulting*** dynamics of protein concentrations (Fig. 3C and Appendix Fig. 16) are very similar to those measured in experiments with a hard switch.

However, given that the reviewer expressed concern, we further clarified this point in the revision:

> This suggests that ~~our observations on gene expression in the experiments with instantaneous switching generalize to the entry into stationary phase in glucose-limited media, and probably beyond~~ the gene expression response upon loss of nutrients and cessation of growth is fairly independent of the speed of the switch and the precise amounts and types of residual carbon sources and may apply to a wide range of environmental transitions from growth to starvation.

More generally, the reviewer seems worried that, even though we do not make any claims beyond the similarity of the gene expression responses, readers might infer that the physiological outcomes are probably also the same (even though we have not explicitly established this and indeed also never claim this). We do not find this worry justified. Just because we haven't demonstrated that the physiological outcomes are also similar, does not mean that they are not. In fact, observing that the gene expression dynamics under abrupt and gradual starvation is very similar, it is entirely reasonable and parsimonious to assume that the physiological outcomes will also be similar. We don't think it is reasonable to demand that we preemptively warn readers against making such reasonable inferences.

Response to Comment 2.9: *Thank you for the clarification and for reorganising these comparisons. The intent of this comment was not to suggest that such controls were entirely absent, but rather that the use of multiple reporters with different properties was not presented in a sufficiently systematic way to clearly assess their impact on the reported dynamics. The inclusion of a consolidated comparison (Fig. S3), together with explicit correction for maturation kinetics, significantly improves the clarity of this aspect of the analysis.*

Second Response 2.9:

As with Comment 2.7, for accuracy of the record of the review process, we note that, as can be easily checked, the reviewer did claim these controls were absent.

Response to Comment 2.10: *Thank you for the clarification regarding the measurements of fluorescent protein degradation and their comparison to literature values.*

The primary concern raised here is not limited to the absolute degradation rate of fluorescent proteins, but rather the extent to which reporter-based measurements can support interpretations about protein turnover and resource allocation at the proteome level. Even if fluorescent protein degradation rates are of the same order of magnitude as native proteins on average, fluorescent proteins are generally relatively stable and are not subject to the same diversity of regulated degradation mechanisms that apply to native proteins.

As a result, reporter measurements do not capture potential gene-specific differences in degradation, translation efficiency, or mRNA dynamics. In particular, extrapolations from reporter-based degradation measurements to proteome-wide behaviour, and to quantitative estimates of resource availability, rely on assumptions that are not directly validated by the presented data.

The suggestion was therefore that interpretations linking the observed dynamics to mechanisms such as resource reallocation, endogenous metabolism, or proteolysis-driven energy supply would benefit from being clearly framed as hypotheses or plausible explanations, rather than as conclusions directly established by the data.

Second Response 2.10:

In the first round of review, the reviewer claimed that fluorescent proteins are more stable than native proteins. In response, we reviewed the literature extensively and explained that, as far as we are able to determine, there is no evidence in the literature to support the claim that fluorescent proteins have degradation dynamics that differs from that of native proteins. Given this, we find it hard to understand that the reviewer iterates this claim without providing a single supporting reference. We do not think there is any basis for the claim that fluorescent proteins are 'generally relatively stable' or that they are 'not subject to the same diversity of regulated degradation mechanisms'.

In the new revision, we have edited the corresponding paragraph of the Results to further prevent misunderstandings by the readers:

> Our measurements of the degradation of several fluorescent proteins during the first days of starvation (Appendix Fig. S2) shows that ~~the~~ their rate of protein degradation itself decays exponentially, and that on the order of half of the fluorescent proteins present at the onset of starvation are eventually degraded (Fig. 2).

With regard to transcriptional reporters not capturing 'possible gene-specific differences in degradation and translation efficiency', as discussed above, we have clearly specified these limitations.

Finally, we note that 'interpretations linking the observed dynamics to mechanisms such as resource allocation...', were in fact clearly presented as hypotheses and possible explanations, i.e. they were only discussed in the Discussion section and never presented as results.

Response to Comment 2.11: *Thank you for the clarification and for providing additional evidence regarding the expression of RpoS-regulated genes during exponential growth.*

The intent of this comment was to express surprise in light of the established literature showing rapid RpoS turnover during exponential phase, and therefore to highlight that the observed pre-starvation expression raises a mechanistic question. I did not mean to suggest that expression of these promoters in the exponential phase is impossible, but rather that it would benefit from clarification.

In particular, it would be helpful to discuss whether this signal reflects basal RpoS activity despite low steady-state levels, contributions from other regulatory inputs or sigma factors, or possible background effects in the fluorescence readout, such as cellular autofluorescence. Explicitly acknowledging these possibilities would strengthen the interpretation of these observations.

Second Response 2.11:

We first note that background fluorescence is explicitly corrected for in our analysis, so this cannot account for the observed signal. More importantly, our experiments with ΔrpoS strains directly address the mechanistic question raised by the reviewer: the residual promoter activity observed in the absence of RpoS demonstrates that this is not low level activity of RpoS but that other must regulatory inputs contribute to the expression of these promoters. In addition, the relative contributions of these other regulatory inputs must vary between promoters. Further investigation into what these other regulatory inputs are is beyond the scope of the present manuscript, and irrelevant for any of the results or interpretations that we present.

Response to Comment 2.17: *I appreciate the efforts made to address several of the points raised, in particular the incorporation of maturation-aware analysis of fluorescence dynamics, the clearer presentation of comparisons across different reporters, and the inclusion of a Methods section in the main text. These changes improve the clarity and technical robustness of the manuscript.*

The remaining concern relates primarily to the strength and scope of the conclusions relative to the data presented. In particular, interpretations concerning proteome remodelling, resource allocation, and broader adaptive mechanisms appear to extend beyond what can be directly supported by transcriptional reporter measurements and model-based inference alone. While the additional comparisons to proteomics and transcriptomics are useful for establishing qualitative consistency, they do not fully resolve these limitations at the level of quantitative or mechanistic interpretation.

Similarly, although the manuscript now clarifies that the comparison between abrupt and gradual starvation is limited to gene expression dynamics, care in framing remains important to avoid potential overinterpretation in terms of broader physiological equivalence.

Overall, I believe the manuscript presents a valuable and carefully executed dataset, and would be strengthened further by consistently aligning the framing of conclusions with the scope of the measurements.

Second Response 2.17:

In response to the first review of this reviewer, we invested great care to ensure the framing of our conclusions is fully aligned with the scope of our measurements. At this point, we must say

that we find it increasingly difficult to respond constructively to vague concerns that have now been raised several times across two rounds of review, each time without reference to a single specific passage in the manuscript. We have addressed these criticisms in detail in our responses to Comments 2.0, 2.2, 2.4, 2.8, and 2.10 and indicated how we have made further revisions to absolutely exclude the possibility that the framing of our conclusions extends beyond what our data provide. We therefore consider this concern fully addressed.

Comment 2.18: *I found that several of Reviewer 3's comments raise important experimental and interpretational questions that, in my view, are not fully addressed in the current responses. I summarise a few key points below that may be helpful in guiding further revision.*

Response to comment 3.2 (degree of starvation / nutrient carryover in microfluidics)

The reviewer's concern appears to be whether the microfluidic setup allows a sufficiently clean transition to nutrient-free conditions. In bulk experiments, done in the cited studies, cells are typically centrifuged, washed, and resuspended in fresh medium, which provides a relatively well-controlled removal of residual nutrients. In contrast, in microfluidic devices, cells reside in confined geometries (e.g. trenches), and residual medium may persist due to incomplete exchange or diffusion from surrounding materials (e.g. PDMS), potentially leading to trace nutrient availability over extended times.

The response focuses on variability in mortality reported in the literature and on the definition of "starvation," but does not directly address this physical aspect of the system. A more direct assessment (e.g. of media exchange efficiency, or controls comparing batch and device conditions under a hard switch) or a clearer discussion of this limitation would strengthen the interpretation.

Response 2.18:

The reviewer suggests that, switches to media without nutrients in mother machine devices are more subject to questions of remaining trace amounts of nutrients than bulk experiments, and mentions 'incomplete exchange' and 'diffusion from surrounding materials' as possible sources for remaining trace amounts of nutrients. Given the size of Avogadro's number, it seems almost certain that there are trace amounts of carbon source available, both in the mother machine and in bulk assays, but it would be technically very difficult to quantify these levels.

However, there is now an increasing body of work in mother machine devices where, upon a sudden switch of media, *all* cells immediately stop growing or completely halt expression of specific genes, e.g. [2]. In our opinion, such immediate and consistent responses are not consistent with either incomplete exchange or diffusion of previous media from the PDMS.

Nonetheless, in our manuscript we explicitly acknowledged the possibility of trace carbon nutrients remaining after the switch, showed that they do not qualitatively change the gene expression response (i.e. the similarity of responses in the abrupt and batch overflow switches), and made sure to define carbon starvation accordingly:

> Further, although it is impossible to exclude that trace amounts of carbon nutrients might still be present after the switch to M9 zero, it is clear that the immediate growth arrest in all cells is caused by the dramatic decrease in carbon availability upon the switch, and we will refer to this state as carbon starvation.

Comment 2.19: ***Response to comment 3.3 (phototoxicity)***

The revised analysis indicates a substantial phototoxicity effect at longer times (e.g. reduced regrowth after 60 h with illumination). While the authors argue that early dynamics are unaffected, the reviewer's concern is broader-namely, whether illumination may influence physiological state and mortality. The current phrasing ("we cannot exclude some effects") seems weaker than warranted given that measurable effects on viability are already observed. A more explicit acknowledgement of the magnitude of phototoxicity and its potential impact on interpretation would be appropriate.

Response 2.19:

The reviewer's comments suggest that they may have overlooked that we addressed the comment of reviewer 3 in the main text our revision:

> As an aside, comparison of the mortality between experiments with and without illumination shows some effects of phototoxicity late in starvation (Appendix Fig. S5A) and this raises the question whether gene expression late in starvation might be affected by phototoxicity as well. However, the strong correlation between expression in early and late starvation suggests that the effect of phototoxicity on gene expression cannot be too severe.

In the new revision, we further rephrased this paragraph and the paragraph in the Methods from which the reviewer cited:

> As an aside, ~~comparison of the mortality between~~ we compared mortality after 60 hours of starvation for experiments with and without illumination ~~shows some effects of phototoxicity late in starvation~~ and found that, while without illumination the large majority of cells survives, with constant illumination less than half of the cells survive (Appendix Fig. S5A)~~and this~~. This raises the question ~~whether gene expression late in starvation might be affected by phototoxicity as well. However, the strong correlation between expression~~ of whether phototoxicity may also affect gene expression. Although we cannot exclude some effects on gene expression, the fact that there is virtually no mortality after 25 hours with illumination, that most protein concentration changes occur during the first 10 hours, and that protein production in early and late starvation are well-correlated, suggests that the effect of phototoxicity on gene expression cannot be too severe.

> Although ~~we cannot exclude that~~ gene expression may be affected by phototoxicity, ~~since~~ we see no effects of fluorescence excitation on mortality for the first 25 hours~~and~~. Since most gene expression changes occur even earlier, i.e. after the first 5-10 hours of starvation ~~we see~~ there are no more significant changes in expression, we feel that it is unlikely phototoxicity is a major driver of the observed gene expression changes early in starvation.

Comment 2.20: ***Response to comment 3.4 (soft shift and residual carbon sources such as acetate)***

The reviewer raises a valid point that overflow from batch culture likely contains alternative carbon sources (e.g. acetate), as well as other changes associated with gradual nutrient depletion (e.g. pH shifts, accumulation of by-products). These factors can influence cellular physiology even in the absence of growth.

The response emphasises the similarity of gene expression dynamics between hard and soft shifts and proposes a model in which adaptation is driven primarily by internal resource turnover. However, this interpretation does not directly rule out contributions from residual external nutrients or environmental changes present in the soft-shift condition.

A more direct test, for example, introducing defined low concentrations of carbon sources in a controlled manner, or more explicitly discussing the limitations of the comparison, would help clarify the extent to which the observed dynamics are intrinsic versus environmentally driven.

More generally, across these responses, there is a recurring pattern in which concerns about experimental interpretation are addressed by comparison to literature or by reframing the question, rather than by directly engaging with the specific experimental limitations raised. Clarifying these points would, in my view, significantly improve the rigor and interpretability of the manuscript.

Response 2.20:

We completely agree with the reviewer that the overflow media from the batch culture are undoubtedly different from the M9 zero media used in the abrupt switch, and likely contain low-level nutrients that are by-products of the glucose metabolism. In fact, we explicitly point this out in our manuscript (lines 518-523). Second, we nowhere claim that these differences in media cannot affect the gene expression dynamics and show that the expression dynamics of the abrupt and gradual switch are not identical. However, the fact that the observed gene expression dynamics is very similar between the abrupt and gradual switch argues strongly that these differences in media are not a major determinant of the gene expression response. This too, we explicitly point out in the manuscript (lines 532-535).

Second, we do not agree that we 'present a model'. As mentioned above already, all we do is provide our interpretation of these observations in the Discussion section (i.e. proposing that the gene expression program is mainly driven by internal resource turnover). Notably, this proposed interpretation also explains our observation that, upon treatment with chloramphenicol during the first 5 hours, the gene expression response is still observed once the chloramphenicol is washed away. We feel that offering these interpretations in the Discussion section is entirely appropriate.

Finally, we respectfully reject the reviewer's suggestion that we are 'not engaging with the specific experimental limitations raised'. We feel that, on the contrary, we very diligently acknowledge the experimental limitations throughout the manuscript, as detailed in this long rebuttal.

References

[1] Somenath Bakshi, Emanuele Leoncini, Charles Baker, Silvia J. Cañas-Duarte, Burak Okumus, and Johan Paulsson. Tracking bacterial lineages in complex and dynamic environments with applications for growth control and persistence. *Nature Microbiology*, 6(6):783–791, June 2021.

[2] Thomas Julou, Ludovit Zweifel, Diana Blank, Athos Fiori, and Erik van Nimwegen. Subpopulations of sensorless bacteria drive fitness in fluctuating environments. *PLoS Biology*, 18(12):e3000952, December 2020. Number: 12.

18th May 2026

Manuscript Number: MSB-2025-13152RR
Title: E. coli prepares for starvation by dramatically remodeling its proteome in the first hours after loss of nutrients

Dear Dr Julou,

Thank you for the submission of your revised manuscript to Molecular Systems Biology. I am pleased to inform you that we will be able to accept your manuscript pending the following final amendments:

1) In the author checklist, you have indicated that human participants have been involved in the study, however this does not appear to be the case. If this is the case, please include in which section this information is available in the checklist, and additional ethics declarations will be needed. If human participants were not involved in the study, please update the author checklist to reflect this. Please also note that for the section on 'cell lines', we mainly refer to vertebrate cell lines, and you may indicate this section as not 'applicable'.
2) Please update all of the other parts of the author checklist in which you have selected 'yes' for Information included in the manuscript to indicate in which section that information has been given.
3) In the main manuscript file, please include keywords to max. 5.
4) Please deposit all mass spectrometry raw data to a suitable community repository (ProteomeXchange member repository for protein mass spectrometry data) and provide the accession codes in the Data Availability statement. Please ensure that the data are already publicly released.
5) Please rename "Competing interest statement" to "Disclosure and competing interests statement". We updated our journal's competing interests policy in January 2022 and request authors to consider both actual and perceived competing interests. Please review the policy https://link.springer.com/partners/embo-press/editorial-policies#Competing%20interest%20disclosures and update your competing interests if necessary.
6) Please correct the reference citation in the reference list such that when there are more than 10 authors on a paper, only the first 10 should be listed, followed by "et al.". In addition, DOIs should only be used for preprints and datasets that have not been published yet. Please check "Author Guidelines" for more information: https://link.springer.com/journal/44320/submission-guidelines#cms-Reference-guidelines
7) Our journal encourages inclusion of *data citations in the reference list* to directly cite datasets that were re-used and obtained from public databases. Data citations in the article text are distinct from normal bibliographical citations and should directly link to the database records from which the data can be accessed. In the main text, data citations are formatted as follows: "Data ref: Smith et al, 2001" or "Data ref: NCBI Sequence Read Archive PRJNA342805, 2017". In the Reference list, data citations must be labeled with "[DATASET]". A data reference must provide the database name, accession number/identifiers and a resolvable link to the landing page from which the data can be accessed at the end of the reference. Further instructions are available at: https://link.springer.com/journal/44320/submission-guidelines#cms-Reference-guidelines
8) Please place individual sections of the manuscript in the following order: Title page - Abstract & Keywords - Introduction - Results - Discussion - Methods - Data Availability - Acknowledgements - Disclosure and Competing Interests Statement - References - Figure Legends - Expanded View Figure Legends.
9) For the figures and figure legends, please take care of the following:
- Please note that information related to n is missing in the legends of figures 1E, 5B, 6B
- Please note that the error bars are not defined in the legends of figures 5B, 6B
10) Please remove the Supplementary Information section from the main manuscript file. If possible to include directly in the FASTA file, this would be preferred. Otherwise a description in the Methods with a callout to the file should also be okay. Please also update the filename and the callout for the file in the manuscript to Dataset EV1.
11) Please remove the Appendix from the main manuscript file and upload it as a separate single PDF with file type as Expanded View content. Please also ensure that the title page has a Table of Contents listing each item with page number. In addition, the nomenclature needs to be corrected to Appendix Figure S#, Appendix Table S# throughout the Appendix file and some of the callouts in the main manuscript also need to be updated to this nomenclature (Fig. S10A, Fig. S11, Fig. S12, Fig. S21C, Table S6). It is not necessary to submit the individual Appendix figures separately in a zip folder, these figures only need to be supplied in the Appendix PDF.
12) As part of the EMBO Publications transparent editorial process initiative (see our policy here: https://www.embopress.org/transparent-process#Review_Process), Molecular Systems Biology will publish online a Peer Review File (PRF) to accompany accepted manuscripts. This file will be published in conjunction with your paper and will include the anonymous referee reports, your point-by-point response and all pertinent correspondence relating to the manuscript. Let us know whether you agree with the publication of the PRF and as here, if you want to remove or not any figures from it prior to publication. Please note that the Authors checklist will be published at the end of the PRF.
13) After your paper is published, we may promote it on social media. If you have any handles or hashtags for Bluesky you would like included, please let us know.
14) Please provide a point-by-point letter INCLUDING my comments and your detailed responses (as Word file).

I look forward to reading a new revised version of your manuscript as soon as possible.

Yours sincerely,

Poonam Bheda, PhD
Scientific Editor
Molecular Systems Biology

University of Basel

BIOZENTRUM
The Center for Molecular Life Sciences

Poonam Bheda, PhD
Scientific Editor
Molecular Systems Biology

Basel, 26 May 2026
Minor revisions on manuscript MSB-2025-13152RR

Dear Dr Bheda

We are delighted that you accept our manuscript "*E. coli* prepares for starvation by dramatically remodeling its proteome in the first hours after loss of nutrients" after final amendments. Please find below our point-by-point response to your comments.

Yours sincerely

Thomas Julou, on behalf of all authors

University of Basel
Department Biozentrum
Spitalstrasse 41
4056 Basel, Switzerland
biozentrum.unibas.ch

Thomas Julou, PhD
Project Leader
T +41 61 207 16 21
thomas.julou@unibas.ch
biozentrum.unibas.ch/julou

1) In the author checklist, you have indicated that human participants have been involved in the study, however this does not appear to be the case. If this is the case, please include in which section this information is available in the checklist, and additional ethics declarations will be needed. If human participants were not involved in the study, please update the author checklist to reflect this. Please also note that for the section on 'cell lines', we mainly refer to vertebrate cell lines, and you may indicate this section as not 'applicable'.

We apologize for our error regarding human participants and modified the authors checklist following your recommendations.

2) Please update all of the other parts of the author checklist in which you have selected 'yes' for Information included in the manuscript to indicate in which section that information has been given.

We indicated the relevant section for each entry of the authors checklist.

3) In the main manuscript file, please include keywords to max. 5.

We added 5 keywords to the main manuscript file: carbon starvation, gene regulation, protein degradation, mother machine, stationary phase.

4) Please deposit all mass spectrometry raw data to a suitable community repository (ProteomeXchange member repository for protein mass spectrometry data) and provide the accession codes in the Data Availability statement. Please ensure that the data are already publicly released.

All mass spectrometry files associated with the experiments done for this manuscript were already accessible at MassIVE (accession code: MSV000095680) as indicated in the corresponding section of the Appendix. We now listed this resource and the corresponding DOI in the *Data availability* section.

5) Please rename "Competing interest statement" to "Disclosure and competing interests statement". We updated our journal's competing interests policy in January 2022 and request authors to consider both actual and perceived competing interests. Please review the policy https://link.springer.com/partners/embo-press/editorial-policies#Competing%20interest%20disclosures and update your competing interests if necessary.

We have updated this section accordingly.

6) Please correct the reference citation in the reference list such that when there are more than 10 authors on a paper, only the first 10 should be listed, followed by "et al.". In addition, DOIs should only be used for preprints and datasets that have not been published yet. Please check "Author Guidelines" for more information: https://link.springer.com/journal/44320/submission-guidelines#cms-Reference-guidelines

We edited the .bst file to comply with this formatting requirement, and fixed typographical issues in the references list.

*7) Our journal encourages inclusion of *data citations in the reference list* to directly cite datasets that were re-used and obtained from public databases. Data citations in the article text are distinct from normal bibliographical citations and should directly link to the database records from which the data can be accessed. In the main text, data citations are formatted as follows: "Data ref: Smith et al, 2001" or "Data ref: NCBI Sequence Read Archive PRJNA342805, 2017". In the Reference list, data citations must be*

labeled with "[DATASET]". A data reference must provide the database name, accession number/identifiers and a resolvable link to the landing page from which the data can be accessed at the end of the reference. Further instructions are available at: https://link.springer.com/journal/44320/submission-guidelines#cms-Reference-guidelines

We modified the references list accordingly (3 occurrences in the Appendix's references).

8) Please place individual sections of the manuscript in the following order: Title page - Abstract & Keywords - Introduction - Results - Discussion - Methods - Data Availability - Acknowledgements - Disclosure and Competing Interests Statement - References - Figure Legends - Expanded View Figure Legends.

The section order was updated accordingly.

9) For the figures and figure legends, please take care of the following:
- Please note that information related to n is missing in the legends of figures 1E, 5B, 6B
- Please note that the error bars are not defined in the legends of figures 5B, 6B

We now indicate the number of cells and the statistics indicated by the error bar.

10) Please remove the Supplementary Information section from the main manuscript file. If possible to include directly in the FASTA file, this would be preferred. Otherwise a description in the Methods with a callout to the file should also be okay. Please also update the filename and the callout for the file in the manuscript to Dataset EV1.

We now provide this file as Dataset EV1. We removed the section and updated the callout in the manuscript.

11) Please remove the Appendix from the main manuscript file and upload it as a separate single PDF with file type as Expanded View content. Please also ensure that the title page has a Table of Contents listing each item with page number. In addition, the nomenclature needs to be corrected to Appendix Figure S#, Appendix Table S# throughout the Appendix file and some of the callouts in the main manuscript also need to be updated to this nomenclature (Fig. S10A, Fig. S11, Fig. S12, Fig. S21C, Table S6). It is not necessary to submit the individual Appendix figures separately in a zip folder, these figures only need to be supplied in the Appendix PDF.

We fixed the callout to Appendix items in the Appendix itself.

12) As part of the EMBO Publications transparent editorial process initiative (see our policy here: https://www.embopress.org/transparent-process#Review_Process), Molecular Systems Biology will publish online a Peer Review File (PRF) to accompany accepted manuscripts. This file will be published in conjunction with your paper and will include the anonymous referee reports, your point-by-point response and all pertinent correspondence relating to the manuscript. Let us know whether you agree with the publication of the PRF and as here, if you want to remove or not any figures from it prior to publication. Please note that the Authors checklist will be published at the end of the PRF.

We agree with the publication of the PRF.

13) After your paper is published, we may promote it on social media. If you have any handles or hashtags for Bluesky you would like included, please let us know.

Please include the following handles:
@erikvannimwegen.bsky.social
@thomasjulou.bsky.social

14) Please provide a point-by-point letter INCLUDING my comments and your detailed responses (as Word file).

You are reading it.

9th Jun 2026
Manuscript number: MSB-2025-13152RRR
Title: E. coli prepares for starvation by dramatically remodeling its proteome in the first hours after loss of nutrients

Dear Dr Julou,

Thank you again for sending us your revised manuscript. We are now satisfied with the modifications made and I am pleased to inform you that your paper has been accepted for publication.

Your manuscript will be processed for publication by EMBO Press. It will be copy edited and you will receive page proofs prior to publication. Please note that you will be contacted by Springer Nature Author Services to complete licensing and payment information.

You may qualify for financial assistance for your publication charges - either via a Springer Nature fully open access agreement or an EMBO initiative. Check your eligibility: https://link.springer.com/journal/44320/how-to-publish-with-us

Should you be planning a Press Release on your article, please notify the production team via this form in order to coordinate publication and release dates: https://forms.cloud.microsoft/e/eH4A8JVP6g

If you have any questions, please do not hesitate to contact the Editorial Office. Thank you for your contribution to Molecular Systems Biology.

Yours sincerely,

Poonam Bheda, PhD
Scientific Editor
Molecular Systems Biology

>>> Please note that it is Molecular Systems Biology policy for the transcript of the editorial process (containing referee reports and your response letter) to be published as an online supplement to each paper. If you do NOT want this, you will need to inform the Editorial Office via email immediately. More information is available here: https://link.springer.com/partners/embo-press/editorial-policies#Peer%20review

EMBO Press Author Checklist

Corresponding Author Name: Thomas Julou
Journal Submitted to: Molecular Systems Biology
Manuscript Number: MSB-2025-13152-T

USEFUL LINKS FOR COMPLETING THIS FORM
The EMBO Journal - Author Guidelines
EMBO Reports - Author Guidelines
Molecular Systems Biology - Author Guidelines
EMBO Molecular Medicine - Author Guidelines

Reporting Checklist for Life Science Articles (updated January 2022)

This checklist is adapted from Materials Design Analysis Reporting (MDAR) Checklist for Authors. MDAR establishes a minimum set of requirements in transparent reporting in the life sciences (see Statement of Task: 10.31222/osf.io/9sm4x). Please follow the journal's guidelines in preparing your manuscript.
Please note that a copy of this checklist will be published alongside your article.

Abridged guidelines for figures
1. Data
The data shown in figures should satisfy the following conditions:
- the data were obtained and processed according to the field's best practice and are presented to reflect the results of the experiments in an accurate and unbiased manner.
- ideally, figure panels should include only measurements that are directly comparable to each other and obtained with the same assay.
- plots include clearly labeled error bars for independent experiments and sample sizes. Unless justified, error bars should not be shown for technical replicates.
- if n<5, the individual data points from each experiment should be plotted. Any statistical test employed should be justified.
- Source Data should be included to report the data underlying figures according to the guidelines set out in the authorship guidelines on Data Presentation.

2. Captions
Each figure caption should contain the following information, for each panel where they are relevant:
- a specification of the experimental system investigated (eg cell line, species name).
- the assay(s) and method(s) used to carry out the reported observations and measurements.
- an explicit mention of the biological and chemical entity(ies) that are being measured.
- an explicit mention of the biological and chemical entity(ies) that are altered/varied/perturbed in a controlled manner.
- the exact sample size (n) for each experimental group/condition, given as a number, not a range;
- a description of the sample collection allowing the reader to understand whether the samples represent technical or biological replicates (including how many animals, litters, cultures, etc.).
- a statement of how many times the experiment shown was independently replicated in the laboratory.
- definitions of statistical methods and measures:
  - common tests, such as t-test (please specify whether paired vs. unpaired), simple χ2 tests, Wilcoxon and Mann-Whitney tests, can be unambiguously identified by name only, but more complex techniques should be described in the methods section;
  - are tests one-sided or two-sided?
  - are there adjustments for multiple comparisons?
  - exact statistical test results, e.g., P values = x but not P values < x;
  - definition of 'center values' as median or average;
  - definition of error bars as s.d. or s.e.m.

Please complete ALL of the questions below.
Select "Not Applicable" only when the requested information is not relevant for your study.

Materials

Newly Created Materials	Information included in the manuscript?	In which section is the information available? (Reagents and Tools Table, Materials and Methods, Figures, Data Availability Section)
New materials and reagents need to be available; do any restrictions apply?	Yes	Reagents and Tools Table, Material and Methods, Figures and Data Availability Section

Antibodies	Information included in the manuscript?	In which section is the information available? (Reagents and Tools Table, Materials and Methods, Figures, Data Availability Section)
For **antibodies** provide the following information:<br>- Commercial antibodies: RRID (if possible) or supplier name, catalogue number and or/clone number<br>- Non-commercial: RRID or citation	Not Applicable	

DNA and RNA sequences	Information included in the manuscript?	In which section is the information available? (Reagents and Tools Table, Materials and Methods, Figures, Data Availability Section)
**Short novel DNA or RNA including primers, probes**: provide the sequences.	Yes	Reagents and Tools Table, Material and Methods, Dataset EV1

Cell materials	Information included in the manuscript?	In which section is the information available? (Reagents and Tools Table, Materials and Methods, Figures, Data Availability Section)
**Cell lines:** Provide species information, strain. Provide accession number in repository **OR** supplier name, catalog number, clone number, and/**OR** RRID.	Not Applicable	
**Primary cultures:** Provide species, strain, sex of origin, genetic modification status.	Not Applicable	
Report if the cell lines were recently **authenticated** (e.g., by STR profiling) and tested for mycoplasma contamination.	Not Applicable	

Experimental animals	Information included in the manuscript?	In which section is the information available? (Reagents and Tools Table, Materials and Methods, Figures, Data Availability Section)
**Laboratory animals or Model organisms:** Provide species, strain, sex, age, genetic modification status. Provide accession number in repository **OR** supplier name, catalog number, clone number, **OR** RRID.	Not Applicable	
**Animal observed in or captured from the field:** Provide species, sex, and age where possible.	Not Applicable	
Please detail **housing and husbandry conditions**.	Not Applicable	

Plants and microbes	Information included in the manuscript?	In which section is the information available? (Reagents and Tools Table, Materials and Methods, Figures, Data Availability Section)
**Plants:** provide species and strain, ecotype and cultivar where relevant, unique accession number if available, and source (including location for collected wild specimens).	Not Applicable	
**Microbes:** provide species and strain, unique accession number if available, and source.	Yes	Reagents and Tools Table and Material and Methods

Human research participants	Information included in the manuscript?	In which section is the information available? (Reagents and Tools Table, Materials and Methods, Figures, Data Availability Section)
If collected and within the bounds of privacy constraints report on age, sex and gender or ethnicity for all study participants.	Not Applicable	

Core facilities	Information included in the manuscript?	In which section is the information available? (Reagents and Tools Table, Materials and Methods, Figures, Data Availability Section)
If your work benefited from core facilities, was their service mentioned in the acknowledgments section?	Yes	Acknowledgments section

Design

Study protocol	Information included in the manuscript?	In which section is the information available? (Reagents and Tools Table, Materials and Methods, Figures, Data Availability Section)
If study protocol has been **pre-registered, provide DOI in the manuscript**. For clinical trials, provide the trial registration number **OR** cite DOI.	Not Applicable	
Report the **clinical trial registration number** (at ClinicalTrials.gov or equivalent), where applicable.	Not Applicable	

Laboratory protocol	Information included in the manuscript?	In which section is the information available? (Reagents and Tools Table, Materials and Methods, Figures, Data Availability Section)
Provide DOI OR other citation details if **external detailed step-by-step protocols** are available.	Not Applicable	

Experimental study design and statistics	Information included in the manuscript?	In which section is the information available? (Reagents and Tools Table, Materials and Methods, Figures, Data Availability Section)
Include a statement about **sample size** estimate even if no statistical methods were used.	Yes	Material and Methods and Figures
Were any steps taken to minimize the effects of subjective bias when allocating animals/samples to treatment (e.g. **randomization procedure**)? If yes, have they been described?	Not Applicable	
Include a statement about **blinding** even if no blinding was done.	Not Applicable	
Describe **inclusion/exclusion criteria** if samples or animals were excluded from the analysis. Were the criteria pre-established?<br><br>If sample or data points were omitted from analysis, report if this was due to attrition or intentional exclusion and provide justification.	Yes	Material and Methods and Figures
For every figure, are **statistical tests** justified as appropriate? Do the data meet the assumptions of the tests (e.g., normal distribution)? Describe any methods used to assess it. Is there an estimate of variation within each group of data? Is the variance similar between the groups that are being statistically compared?	Yes	Material and Methods and Figures

Sample definition and in-laboratory replication	Information included in the manuscript?	In which section is the information available? (Reagents and Tools Table, Materials and Methods, Figures, Data Availability Section)
In the figure legends: state number of times the experiment was **replicated** in laboratory.	Yes	Figures
In the figure legends: define whether data describe **technical or biological replicates**.	Yes	Figures

Ethics

Ethics	Information included in the manuscript?	In which section is the information available? (Reagents and Tools Table, Materials and Methods, Figures, Data Availability Section)
Studies involving **human participants**: State details of **authority granting ethics approval** (IRB or equivalent committee(s), provide reference number for approval.	Not Applicable	
Studies involving **human participants**: Include a statement confirming that **informed consent** was obtained from all subjects and that the experiments conformed to the principles set out in the WMA Declaration of Helsinki and the Department of Health and Human Services Belmont Report.	Not Applicable	
Studies involving **human participants:** For publication of **patient photos**, include a statement confirming that consent to publish was obtained.	Not Applicable	
Studies involving experimental **animals**: State details of **authority granting ethics approval** (IRB or equivalent committee(s), provide reference number for approval. Include a statement of compliance with ethical regulations.	Not Applicable	
Studies involving **specimen and field samples:** State if relevant **permits** obtained, provide details of authority approving study; if none were required, explain why.	Not Applicable	

Dual Use Research of Concern (DURC)	Information included in the manuscript?	In which section is the information available? (Reagents and Tools Table, Materials and Methods, Figures, Data Availability Section)
Could your study fall under dual use research restrictions? Please check biosecurity documents and list of select agents and toxins (CDC): https://www.selectagents.gov/sat/list.htm	Not Applicable	
If you used a select agent, is the security level of the lab appropriate and reported in the manuscript?	Not Applicable	
If a study is subject to dual use research of concern regulations, is the name of the **authority granting approval and reference number** for the regulatory approval provided in the manuscript?	Not Applicable	

Reporting

The MDAR framework recommends adoption of discipline-specific guidelines, established and endorsed through community initiatives. Journals have their own policy about requiring specific guidelines and recommendations to complement MDAR.

Adherence to community standards	Information included in the manuscript?	In which section is the information available? (Reagents and Tools Table, Materials and Methods, Figures, Data Availability Section)
State if relevant guidelines or checklists (e.g., **ICMJE, MIBBI, ARRIVE, PRISMA**) have been followed or provided.	Not Applicable	
For **tumor marker prognostic studies,** we recommend that you follow the **REMARK** reporting guidelines (see link list at top right). See author guidelines, under 'Reporting Guidelines'. Please confirm you have followed these guidelines.	Not Applicable	
For **phase II and III randomized controlled trials**, please refer to the **CONSORT** flow diagram (see link list at top right) and submit the CONSORT checklist (see link list at top right) with your submission. See author guidelines, under 'Reporting Guidelines'. Please confirm you have submitted this list.	Not Applicable	

Data Availability

Data availability	Information included in the manuscript?	In which section is the information available? (Reagents and Tools Table, Materials and Methods, Figures, Data Availability Section)
Have **primary datasets** been deposited according to the journal's guidelines (see 'Data Deposition' section) and the respective accession numbers provided in the Data Availability Section?	Yes	Data Availability
Were **human clinical and genomic datasets** deposited in a public access-controlled repository in accordance to ethical obligations to the patients and to the applicable consent agreement?	Not Applicable	
Are **computational models** that are central and integral to a study available without restrictions in a machine-readable form? Were the relevant accession numbers or links provided?	Not Applicable	
If publicly available data were reused, provide the respective **data citations in the reference list**.	Not Applicable	
